# ﻿Mayflies of the Fiji Islands (Ephemeroptera)

**DOI:** 10.3897/zookeys.1259.168521

**Published:** 2025-11-11

**Authors:** Thomas Kaltenbach

**Affiliations:** 1 Muséum cantonal des Sciences Naturelles, Département de zoologie, Palais de Rumine, Place Riponne 6, CH-1005 Lausanne, Switzerland Muséum cantonal des Sciences Naturelles Lausanne Switzerland; 2 University of Lausanne (UNIL), Department of Ecology and Evolution, CH-1015 Lausanne, Switzerland University of Lausanne Lausanne Switzerland

**Keywords:** *
Baetis
*, COI, *
Fijifiliola
*, integrative taxonomy, ovoviviparity, *
Papuanatula
*, parthenogenesis

## Abstract

Material collected in 2024 in nine different rivers on the two main islands of Fiji, Viti Levu and Vanua Levu, is the basis for this taxonomic study of mayflies of the Fiji Islands. Apart from one larva of Caenidae, not treated in this study, all other larvae and three female imagos belong to two genera of the family Baetidae (*Baetis* Leach and *Papuanatula* Lugo-Ortiz & McCafferty). A new subgenus of *Papuanatula*, *Fijifiliola***subgen. nov.**, based on the larvae of ten new species, and the larva and female imago of one new species of *Baetis* are described and illustrated. DNA barcodes (COI) were obtained for most species, and their genetic distances were analysed (Kimura-2 parameter). A key to all species of Papuanatula (Fijifiliola)**subgen. nov.** is provided. Amongst the new species of Papuanatula (Fijifiliola)**subgen. nov.**, a case of parthenogenesis and likely ovoviviparity is discussed.

## ﻿Introduction

The Fijian archipelago in the southwestern Pacific consists of more than 300 islands with a tropical, maritime climate, laying in the middle of other archipelagos, namely Vanuatu, New Caledonia, Tonga, and Samoa. It has four major islands, Viti Levu, Vanua Levu, Taveuni, and Kadavu. In terms of geology, Fiji is dominated by volcanic rocks and their erosion products (Colley 2009). Fauna and Flora of the Fiji Islands are strongly connected to New Guinea. Fiji is close enough to other island groups for dispersal to occur, likely along the Melanesian arc, which had even more islands in the past and therefore, enabled shorter dispersal distances (Flowers 1990; Ryan 2009). Later, some of the western islands of the Melanesian arc converged to become part of what is today New Guinea. This likely dispersal together with a wide range of habitats has led to a considerable diversity in the Fiji Islands (Ryan 2009).

The mayfly fauna of the Fiji Islands is poorly studied. As explained in Flowers (1990), McLean (1974) published a study guide to aquatic insects of the Fiji Islands, intended for students, without formal taxonomic treatment. He listed the mayfly genera *Baetis*, *Pseudocloeon*, and *Isca* (Leptophlebiidae). According to Flowers (1990), *Baetis* refers to species of the former group *molawinensis* (according to Müller-Liebenau and Hubbard 1985), which later became *Labiobaetis* Novikova & Kluge, 1987; *Pseudocloeon* refers to New Genus “A”, which is described in this study as Papuanatula (Fijifiliola) subgen. nov.; and *Isca*, which was based on adults only, refers to a species of Caenidae. The study of Flowers (1990) on Ephemeroptera of the Fiji Islands gives an important first view on the diversity, morphology, and biogeography of mayflies on this archipelago, but without any formal taxonomic treatment. His study was mainly based on very rich material coming from Dr. McLean’s collections in Fiji and from the University of the South Pacific’s Institute of Natural Resources Fijian aquatic insect survey in 1980. Sixty locations on four different islands (Viti Levu, Vanua Levu, Ovalau, and Ono-i-Kadavu) were covered, but most specimens came from the two largest islands, Viti Levu and Vanua Levu. Flowers (1990) reported one species of Caenidae, and 14 species of Baetidae from three genera: *Baetis* group *molawinensis* (according to Müller-Liebenau and Hubbard 1985, now *Labiobaetis*) with three species; New Genus “A” described herein as Papuanatula (Fijifiliola) subgen. nov. with eight species; and New Genus “B” (probably undescribed new genus; no material available for this study) with three species. He also provided figures of main characters of the different genera. Additionally, some ecological studies on streams were done on the main island Viti Levu by Haynes (1987, 1994, 1999). Haynes (1999) mentioned three species of Ephemeroptera: *Cloeon* sp. A, *Cloeon* sp. B, and *Pseudocloeon* sp., the latter as living on stones and boulders. *Pseudocloeon* certainly refers to *Papuanatula*, but it remains unclear to what *Cloeon* refers. Neither McLean (1974) nor Flowers (1990) mentioned *Cloeon* for the Fiji Islands, but this does not exclude their presence. *Cloeon* has a worldwide distribution, some species are successful colonisers, and its presence on Vanuatu has been confirmed (Gattolliat and Staniczek 2011). The Bishop Museum of Hawaii provided a checklist of Ephemeroptera of Fiji compiled by Evenhuis (2007), in which all the above-mentioned species are listed.

Currently, the genus *Papuanatula* Lugo-Ortiz & McCafferty, 1999 (Baetidae) comprises 33 species, 32 endemic to New Guinea including the island of New Britain, and one species from Sulawesi (Lugo-Ortiz and McCafferty 1999; Kaltenbach et al. 2025a, b). In this study, ten new species of *Papuanatula* are described and illustrated based on larvae, all belonging to the new subgenus Fijifiliola subgen. nov., which is also described. The material was collected by the author in 2024 in nine rivers on Viti Levu and Vanua Levu (Table 2). Considering the high diversity of *Papuanatula* in the Fiji Islands and the limited locations investigated so far, we must expect further new species in the future, also when the historical material from this archipelago is studied in more detail.

Surprisingly, a new species of *Baetis* was also discovered, far from the closest known occurrences of the genus in Southeast Asia, Taiwan, or Japan; currently, the genus is not known from New Guinea. Larvae and female imagos of the new species are described and illustrated. Collections in surrounding archipelagos, especially toward New Guinea, may show if it is an isolated population in the Fiji Islands, or somehow connected to other records of the genus.

## ﻿Materials and methods

The larvae and three female imagos were collected by kick-sampling and preserved in 96% ethanol. Dissection of larvae and imagos was done in Cellosolve (2-Ethoxyethanol) with subsequent mounting on slides with Euparal liquid, using an Olympus SZX7 stereomicroscope.

The DNA of part of the specimens was extracted using non-destructive methods allowing subsequent morphological analysis (see Vuataz et al. 2011 for details). We amplified a 658 bp fragment of the mitochondrial gene cytochrome oxidase subunit 1 (COI) using the primers LCO 1490 and HCO 2198 (Folmer et al. 1994). Sequencing was done with Sanger’s method (Sanger et al. 1977). Genetic variability between specimens was estimated using Kimura-2-parameter distances (K2P, Kimura 1980), calculated with the program MEGA 11 (Tamura et al. 2021, http://www.megasoftware.net).

The GenBank accession numbers are given in Table 1; nomenclature of gene sequences follows Chakrabarty et al. (2013).

**Table 1.** Sequenced specimens (COI).

Species	Specimen voucher catalogue #	Island	Locality code	GPS coordinates	GenBank # (COI)	GenSeq nomenclature
P. (F.) aji sp. nov.	GBIFCH00975846	Viti Levu	A	17°37'35"S, 177°45'04"E	PX067251	genseq-2 COI
	GBIFCH00975847	Viti Levu	A	17°37'35"S, 177°45'04"E	PX067252	genseq-2 COI
	GBIFCH01581961	Viti Levu	B	17°37'33"S, 177°45'15"E	PX067253	genseq-2 COI
	GBIFCH01581939	Viti Levu	C	17°40'26"S, 177°33'06"E	PX067254	genseq-2 COI
	GBIFCH01581953	Viti Levu	C1	17°40'07"S, 177°32'31"E	PX067255	genseq-2 COI
	GBIFCH01581925	Viti Levu	C1	17°40'07"S, 177°32'31"E	PX067256	genseq-2 COI
	GBIFCH01582018	Viti Levu	C1	17°40'07"S, 177°32'31"E	PX067257	genseq-2 COI
	GBIFCH01582021	Viti Levu	C1	17°40'07"S, 177°32'31"E	PX067258	genseq-2 COI
	GBIFCH01582022	Viti Levu	C1	17°40'07"S, 177°32'31"E	PX067259	genseq-2 COI
	GBIFCH01582024	Viti Levu	C1	17°40'07"S, 177°32'31"E	PX067260	genseq-2 COI
	GBIFCH01581917	Viti Levu	D	17°38'12"S, 177°45'29"E	PX067262	genseq-2 COI
	GBIFCH01581918	Viti Levu	D	17°38'12"S, 177°45'29"E	PX067263	genseq-2 COI
	GBIFCH01581976	Viti Levu	I	17°43'09"S, 177°31'21"E	PX067270	genseq-2 COI
	GBIFCH01581977	Viti Levu	I	17°43'09"S, 177°31'21"E	PX067268	genseq-2 COI
	GBIFCH01581978	Viti Levu	I	17°43'09"S, 177°31'21"E	PX067269	genseq-2 COI
	GBIFCH01582028	Viti Levu	I	17°43'09"S, 177°31'21"E	PX067261	genseq-2 COI
	GBIFCH01581954	Vanua Levu	E	16°39'55"S, 179°20'03"E	PX067264	genseq-2 COI
	GBIFCH01581909	Vanua Levu	E1	16°39'54"S, 179°20'01"E	PX067265	genseq-2 COI
	GBIFCH01581987	Vanua Levu	F	16°36'25"S, 179°08'46"E	PX067267	genseq-2 COI
	GBIFCH01581986	Vanua Levu	F	16°36'25"S, 179°08'46"E	PX067266	genseq-2 COI
P. (F.) claudia sp. nov.	GBIFCH01581930	Viti Levu	C	17°40'26"S, 177°33'06"E	PX067280	genseq-2 COI
	GBIFCH01581934	Viti Levu	C	17°40'26"S, 177°33'06"E	PX067279	genseq-2 COI
P. (F.) crussetae sp. nov.	GBIFCH01582032	Viti Levu	C	17°40'26"S, 177°33'06"E	PX067281	genseq-1 COI
P. (F.) sekawa sp. nov.	GBIFCH01581904	Vanua Levu	E	16°39'55"S, 179°20'03"E	PX067294	genseq-2 COI
	GBIFCH01581903	Vanua Levu	E	16°39'55"S, 179°20'03"E	PX067293	genseq-2 COI
	GBIFCH01581908	Vanua Levu	E	16°39'55"S, 179°20'03"E	PX067292	genseq-2 COI
P. (F.) gattolliati sp. nov.	GBIFCH01581907	Vanua Levu	E	16°39'55"S, 179°20'03"E	PX067284	genseq-2 COI
	GBIFCH01581989	Vanua Levu	F	16°36'25"S, 179°08'46"E	PX067285	genseq-2 COI
P. (F.) grisea sp. nov.	GBIFCH01581940	Viti Levu	A	17°37'35"S, 177°45'04"E	PX067286	genseq-2 COI
	GBIFCH01582011	Viti Levu	A	17°37'35"S, 177°45'04"E	PX067288	genseq-2 COI
	GBIFCH01582029	Viti Levu	A	17°37'35"S, 177°45'04"E	PX067287	genseq-2 COI
	GBIFCH01581958	Viti Levu	B	17°37'33"S, 177°45'15"E	PX067289	genseq-2 COI
	GBIFCH01582026	Viti Levu	I	17°43'09"S, 177°31'21"E	PX067291	genseq-2 COI
	GBIFCH01581972	Viti Levu	I	17°43'09"S, 177°31'21"E	PX067290	genseq-2 COI
P. (F.) flowersi sp. nov.	GBIFCH01581952	Viti Levu	C1	17°40'07"S, 177°32'31"E	PX067283	genseq-2 COI
	GBIFCH01581932	Viti Levu	C	17°40'26"S, 177°33'06"E	PX067282	genseq-2 COI
P. (F.) bula sp. nov.	GBIFCH01581915	Viti Levu	D1	17°38'22"S, 177°45'30"E	PX067271	genseq-2 COI
	GBIFCH01581981	Viti Levu	G	18°10'57"S, 177°44'13"E	PX067272	genseq-2 COI
	GBIFCH01581982	Viti Levu	G	18°10'57"S, 177°44'13"E	PX067273	genseq-2 COI
	GBIFCH01581980	Viti Levu	G	18°10'57"S, 177°44'13"E	PX067274	genseq-2 COI
	GBIFCH01582014	Viti Levu	G	18°10'57"S, 177°44'13"E	PX067275	genseq-2 COI
	GBIFCH01581920	Viti Levu	H	18°03'35"S, 178°28'11"E	PX067276	genseq-2 COI
	GBIFCH01581956	Viti Levu	H	18°03'35"S, 178°28'11"E	PX067278	genseq-2 COI
	GBIFCH01581955	Viti Levu	H	18°03'35"S, 178°28'11"E	PX067277	genseq-2 COI
*B. procul* sp. nov.	GBIFCH01581965	Viti Levu	B	17°37'33"S, 177°45'15"E	PX067250	genseq-2 COI
	GBIFCH01581967	Viti Levu	I	17°43'09"S, 177°31'21"E	PX067247	genseq-2 COI
	GBIFCH01581968	Viti Levu	I	17°43'09"S, 177°31'21"E	PX067248	genseq-2 COI
	GBIFCH01581969	Viti Levu	I	17°43'09"S, 177°31'21"E	PX067249	genseq-2 COI

Photographs of larvae in toto were taken using a Canon EOS 6D camera and processed with the programs Adobe Photoshop Lightroom (http://www.adobe.com) and Helicon Focus v. 5.3 (http://www.heliconsoft.com). Photographs of larval and imaginal parts were taken with an Olympus SC 50 camera on an Olympus BX43 microscope, processed with the program Olympus Cell Sense v. 3.2. Photographs were subsequently enhanced with Adobe Photoshop Elements 13.

Distribution maps were generated with the program SimpleMappr (https://simplemappr.net, Shorthouse 2010). Coordinates and description of sample locations are given in Table 2.

**Table 2.** Sample locations (Figs 36–38; w: width, d: depth).

Location code	Island	GPS coordinates	Altitude	River	Characteristics (surroundings/size/velocity/riv. bed)
A	Viti Levu	17°37'35"S, 177°45'04"E	20 m	Ba River	farmland, secondary forest
					w: 20 m; d: 0.1–0.3 m
					medium fast
					mainly cobble
B	Viti Levu	17°37'33"S, 177°45'15"E	20 m	Ba River	farmland, secondary forest
					w: 20 m; d: 0.1–0.3 m
					medium fast
					mainly cobble
C	Viti Levu	17°40'26"S, 177°33'06"E	690 m	Savuione River	primary rainforest, pristine
					narrow river, close to waterfall
					very fast, turbulent
					bedrock
C1	Viti Levu	17°40'07"S, 177°32'31"E	510 m	Savuione River	secondary rainforest, pristine
					narrow, shallow runs on rock surface
					fast, turbulent
					bedrock
D	Viti Levu	17°38'12"S, 177°45'29"E	160 m	tributary of Ba River	farmland, open grassland, disturbed
					w: 0.5–1 m; d: 0.1–0.2 m
					slow
					muddy, algae, stones, cobble
D1	Viti Levu	17°38'22"S, 177°45'30"E	220 m	tributary of Ba River	farmland, open grassland, disturbed
					w: 0.5–1 m; d: 0.1–0.2 m
					slow
					muddy, algae, stones, cobble
E	Vanua Levu	16°39'55"S, 179°20'03"E	30 m	tributary of Sekawa River	rainforest, pristine
					w: 5 m; d: 0.2 m
					medium fast
					mainly cobble
E1	Vanua Levu	16°39'54"S, 179°20'01"E	30 m	Sekawa River	forest, open grassland, disturbed
					w: 10 m; d: 0.2 m
					medium fast
					cobble, sand, filamentous algae cover
F	Vanua Levu	16°36'25"S, 179°08'46"E	80 m	Seaqaqa River	open forest, disturbed
					w: 5 m; d: 0.1–0.2 m
					medium fast
					cobble, gravel
G	Viti Levu	18°10'57"S, 177°44'13"E	70 m	Biausevu River	rainforest, closed canopy; pristine
					w: 3 m; d: 0.1–0.2 m
					medium fast
					stones, sandy patches
H	Viti Levu	18°03'35"S, 178°28'11"E	160 m	Waisila Creek	prim. rainforest, closed canopy; pristine
					w: 3–5 m; d: 0.1–0.5 m
					fast
					stones, cobble, sand
I	Viti Levu	17°43'09"S, 177°31'21"E	30 m	Sabeto River	farmland, open forest, wide banks
					w: 10 m; d: 0.1–0.4 m
					medium fast
					stones, cobble, gravel

The dichotomous key was elaborated with support of the program DKey v. 1.3.0 (http://drawwing.org/dkey, Tofilski 2018).

The terminology follows Kluge (2004). The term “blank” is used to describe an unpigmented area of cuticle (Kluge et al. 2023). The term “posterior seta/setae” is used for long setae in posterior position of the claw (approximately opposite to the distalmost denticle), as proposed by Kluge and Novikova (2014).

All material has been deposited in the Muséum cantonal des Sciences Naturelles, Lausanne, Switzerland (**MZL**).

## ﻿Results

### ﻿Genus *Baetis* Leach, 1815

#### ﻿ Baetis procul

Taxon classificationAnimaliaEphemeropteraBaetidae

sp. nov.

6019203B-2827-5126-925C-7B3160E21525

https://zoobank.org/0C33CE39-3BFE-4C55-8ADB-AAECDAD8135A

Figs 1, 2, 3, 4

##### Material examined.

***Holotype*.** FIJI • larva; Viti Levu, Ba Prov., Sabeto Riv., near Sabeto; 17°43'09"S, 177°31'21"E; 30 m; 17.xi.2024; leg. T. Kaltenbach; on slides; GBIFCH01221812, GBIFCH01221813; MZL. ***Paratypes*.** • 11 larvae; same data as holotype; 2 on slides; GBIFCH01221839, GBIFCH01581967, GBIFCH01581968; 9 in alcohol; GBIFCH01581966, GBIFCH01581969, GBIFCH01581970; MZL • 3 female imagos; Viti Levu, Ba Prov., Ba Riv., near Outback Hotel; 17°37'35"S, 177°45'04"E; 20 m; 22.x.2024; leg. T. Kaltenbach; 1 on slide; GBIFCH01221835; 2 in alcohol; GBIFCH01581964, GBIFCH01581965; MZL.

##### Diagnosis.

**Larva.** Following combination of characters differentiate *Baetis procul* sp. nov. from other species of *Baetis*, some of the characters also from the most similar species *Baetis taiwanensis* Müller-Liebenau, 1985: tergalii present on abdominal segments I–VII; hind protopteron developed; abdominal terga I–VIII grey-brown, with distinct sublateral pale areas; femur with yellowish to yellow-brown, subapical spot on anterior side.

##### Description.

**Figure 1.**
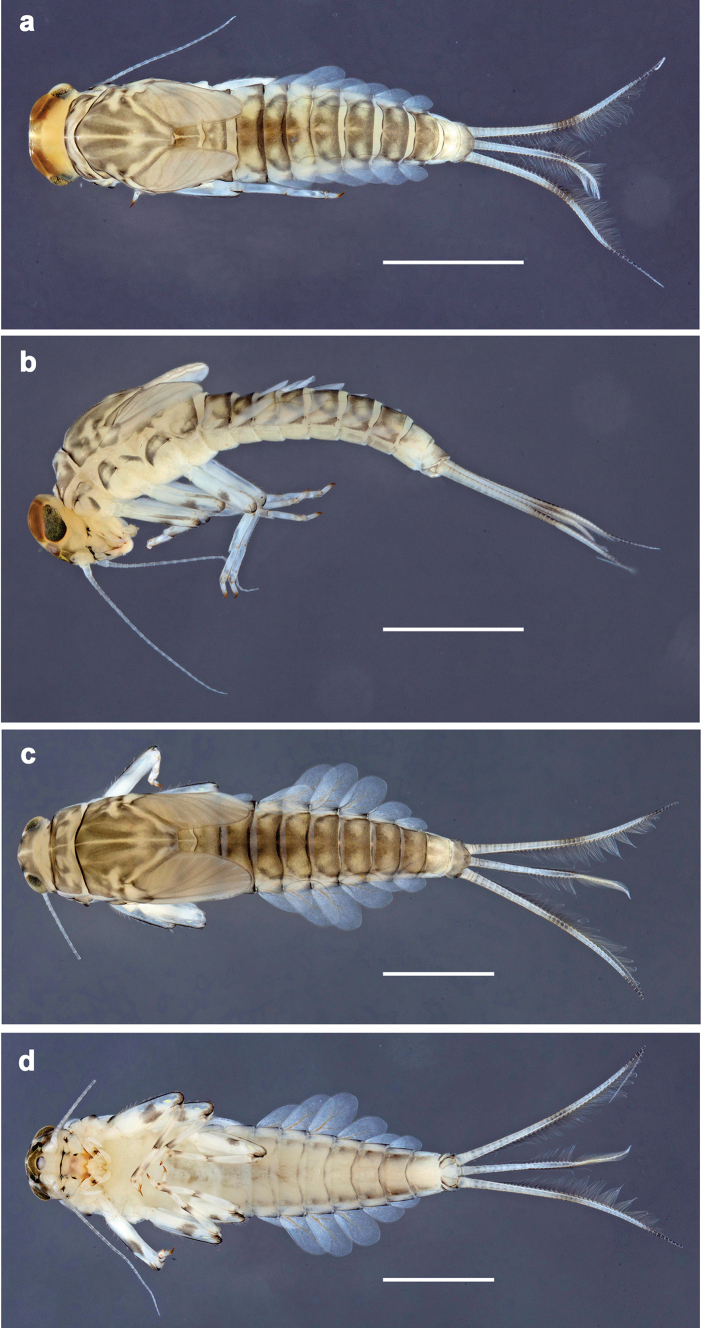
*Baetis procul* sp. nov., larva, habitus. a. Male, dorsal view; b. Male, lateral view; c. Female, dorsal view; d. Female, ventral view. Scale bars: 1 mm.

**Figure 2.**
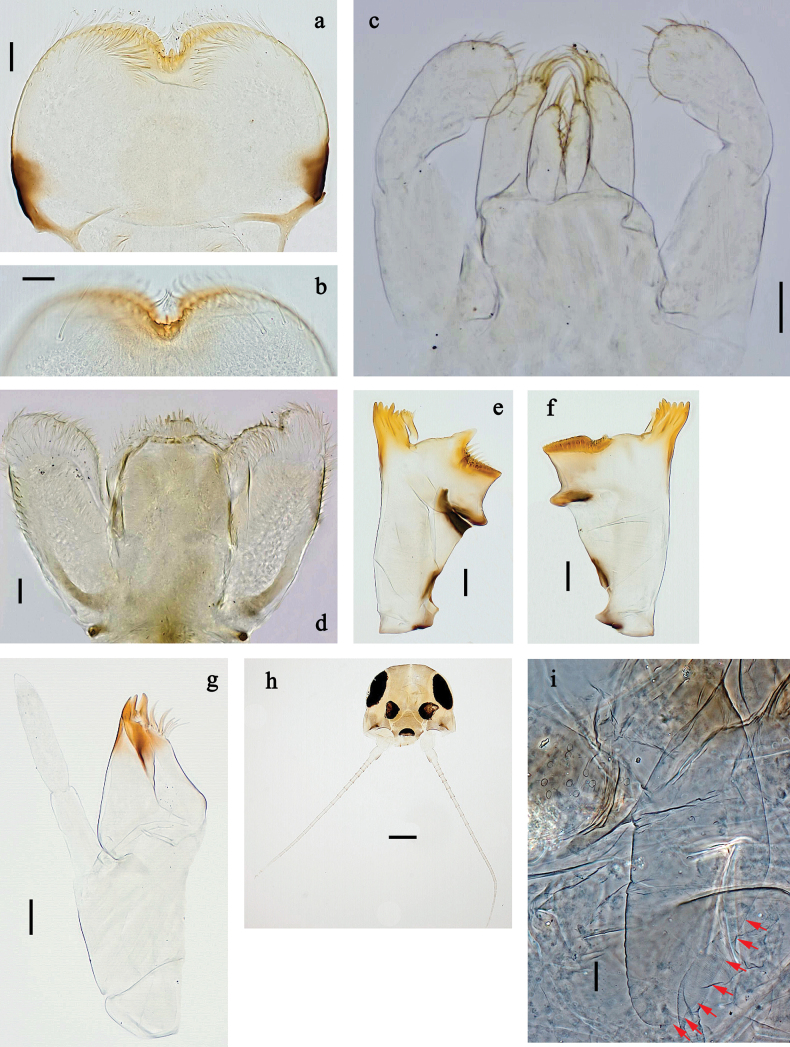
*Baetis procul* sp. nov., larva. a, b. Labrum; c. Labium; d. Hypopharynx and superlinguae; e. Left mandible; f. Right mandible; g. Maxilla; h. Head; i. Hind protopteron (arrows: costal margin). Scale bars: 100 µm (h); 20 µm (a, c–g); 10 µm (b, i).

**Figure 3.**
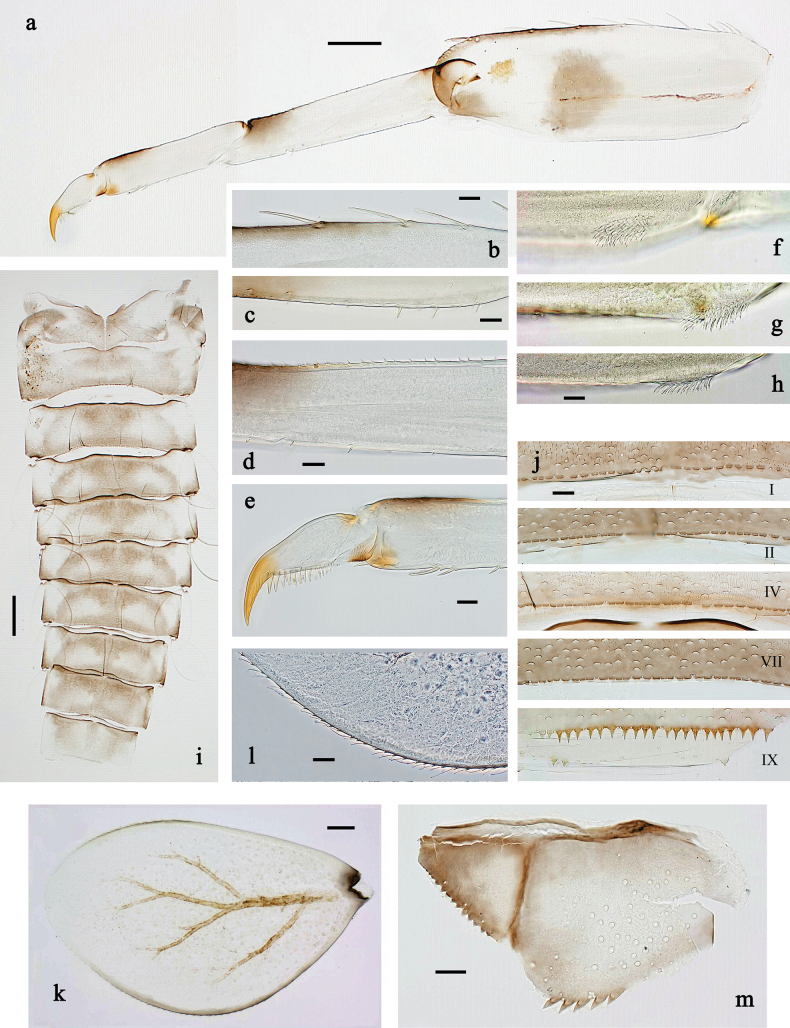
*Baetis procul* sp. nov., larva. a. Hind leg; b. Femur, outer margin; c. Femur, inner margin; d. Tibia; e. Tarsus, claw; f. Femoral patch, fore leg; g. Femoral patch, middle leg; h. Femoral patch, hind leg; i. Abdomen; j. Abdominal segments; k, l. Tergalius IV; m. Paraproct. Scale bars: 100 µm (i); 50 µm (a); 20 µm (k); 10 µm (b–h, j, l, m).

**Larva** (Figs 1–3). Body length 3.1–4.0 mm. Cerci ~½ body length; paracercus ~⅓ of body length. ***Antenna***: ~ 2× head length.

***Colouration*** (Figs 1a–d, 3a, i). Head and thorax dorsally grey with indistinct yellowish grey pattern as in Fig. 1a, c. Abdominal terga I–VIII dorsally grey-brown, with distinct sublateral, yellowish grey markings; terga IX and X yellowish grey. Fore protoptera grey. Thorax and abdomen ventrally pale yellowish grey, laterally with grey streaks. Legs off-white, femur distomedially with grey marking, tibia and tarsus distally darker. Caudalii off-white, with dark brown section in middle part, cerci with additional dark brown section subdistally.

***Labrum*** (Fig. 2a, b). Sub-rectangular, length 0.6× maximum width. Distal margin with medial emargination and small process. Dorsally with pair of submedian setae, and submarginal arc of three or four medium to long, simple setae on each side. Ventrally with marginal row of setae composed of anterolateral long, feathered setae and medial long, bifid, feathered setae.

***Right mandible*** (Fig. 2f). Incisor and kinetodontium fused. Incisor with five denticles; kinetodontium with three denticles. Prostheca robust, apically denticulate. Margin between prostheca and mola slightly convex, smooth. Tuft of setae on proximal corner of mola present.

***Left mandible*** (Fig. 2e). Incisor and kinetodontium fused. Incisor with four denticles, kinetodontium with three denticles. Prostheca robust, apicolaterally with small denticles and comb-shaped structure. Margin between prostheca and mola almost straight, with few minute denticles. Tuft of setae on proximal corner of mola absent.

Both mandibles with lateral margins almost straight.

***Hypopharynx and superlinguae*** (Fig. 2d). Lingua approx. as long as superlinguae. Lingua longer than broad; medial tuft of stout setae poorly developed, short. Superlinguae with lateral margins rounded; fine, long, simple setae along distal margin.

***Maxilla*** (Fig. 2g). Galea-lacinia ventrally with two simple, apical setae below canines. Medially with one feathered, spine-like seta and three short to long, simple setae. Maxillary palp slightly longer than length of galea-lacinia; 2-segmented; palp segment II ~1.1× as long as segment I; setae on maxillary palp fine, simple, scattered over surface of segments I and II; apex of segment II slightly pointed.

***Labium*** (Fig. 2c). Glossa basally broad, narrowing toward apex; shorter than paraglossa; inner margin with ~7 spine-like seta; apex with three long robust setae; outer margin with ~3 spine-like setae; ventral surface with fine, simple, scattered setae. Paraglossa sub-rectangular, slightly curved inward; apex rounded; with three rows of long, robust, distally pectinate setae in apical area and two short, simple setae in anteromedial area; dorsally with four long, spine-like setae near inner margin. Labial palp with segment I approx. as long as length of segments II and III combined. Segment II without developed distomedial protuberance; dorsally with two or three spine-like setae near outer margin. Segment III nearly semicircular; length ~0.8× maximal width; ventrally covered with short, spine-like, simple setae and short, fine, simple setae.

***Hind protoptera*** (Fig. 2i) present, well developed.

***Legs*** (Fig. 3a–h). Ratio of foreleg segments 1.5:1.0:0.8:0.3, middle leg 1.6:1.0:0.8:0.4, hind leg 1.5:1.0:0.7:0.3. ***Femur*.** Femur length ~3× maximum width. Outer margin with a row of 9–17 spine-like setae; length of setae ~0.3× maximum width of femur. Apex rounded, with pair of spine-like setae and short, stout, apically blunt setae. Stout, lanceolate, pointed setae scattered along inner margin; femoral patch well developed on all legs. ***Tibia*.** Outer margin with row of short, stout, pointed setae. Inner margin with spaced row of short, spine-like setae, on apex tuft of fine, simple setae. Patella-tibial suture present on basal ¾. ***Tarsus*.** Outer margin with spaced row of short, stout, pointed setae. Inner margin with row of curved, spine-like setae increasing in length distally. ***Claw*** with one row of 13–15 denticles; distally pointed.

***Abdominal terga*** (Fig. 3i, j). Surface with irregular rows of U-shaped scale bases. Posterior margin of terga: I–VIII with small, short, rounded, transparent spines; IX with longer, acute, triangular, spines.

***Abdominal sterna*** (Fig. 3j). Posterior margin of sterna: I–VIII smooth, without spines; IX laterally with small, triangular spines.

***Tergalii*** (Fig. 3k, l). Present on segments I–VII. Skew oval in shape, margin with small denticles intercalating fine, simple setae. Tergalius I as long as length of segment II; tergalius IV as long as segments V and VI combined; tergalius VII slightly longer than segment VIII.

***Paraproct*** (Fig. 3m). Distally not expanded, with five or six stout, marginal spines. Cercotractor with numerous small, marginal spines.

**Imago. Male.** Unknown.

##### Diagnosis.

**Imago, female.** Following characters differentiate *Baetis procul* sp. nov. from *Baetis taiwanensis* Müller-Liebenau, 1985 (Fujitani et al. 2004: figs 3, 6): hindwing narrower, with anal margin concave; costal process more developed.

##### Description.

**Figure 4.**
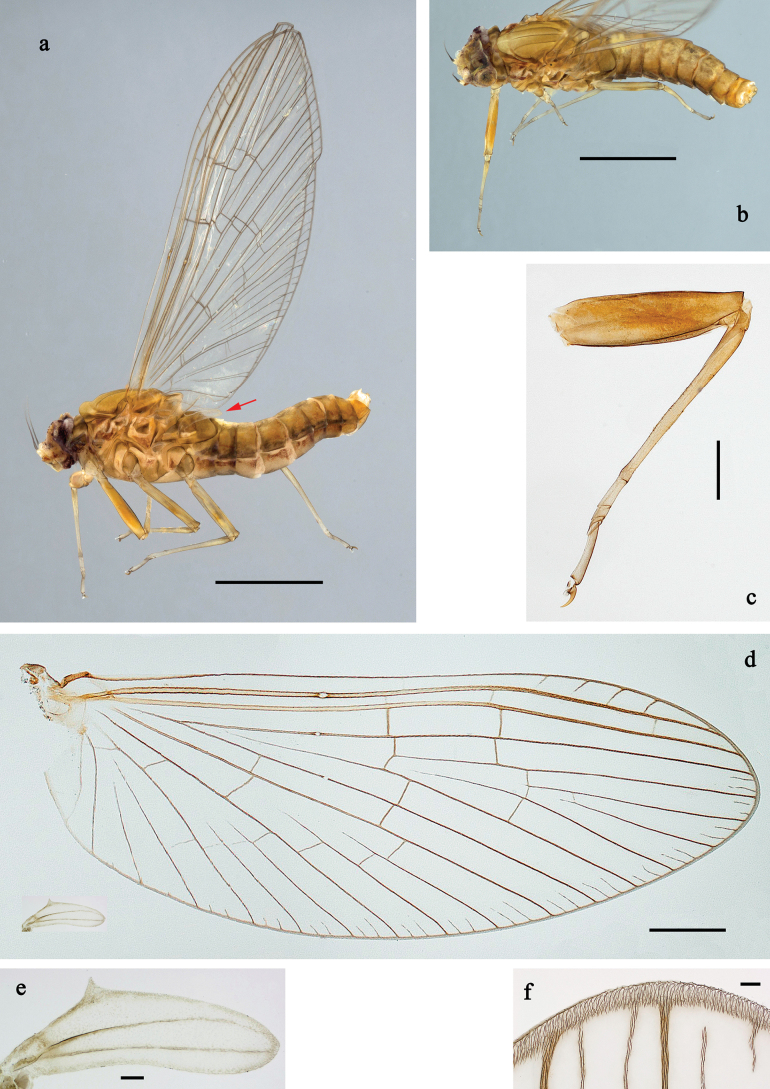
*Baetis procul* sp. nov., female imago. a. Habitus, lateral view (arrow: hind wing); b. Habitus, dorsal view; c. Fore leg; d. Wings; e. Hind wing; f. Apex of fore wing. Scale bars: 1 mm (a, b); 200 µm (d); 100 µm (c); 20 µm (e); 10 µm (f).

**Imago, female** (Fig. 4a–f). Body length ~3.2 mm, fore wing length ~3.9 mm, hind wing length ~0.5 mm. Head and antennae dark brown. Thorax and abdomen grey-brown. Fore wing with membrane colourless, base of wings slightly brownish; veins brown, covered with very fine setae; pterostigma with four oblique crossveins, mostly nearly complete. Hind wing very small, narrow, anal margin concave; with two nearly parallel, longitudinal veins; costal margin with distinct process in basal ½; veins grey-brown, covered with very fine setae similar to fore wing. Legs grey-brown; femur anteriorly with diffuse reddish brown colour, more developed on foreleg.

##### Biological aspects.

Living in the run of medium fast flowing lowland rivers in algae on stones (Figs 36c, 37b). Female imagos are laying their eggs under water, probably in algae. When caught by kick-sampling, larvae are always swimming very fast and straight to the next algae or debris to cling on.

##### Etymology.

The name *procul*, meaning “far away” or “at a distance” in Latin, refers to the large geographic distance of this new species to other known *Baetis* species (see discussion).

##### Distribution.

Fiji, Viti Levu (Fig. 39a).

### ﻿Genus *Papuanatula* Lugo-Ortiz & McCafferty, 1999

Lugo-Ortiz and McCafferty 1999

Kaltenbach, Kluge and Gattolliat 2025a: redescription of genus *Papuanatula*; subgenera *Papuanatula* s. str. and *Papuafiliola* Kaltenbach, Kluge & Gattolliat.

#### ﻿Subgenus Fijifiliola subgen. nov.

Taxon classificationAnimaliaEphemeropteraBaetidae

894E9BAB-13B6-544F-9EA6-721A6D829125

https://zoobank.org/ACC67C58-CE8B-4897-A87D-148BAA8A881D

##### Type species.

Papuanatula (Fijifiliola) aji sp. nov.

##### Diagnosis

**Figure 5.**
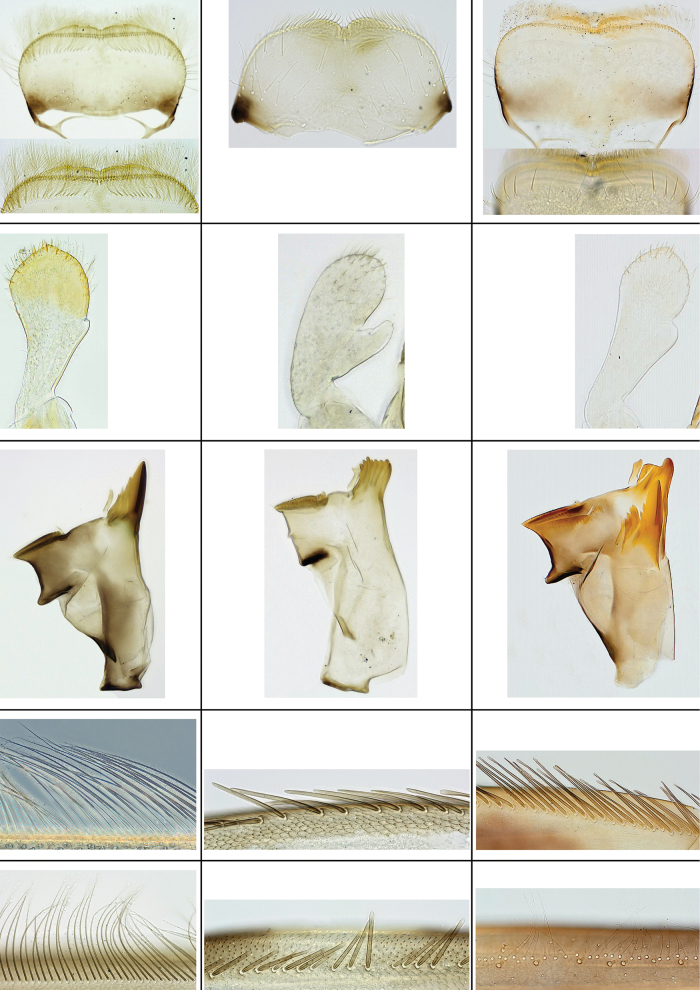
*Papuanatula*, comparison of main characters between subgenera: left column: *Papuanatula* s. str.; middle column: *Papuafiliola*; right column: Fijifiliola subgen. nov.; row one (top): labrum, shape, and dorsal submarginal arc of setae; row two: labial palp segment II/III; row three: right mandible; row four: femur, outer margin row of setae; row five (bottom): tibia, anterior surface, row of setae.

**(larval characters)** (Table 3, Fig. 5).

**Table 3.** Comparison of main larval characters of *Papuanatula* subgenera.

Characters	Papuanatula s. str.	Papuafiliola	Fijifiliola subgen. nov.
Antenna: distal flagellomeres	Asymmetrical with brown dots	Symmetrical without brown dots	Asymmetrical with brown dots
Labrum: shape	widest medially	widest at base	widest medially
Labrum: dorsal submarginal arc	feathered setae	few simple setae	simple setae
Mandibles: incisor	blade-like	not blade-like	blade-like
Labial Palp: segment II	no real protuberance	narrow, thumb-like protuberance	short, rounded protuberance
Femur: setae outer margin	long, pointed, ciliate	long, flattened, parallel-sided, apex blunt, not ciliate	long, flattened, apex usually blunt, not ciliate
Tibia: anterior row of setae	long, pointed, ciliate	similar to femur, not ciliate	long, fine, simple; not ciliate; 1–2 rows short, stout setae
Tarsus: distal seta inner margin (length vs. other setae)	much longer	not much longer	much longer
Paraproct: distal margin	usually expanded	not expanded	usually expanded
Untypical morphology	*P. normungulata* (Sulawesi)	none	*P. crussetae* sp. nov. *P. tenebris* sp. nov.
Number of species	29	4	10
Distribution	New Guinea, Sulawesi	New Guinea	Fiji Islands

Distal part of antenna asymmetric, each flagellomere with anterior side more convex than posterior side, with brown hypodermal spot near apex of anterior side (Fig. 8j).

***Labrum*** (Fig. 7a, b) widest in middle area, laterally rounded; dorsally with submarginal row of long, simple setae.

***Hypopharynx and superlinguae*** (Fig. 7d). Apex of lingua usually with a well-developed, bifid bunch of stout setae. Distolateral margin of superlinguae with medium, fine setae; lateral margins almost straight.

Both mandibles long, with incisor strongly elongated (blade-like); full-length incisors present only at the beginning of each instar, usually worn at the end of instar (Fig. 7e, f).

***Right mandible*** (Fig. 7f). Kinetodontium deeply separated from incisor; incisor with three denticles; kinetodontium terminating with three or four denticles, with distal denticle longest. Mola with setae on proximal corner.

***Left mandible*** (Fig. 7e). Incisor and kinetodontium fused at most of its length; incisor with three denticles; kinetodontium terminating with three or four denticles, with distal denticle longest. Mola with setae on proximal corner.

***Maxilla*** (Fig. 7g). With one or two simple setae under canines. Medially with one spine-like, feathered seta and usually three short to long simple setae. Maxillary palp apically constricted.

***Labium*** (Fig. 7c). Glossae longer than half of paraglossae; with several long setae at and near apex. Paraglossae with one medium simple seta in medioproximal area. Labial palp with poorly developed, rounded, distomedial projection on segment II.

***Thorax and abdomen*** dorsally and ventrally usually without protuberances. The only known exception is a minute spine posteromedially on fore protoptera of mature larvae of *P. aji* sp. nov. Posterior margin of abdominal sternites smooth, without spines.

***Legs*** (Fig. 8a–d). Outer side of femur usually with single, regular row of long, slender, flattened setae with blunt apex; additionally in distal ½ row of medium to long, fine, simple setae; apex with short, stout, blunt setae; short to medium, spine-like setae along inner margin. Anterior side of tibia with regular row of long, fine, simple setae; parallel one or two rows of short, stout setae with blunt apices. Tarsus with regular row of long, fine, simple setae along outer margin; inner margin subdistally usually with one much longer, thinner, pointed seta. Claw with single row of denticles and usually one posterior seta.

***Paraproct*** (Fig. 8h). Posterior margin usually with prolongation and with split denticles. Cercotractor usually with small denticles.

***Pose of subimaginal gonostyli under larval cuticle*** (Fig. 7j). Subimaginal gonostyli developing under cuticle of last instar male larvae folded in “*Labiobaetis*-type” (Kluge 2004: fig. 29I): 1^st^ segment directed laterally, 2^nd^ and 3^rd^ segments directed medially, whereby second segment is closest to posterior margin of the ninth segment.

##### Etymology.

The new genus-group name Fijifiliola subgen. nov. is formed from the nouns Fiji and filiola (meaning “little daughter” in Latin), and can be understood as “little daughter of Fiji”. Gender is feminine.

### ﻿Species included in Fijifiliola subgen. nov.

*Papuanatula aji* sp. nov.

*Papuanatula aurantica* sp. nov.

*Papuanatula bula* sp. nov.

*Papuanatula claudia* sp. nov.

*Papuanatula crussetae* sp. nov.

*Papuanatula flowersi* sp. nov.

*Papuanatula gattolliati* sp. nov.

*Papuanatula grisea* sp. nov.

*Papuanatula sekawa* sp. nov.

*Papuanatula tenebris* sp. nov.

#### ﻿ Papuanatula (Fijifiliola) aji

Taxon classificationAnimaliaEphemeropteraBaetidae

sp. nov.

FA2598CA-45AE-547C-A1C4-2BCE52C22212

https://zoobank.org/78922C63-153A-4499-A1B9-8B3C59E0B714

Figs 6, 7, 8

##### Material examined.

***Holotype*.** FIJI • larva; Viti Levu, Ba Prov., Ba Riv., Qerelevu, near Outback Hotel; 17°37'35"S, 177°45'04"E; 20 m; 21.x.2024; leg. T. Kaltenbach; on slide; GBIFCH00975845; MZL. ***Paratypes*.** • 25 larvae; same data as holotype; 3 on slides; GBIFCH00975846, GBIFCH00975847, GBIFCH01581944; 22 in alcohol; GBIFCH01581941, GBIFCH01581942, GBIFCH01581943; MZL • 32 larvae; Viti Levu, Ba Prov., Ba Riv., Qerelevu, near Outback Hotel; 17°37'35"S, 177°45'04"E; 20 m; 22.x.2024; leg. T. Kaltenbach; in alcohol; GBIFCH01581960, GBIFCH01581961, GBIFCH01581962, GBIFCH01581963, GBIFCH01582031; MZL • 22 larvae; Viti Levu, Ba Prov., creek, near Outback Hotel; 17°38'12"S, 177°45'29"E; 157 m; 25.x.2024; leg. T. Kaltenbach; in alcohol; GBIFCH01581912, GBIFCH01581916, GBIFCH01581917, GBIFCH01581918; MZL • 13 larvae; Vanua Levu, Cacaudrove Prov., tributary to Sekawa Riv., near Nakawaga; 16°39'55"S, 179°20'03"E; 32 m; 29.x.2024; leg. T. Kaltenbach; in alcohol; GBIFCH01581954, GBIFCH01582016, GBIFCH01582017; MZL • 4 larvae; Vanua Levu, Cacaudrove Prov., Sekawa Riv., many filamentous algae; near Nakawaga; 16°39'54"S, 179°20'01"E; 32 m; 29.x.2024; leg. T. Kaltenbach; in alcohol; GBIFCH01581909, GBIFCH01581910; MZL • 14 larvae; Vanua Levu, Cacaudrove Prov., Seaqaqa Riv., bridge near Saivou village; 16°36'25"S, 179°08'46"E; 80 m; 05.xi.2024; leg. T. Kaltenbach; in alcohol; GBIFCH01581900, GBIFCH01581984, GBIFCH01581985, GBIFCH01581986, GBIFCH01581987; MZL • 42 larvae; Viti Levu, Ba Prov., Sabeto Riv., near Sabeto; 17°43'09"S, 177°31'21"E; 30 m; 17.xi.2024; leg. T. Kaltenbach; in alcohol; GBIFCH01581971, GBIFCH01581973, GBIFCH01581974, GBIFCH01581975, GBIFCH01581976, GBIFCH01581977, GBIFCH01581978, GBIFCH01582027, GBIFCH01582028; MZL • 1 larva; Viti Levu, Ba Prov., near Abaca village, Savuione Riv., close below Savuione waterfall; 17°40'26"S, 177°33'06"E; 690 m; 24.x.2024; leg. T. Kaltenbach; in alcohol; GBIFCH01581939; MZL • 16 larvae; Viti Levu, Ba Prov., near Abaca village, Savuione Riv.; 17°40'07"S, 177°32'31"E; 510 m; 24.x.2024; leg. T. Kaltenbach; 1 on slide; GBIFCH01581928; 15 in alcohol; GBIFCH01581924, GBIFCH01581926, GBIFCH01581927, GBIFCH01582018, GBIFCH01582021, GBIFCH01582022, GBIFCH01582023, GBIFCH01582024, GBIFCH01582025; MZL.

##### Diagnosis.

**Figure 6.**
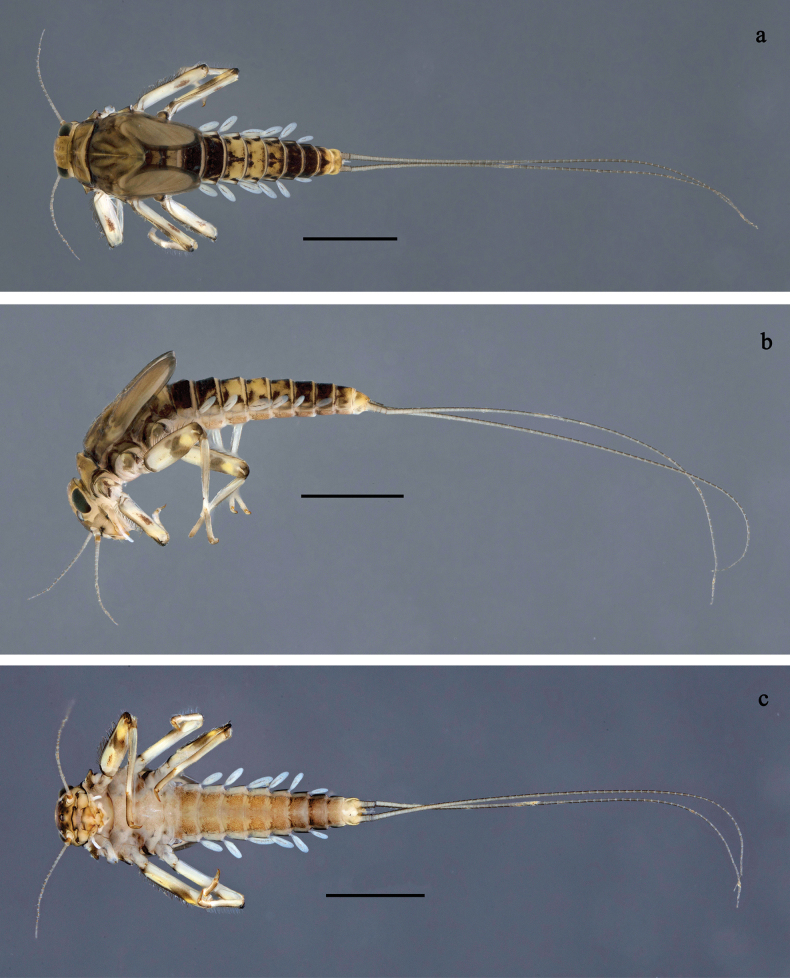
Papuanatula (Fijifiliola) aji sp. nov., larva, habitus. a. dorsal view; b. Lateral view; c. Ventral view. Scale bars: 1 mm.

**Figure 7.**
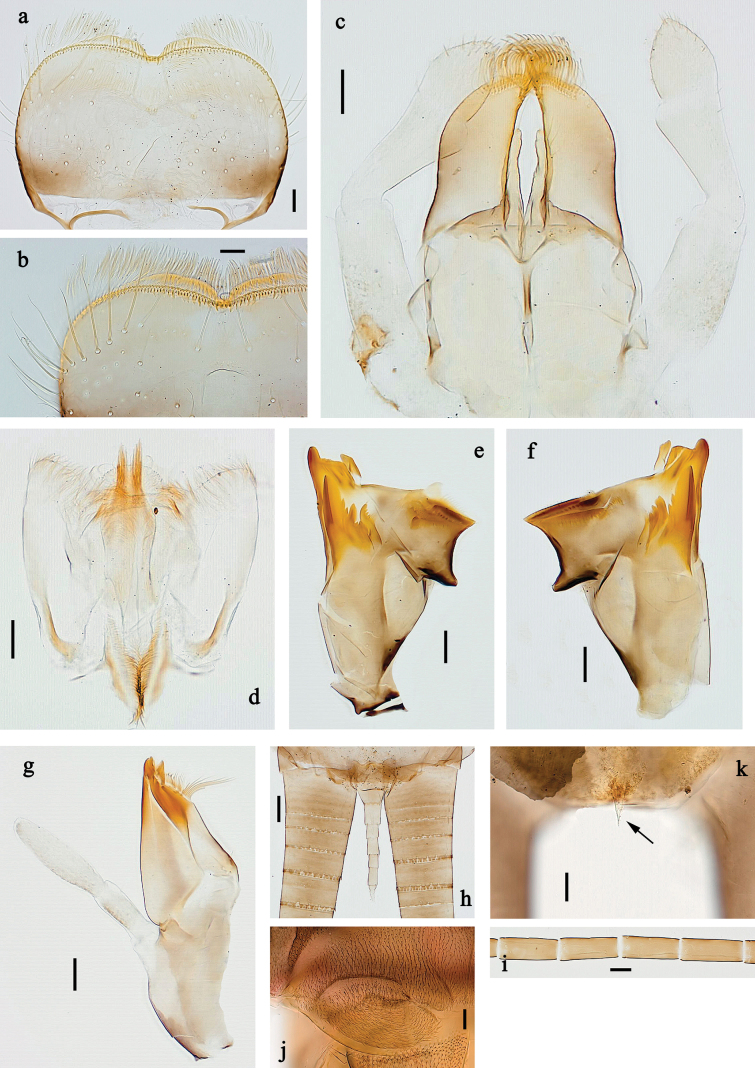
Papuanatula (Fijifiliola) aji sp. nov., larva. a, b. Labrum; c. Labium; d. Hypopharynx and superlinguae; e. Left mandible; f. Right mandible; g. Maxilla; h. Paracercus; i. Cercus; j. Male mature larva, developing subimaginal gonostylus; k. Fore protoptera, protuberance. Scale bars: 20 µm (a, c–h). 10 µm (b, i–k).

**Larva**. Following combination of characters distinguishes *P. aji* sp. nov. from other species of Fijifiliola subgen. nov.: fore protoptera posteromedially with minute spine (Fig. 7k); labrum without small process in distomedial emargination; labial palp segment III conical, pointed; abdominal tergum I grey, II, III, VII, and VIII blackish, IV–VI, IX, and X yellowish grey with blackish markings as in Fig. 6a, b; femur anteriorly with dark brown hypodermal streak in mediobasal blank (usually developed on fore femur, less on middle femur and absent on hind femur); femur anteriorly with large grey-brown marking in distomedial area, yellowish in subdistal area; paracercus with eight segments.

##### Description.

**Figure 8.**
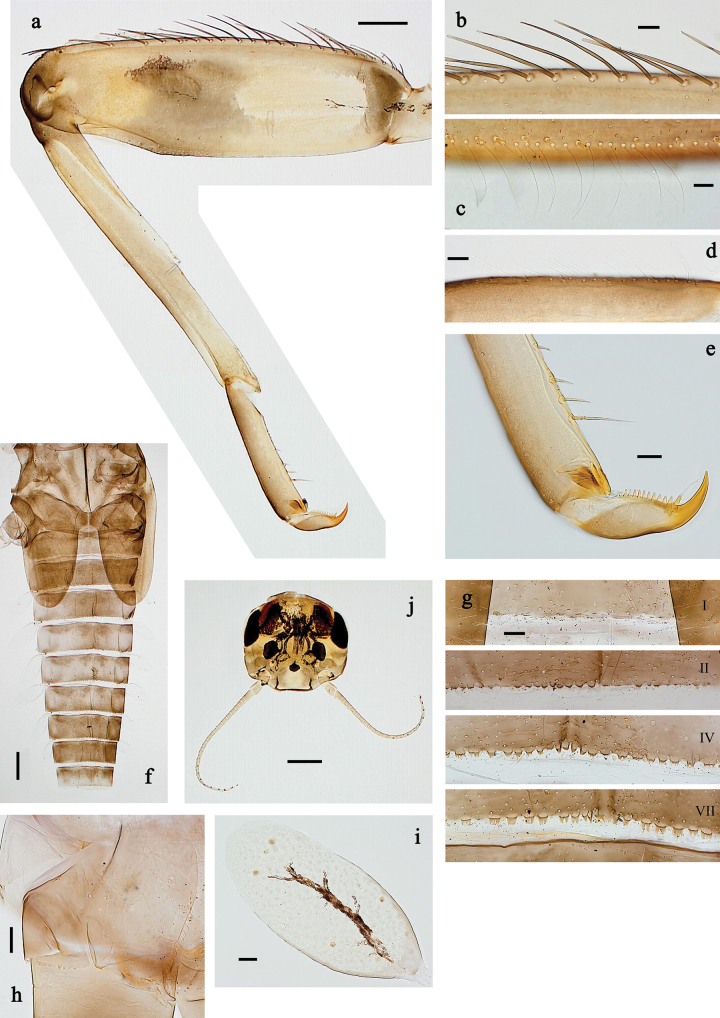
Papuanatula (Fijifiliola) aji sp. nov., larva. a. Hind leg; b. Femur, outer margin; c. Tibia; d. Tarsus, outer margin; e. Tarsus, claw; f. Abdomen; g. Abdominal terga; h. Paraproct; i. Tergalius IV; j. Head. Scale bars: 100 µm (f, j). 50 µm (a); 10 µm (b–e, g–i).

**Larva** (Figs 6–8). Body length 2.8–3.5 mm, cerci ~1.5× body length.

***Colouration*** (Figs 6a–c, 8f). Head and thorax dorsally dark grey with indistinct pattern, fore protoptera grey with dark grey margins. Abdominal tergum I grey, II, III, VII, and VIII blackish, IV–VI, IX, and X yellowish grey with blackish markings as in Fig. 6a, b. Thorax and abdomen ventrally grey to reddish grey, sternites VII and VIII darker. Legs grey; femur on anterior side apically, distomedially, and basally dark grey, basal area with large blank and hypodermal streak inside (developed on fore femur, poorly developed on middle femur, absent on hind femur), subdistal area yellowish; femur of all legs on posterior side with brown streak in distomedial area along outer margin. Caudalii grey.

***Head*. *Antenna*** (Fig. 8j). Length ~1.5× head length. As typical for subgenus.

***Labrum*** (Fig. 7a, b). Length 0.6× maximum width. Distomedial emargination without protuberance. Otherwise, as typical for subgenus.

***Right mandible*** (Fig. 7f). Margin between prostheca and mola straight, smooth. Otherwise, as typical for subgenus.

***Left mandible*** (Fig. 7e). Margin between prostheca and mola straight, smooth. Otherwise, as typical for subgenus.

***Hypopharynx and superlinguae*** (Fig. 7d). As typical for subgenus.

***Maxilla*** (Fig. 7g). Maxillary palp approx. as long as galea-lacinia; palp segment II ~1.4× longer than segment I. Otherwise, as typical for subgenus.

***Labium*** (Fig. 7c). Paraglossa with two spine-like setae on inner, distolateral margin. Labial palp with segment I subequal in length to segments II and III combined. Segment II dorsally with row of five spine-like setae near outer, distolateral margin. Segment III conical, pointed, 0.6× length of segment II. Otherwise, as typical for subgenus.

***Legs*** (Fig. 8a–e). Ratio of leg segments: fore leg 1.0:1.0:0.4:0.2, middle leg 1.1:1.0:0.4:0.2 and hind leg 1.1:1.0:0.4:0.2. ***Femur*.** Length ~3 × maximum width. ***Tibia*.** With one row of short, blunt setae parallel to row of long, fine, simple setae. ***Claw*** with one row of 11–14 denticles; one posterior seta. Otherwise, as typical for subgenus.

***Abdomen*. *Terga*** (Fig. 8f, g). Posterior margin of terga: I smooth, without denticles; II–IX with short, triangular, mostly split denticles.

***Tergalii*** (Fig. 8i). Elongate oval, rather narrow; tracheation mainly limited to main trunk; margin smooth, with short, fine, simple setae.

***Paraproct*** (Fig. 8h). Posterior margin with prolongation, with split denticles in distal part.

***Caudalii*** (Fig. 7h, i). Cerci without swimming setae. Paracercus with 7–10 segments.

***Pose of subimaginal gonostyli under larval cuticle*** (Fig. 7j). As typical for subgenus. Segment III conical.

**Subimago.** Unknown.

**Imago.** Unknown.

**Egg.** Unknown.

##### Biological aspects.

By far the most widespread and most frequent *Papuanatula* species of Fiji. Larvae usually living in run or riffles of medium fast flowing lowland rivers, sometimes in fast flowing, turbulent mountain rivers (Figs 36b–e, 37a, b, 38a–c). Occurs in more pristine rivers as well as in rivers influenced by human activities.

##### Etymology.

The species is dedicated to AJ, the owner of the Outback Hotel, where the type locality is located. He and his family provided great support during our stay and on collection excursions.

##### Distribution.

Fiji: Viti Levu, Vanua Levu (Fig. 39b).

#### ﻿ Papuanatula (Fijifiliola) aurantica

Taxon classificationAnimaliaEphemeropteraBaetidae

sp. nov.

197B2BA0-998D-5793-B665-5DEF9025D817

https://zoobank.org/36040D0D-8862-4C6F-8DAF-41FB6F1B0C24

Figs 9, 10, 11

##### Material examined.

***Holotype*.** FIJI • larva; Viti Levu, Serua Prov., Biausevu Riv., near Biausevu; 18°10'57"S, 177°44'13"E; 70 m; 10.xi.2024; leg. T. Kaltenbach; on slides; GBIFCH01221830, GBIFCH01582013; MZL.

##### Diagnosis.

**Larva**. Following combination of characters distinguishes *P. aurantica* sp. nov. from other species of Fijifiliola subgen. nov.: Head, thorax, and abdomen predominantly orange to orange-brown; legs in basal part with orange-brown, hypodermal marking in oval blank; medially with broad, grey band; with orange streak along outer margin and orange in subdistal part; labial palp segment III slightly pentagonal; paracercus with 14 segments.

##### Description.

**Figure 9.**
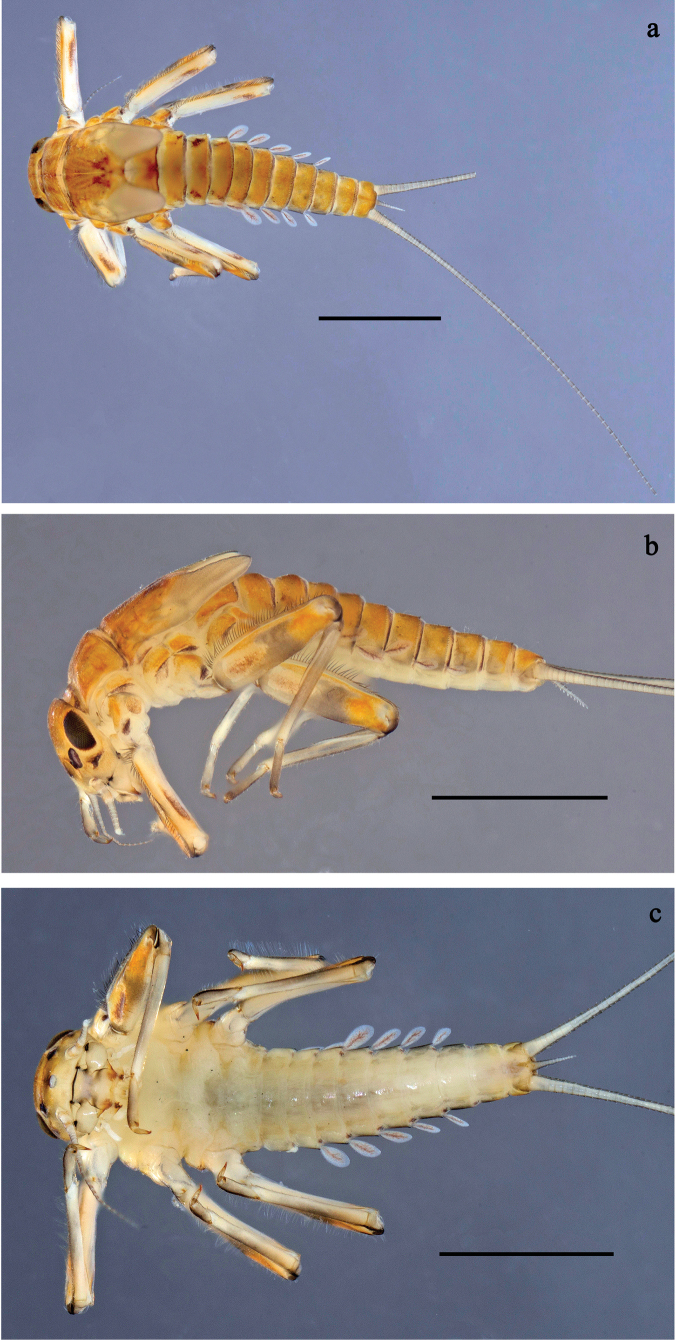
Papuanatula (Fijifiliola) aurantica sp. nov., larva, habitus. a. Dorsal view; b. Lateral view; c. Ventral view. Scale bars: 1 mm.

**Figure 10.**
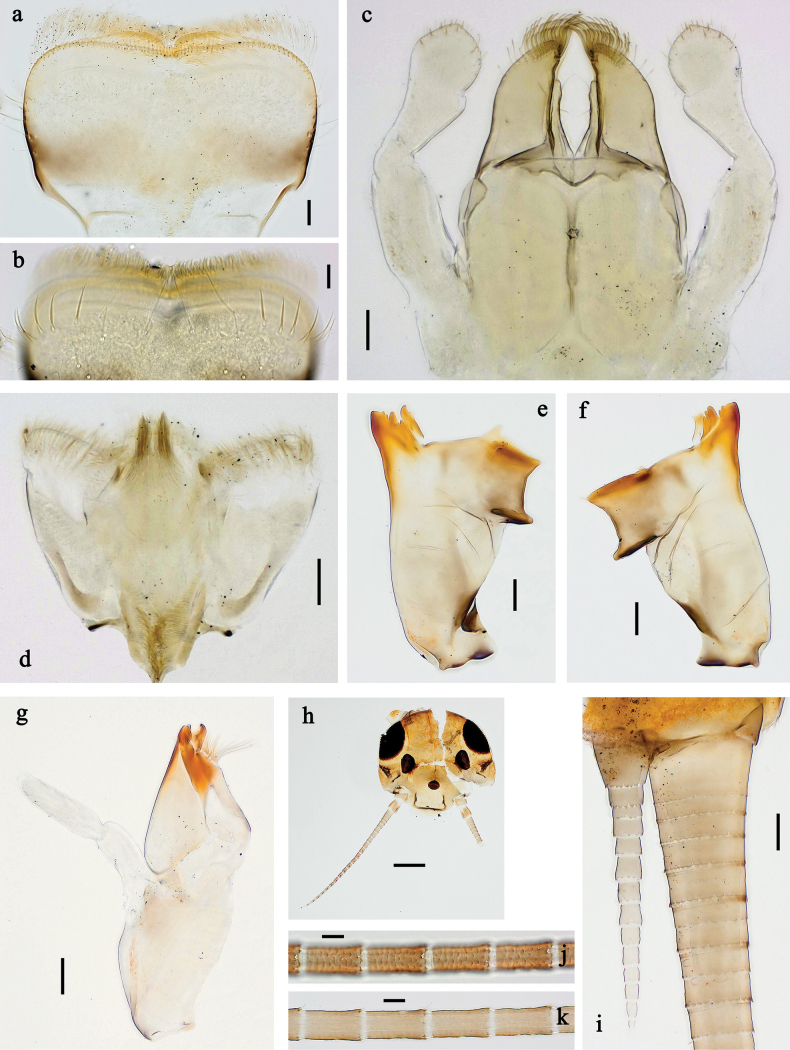
Papuanatula (Fijifiliola) aurantica sp. nov., larva. a, b. Labrum; c. Labium; d. Hypopharynx and superlinguae; e. Left mandible; f. Right mandible; g. Maxilla; h. Head; i. Paracercus; j, k. Cerci. Scale bars: 100 µm (h); 20 µm (a, c–g, i); 10 µm (b, j, k).

**Figure 11.**
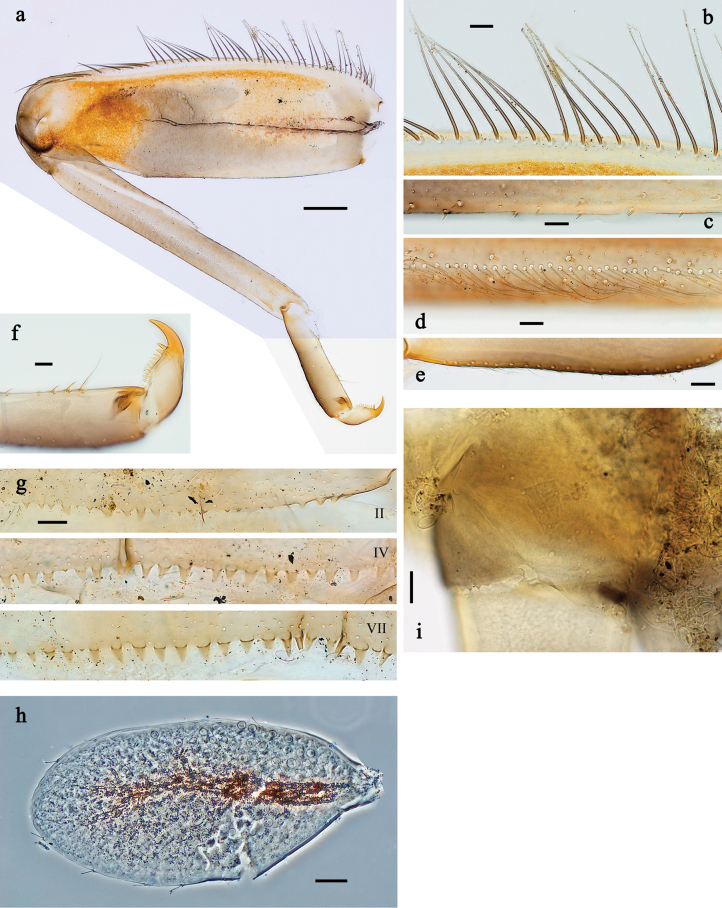
Papuanatula (Fijifiliola) aurantica sp. nov., larva. a. Hind leg; b. Femur, outer margin; c. Femur, inner margin; d. Tibia; e. Tarsus, outer margin; f. Tarsus, claw; g. Abdominal terga; h. Tergalius III; i. Paraproct. Scale bars: 50 µm (a); 10 µm (b–i).

**Larva** (Figs 9–11). Body length ~3 mm, cerci ~1.3× body length.

***Colouration*** (Fig. 9a–c). Head, thorax and abdomen dorsally predominantly orange to orange-brown, thorax with indistinct pattern, fore protoptera grey; abdominal terga I–III laterally with oblique, brown streaks. Head, thorax, and abdomen ventrally beige. Legs in basal part with orange-brown, hypodermal marking in oval blank; medially with broad, grey band; with orange streak along outer margin and orange in subdistal part; distally dark grey; femur of all legs on posterior side with brown streak in distomedial area along outer margin. Caudalii grey.

***Head*. *Antenna*** (Fig. 10h). Length ~1.5× head length. As typical for subgenus.

***Labrum*** (Fig. 10a, b). Length 0.6× maximum width. Otherwise, as typical for subgenus.

***Right mandible*** (Fig. 10f). Margin between prostheca and mola straight, with few minute denticles. Otherwise, as typical for subgenus.

***Left mandible*** (Fig. 10e). Margin between prostheca and mola straight, with few denticles toward subtriangular process. Otherwise, as typical for subgenus.

***Hypopharynx and superlinguae*** (Fig. 10d). As typical for subgenus.

***Maxilla*** (Fig. 10g). Maxillary palp approx. as long as galea-lacinia; palp segment II ~1.2× longer than segment I. Otherwise, as typical for subgenus.

***Labium*** (Fig. 10c). Paraglossa with two spine-like setae on inner, distolateral margin. Labial palp with segment I ~1.2× length of segments II and III combined. Segment II dorsally with row of four or five spine-like setae near outer, distolateral margin. Segment III slightly pentagonal, 0.7× length of segment II. Otherwise, as typical for subgenus.

***Legs*** (Fig. 11a–f). Ratio of leg segments: fore leg 0.9:1.0:0.3:0.2, middle leg 1.1:1.0:0.4:0.2 and hind leg 1.1:1.0:0.3:0.2. ***Femur***. Length ~3 × maximum width. Setae on outer margin long, flattened, pointed. ***Tibia*.** With one row of short, blunt setae on each side of row of long, fine, simple setae. ***Claw*** with one row of ten or eleven denticles; one posterior seta. Otherwise, as typical for subgenus.

***Abdomen*. *Terga*** (Fig. 11g). Posterior margin of terga: I smooth, without denticles; II–IX with triangular denticles.

***Tergalii*** (Fig. 11h). Elongate oval; tracheation mainly limited to main trunk, brown; scattered orange pigmentation outside tracheae; margin smooth, with short, fine, simple setae.

***Paraproct*** (Fig. 11i). Posterior margin with prolongation, with denticles.

***Caudalii*** (Fig. 10i, k). Cerci without swimming setae. Paracercus with 14 segments.

***Pose of subimaginal gonostyli under larval cuticle*.** Unknown.

**Subimago.** Unknown.

**Imago.** Unknown.

**Egg.** Unknown.

##### Biological aspects.

Local species, only known from Biausevu River. Larvae living in run of medium fast flowing rivers (Fig. 37c), in natural forest with pristine water.

##### Etymology.

The species name refers to its predominantly orange colour, *aurantica* meaning orange in Latin.

##### Distribution.

Fiji: Viti Levu (Fig. 39c).

#### ﻿ Papuanatula (Fijifiliola) bula

Taxon classificationAnimaliaEphemeropteraBaetidae

sp. nov.

E884A3E2-9D23-51F9-8303-9F86830F8025

https://zoobank.org/EFE10168-E817-4880-A720-7C36A6FEF70E

Figs 12, 13, 14

##### Material examined.

***Holotype*.** FIJI • larva; Viti Levu, Rewa Prov., Colo-I-Suva Forest Park, creek near Coloisuva; 18°03'35"S, 178°28'11"E; 160 m; 12.xi.2024; leg. T. Kaltenbach; on slide; GBIFCH01581921; MZL. ***Paratypes*.** • 5 larvae; same data as holotype; 1 on slide; GBIFCH01581920; 4 in alcohol; GBIFCH01581919, GBIFCH01581955, GBIFCH01581965; MZL • 6 larvae; Viti Levu, Serua Prov., Biausevu Riv., near Biausevu; 18°10'57"S, 177°44'13"E; 70 m; 10.xi.2024; leg. T. Kaltenbach; 2 on slides; GBIFCH01581982, GBIFCH01221827; 4 in alcohol; GBIFCH01581979, GBIFCH01581980, GBIFCH01581981, GBIFCH01582014; MZL • 1 larva; Viti Levu, Ba Prov., creek, near Outback Hotel; 17°38'12"S, 177°45'29"E; 157 m; 25.x.2024; leg. T. Kaltenbach; in alcohol; GBIFCH01581913; MZL • 5 larvae; Viti Levu, Ba Prov., creek, near Outback Hotel; 17°38'22"S, 177°45'30"E; 220 m; 25.x.2024; leg. T. Kaltenbach; in alcohol; GBIFCH01581915, GBIFCH01581914; MZL.

##### Diagnosis.

**Larva**. Following combination of characters distinguishes *P. bula* sp. nov. from other species of Fijifiliola subgen. nov.: femur basally with narrow dark brown to red-brown, longitudinal streak in large blank; abdominal terga II, VII, VIII dark brown, I, III–V, IX, X brighter, washy grey-brown; labial palp segment III slightly pentagonal; paracercus with 13–15 segments; cerci in middle part with one medium swimming seta each 2^nd^ segment (Fig. 13j).

##### Description.

**Figure 12.**
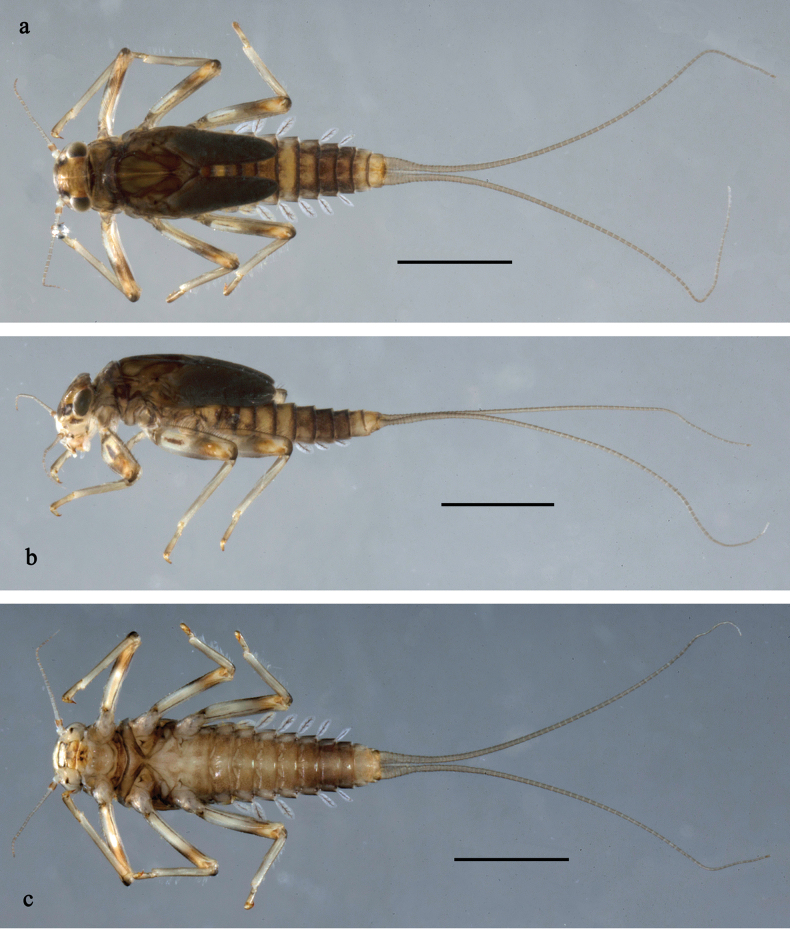
Papuanatula (Fijifiliola) bula sp. nov., larva, habitus. a. Dorsal view; b. Lateral view; c. Ventral view. Scale bars: 1 mm.

**Figure 13.**
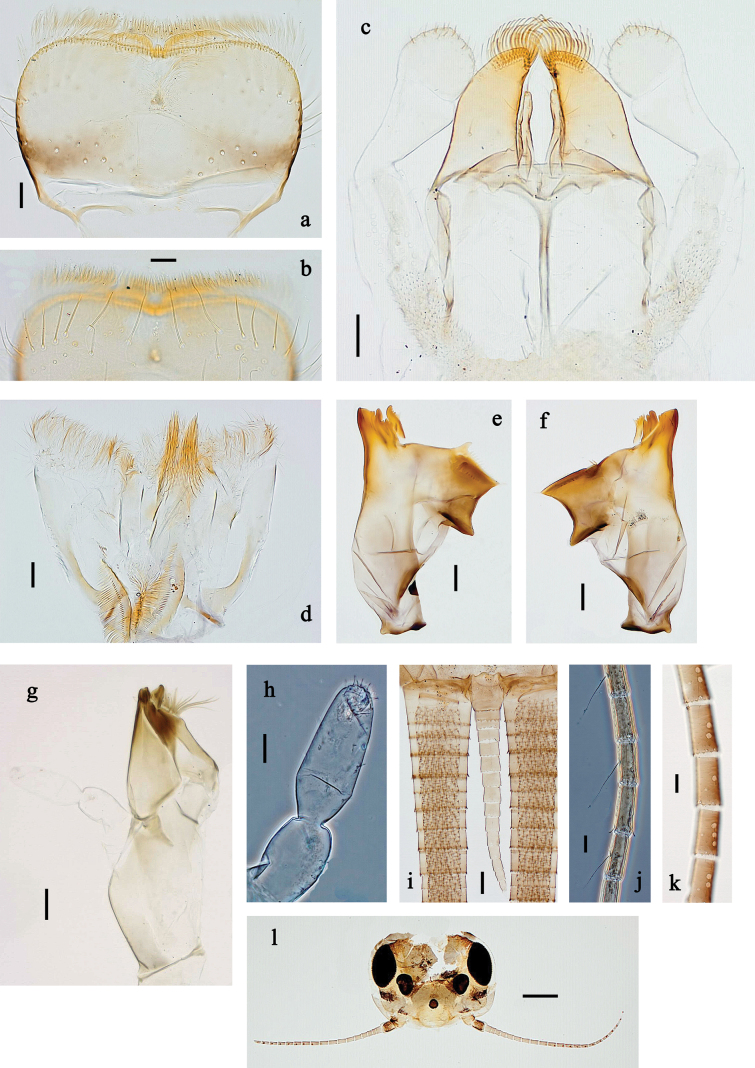
Papuanatula (Fijifiliola) bula sp. nov., larva. a, b. Labrum; c. Labium; d. Hypopharynx and superlinguae; e. Left mandible; f. Right mandible; g. Maxilla; h. Maxillary palp; i. Paracercus; j, k. Cerci; l. Head. Scale bars: 100 µm (l); 20 µm (a, c–g, i); 10 µm (b, h, j, k).

**Figure 14.**
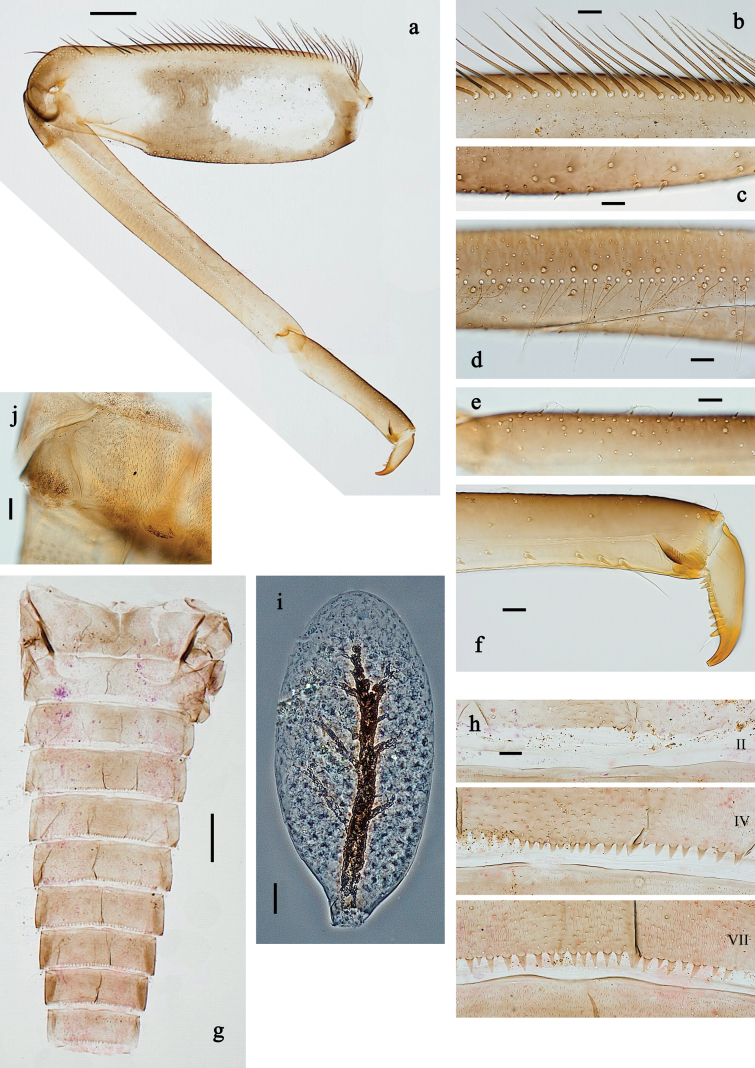
Papuanatula (Fijifiliola) bula sp. nov., larva. a. Fore leg; b. Femur, outer margin; c. Femur, inner margin; d. Tibia; e. Tarsus, outer margin; f. Tarsus, claw; g. Abdomen; h. Abdominal terga; i. Tergalius IV; j. Paraproct. Scale bars: 100 µm (g); 50 µm (a); 10 µm (b–f, h–j).

**Larva** (Figs 12–14). Body length 2.9–4.5 mm, cerci ~1.4× body length.

***Colouration*** (Figs 12a–c, 13l). Head dorsally brown, with dark brown markings; thorax dorsally dark brown; abdominal terga II, VII, VIII dark brown, I, III–V, IX, X brighter, washy grey-brown. Legs grey-brown; femur on anterior side in basal part with narrow dark brown to red-brown, hypodermal, longitudinal streak in large blank; medially with indistinct, broad, dark grey band; in distal area reddish brown; femur of all legs on posterior side reddish brown in distal ⅓, with one or two parallel, dark brown, hypodermal, longitudinal streaks. Head, thorax and abdomen ventrally grey to dark grey, sternites VI–VIII darker. Caudalii grey.

***Head*. *Antenna*** (Fig. 13l). Length ~1.5× head length. As typical for subgenus.

***Labrum*** (Fig. 13a, b). Length 0.6× maximum width. Otherwise, as typical for subgenus.

***Right mandible*** (Fig. 13f). Margin between prostheca and mola slightly convex, smooth. Otherwise, as typical for subgenus.

***Left mandible*** (Fig. 13e). Margin between prostheca and mola straight, with few denticles toward subtriangular process. Otherwise, as typical for subgenus.

***Hypopharynx and superlinguae*** (Fig. 13d). As typical for subgenus.

***Maxilla*** (Fig. 13g, h). Maxillary palp approx. as long as galea-lacinia; palp segment II ~1.2× longer than segment I. Otherwise, as typical for subgenus.

***Labium*** (Fig. 13c). Paraglossa with one or two spine-like setae on inner, distolateral margin. Labial palp with segment I subequal to length of segments II and III combined. Segment II dorsally with row of four or five spine-like setae near outer, distolateral margin. Segment III slightly pentagonal, 0.7× length of segment II. Otherwise, as typical for subgenus.

***Legs*** (Fig. 14a–f). Ratio of leg segments: fore leg 1.0:1.0:0.4:0.2, middle leg 1.0:1.0:0.4:0.2 and hind leg 1.1:1.0:0.4:0.2. ***Femur***. Length ~3 × maximum width. Setae on outer margin long, flattened, pointed. ***Tibia*.** With one row of short, blunt setae on each side of row of long, fine, simple setae. ***Claw*** with one row of 11 or 12 denticles; one posterior seta. Otherwise, as typical for subgenus.

***Abdomen*. *Terga*** (Fig. 14g, h). Posterior margin of terga: I smooth, without denticles; II with rudimentary denticles in middle part, III–IX with triangular, pointed denticles.

***Tergalii*** (Fig. 14i). Elongate oval; tracheation well-developed, not reaching margins, brown; margin smooth, without setae.

***Paraproct*** (Fig. 14j). Posterior margin with prolongation, with split denticles.

***Caudalii*** (Fig. 13i–k). Cerci in middle area with one swimming seta each 2^nd^ segment, distally up to three swimming setae per segment. Paracercus with 13–15 segments.

***Pose of subimaginal gonostyli under larval cuticle*.** Unknown.

**Subimago.** Unknown.

**Imago.** Unknown.

**Egg.** Unknown.

##### Biological aspects.

Larvae living in run of medium fast to fast flowing rivers with both, pristine water (Fig. 37c, d) or influenced by human activities (Fig. 37a).

##### Etymology.

The species name refers to the Fijian word “bula”, meaning hello or welcome, which you hear everywhere from the friendly people in Fiji.

##### Distribution.

Fiji: Viti Levu (Fig. 39d).

#### ﻿ Papuanatula (Fijifiliola) claudia

Taxon classificationAnimaliaEphemeropteraBaetidae

sp. nov.

F7FA939D-52CA-596F-901B-F29529A4F755

https://zoobank.org/C59F4B7D-7CA8-46A7-B1B6-1AA0717F2B2A

Figs 15, 16, 17

##### Material examined.

***Holotype*.** FIJI • larva; Viti Levu, Ba Prov., near Abaca village, Savuione Riv., close below Savuione waterfall; 17°40'26"S, 177°33'06"E; 690 m; 24.x.2024; leg. T. Kaltenbach; on slide; GBIFCH1581938; MZL. ***Paratypes*.** • 9 larvae; same data as holotype; 3 on slides; GBIFCH01581930, GBIFCH01581934, GBIFCH01221828; 6 in alcohol; GBIFCH01581929, GBIFCH01581933, GBIFCH01581935, GBIFCH01581937; MZL.

##### Other material.

FIJI • 14 larvae; Viti Levu, Ba Prov., near Abaca village, Savuione Riv.; 17°40'07"S, 177°32'31"E; 510 m; 24.x.2024; leg. T. Kaltenbach; in alcohol; GBIFCH01581922, GBIFCH01582020; MZL.

##### Diagnosis.

**Larva**. Following combination of characters distinguishes *P. claudia* sp. nov. from other species of Fijifiliola subgen. nov.: abdominal terga I, IX, and X yellowish grey, II–IV dark grey, V–VIII grey with indistinct pattern; femur on anterior side in basal ½ with large, dark reddish brown, hypodermal marking in large blank, marking oblong on fore and mid femur and like a streak on hind femur, distomedially with dark grey transvers band, distally reddish brown and grey; femur on posterior side with large, distomedial, reddish brown, hypodermal streak; labial palp segment III slightly pentagonal; paracercus with 8–10 segments.

##### Description.

**Figure 15.**
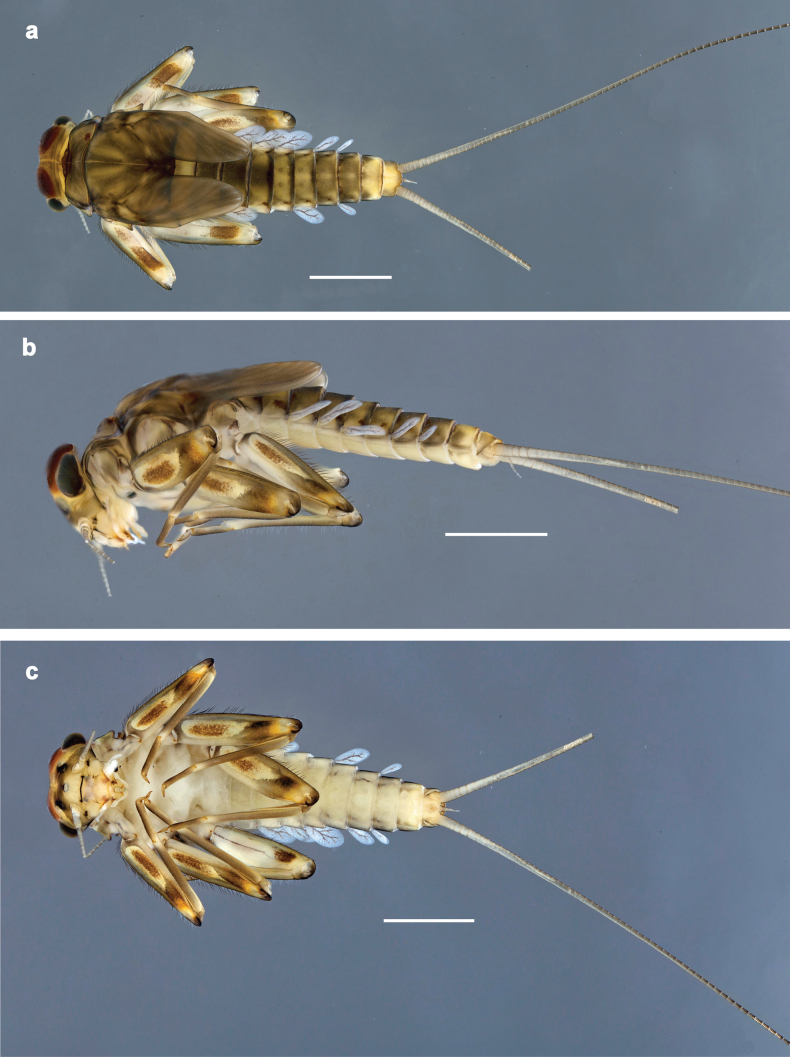
Papuanatula (Fijifiliola) claudia sp. nov., larva, habitus. a. Dorsal view; b. Lateral view; c. Ventral view. Scale bars: 1 mm.

**Figure 16.**
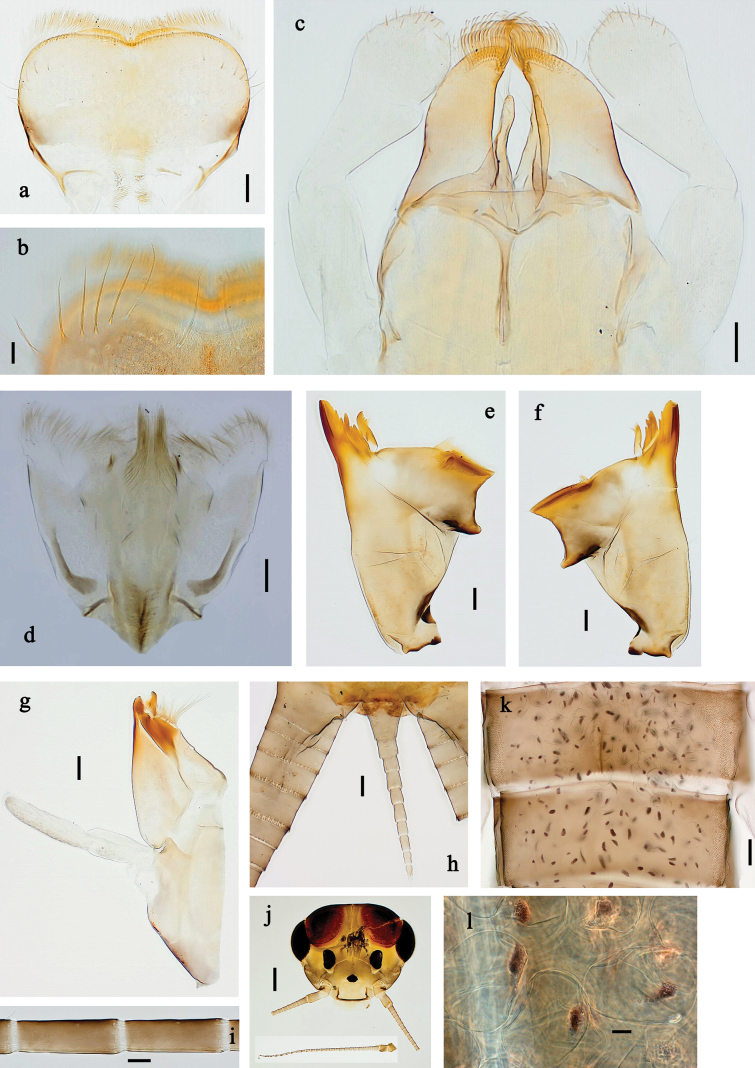
Papuanatula (Fijifiliola) claudia sp. nov., larva. a, b. Labrum; c. Labium; d. Hypopharynx and superlinguae; e. Left mandible; f. Right mandible; g. Maxilla; h. Paracercus; i. Cercus; j. Head; k. Mature female larva, abdominal segments III and IV, eggs; l. Eggs in mature female larva. Scale bars: 100 µm (j); 20 µm (a, c–h); 10 µm (b, i, k, l).

**Figure 17.**
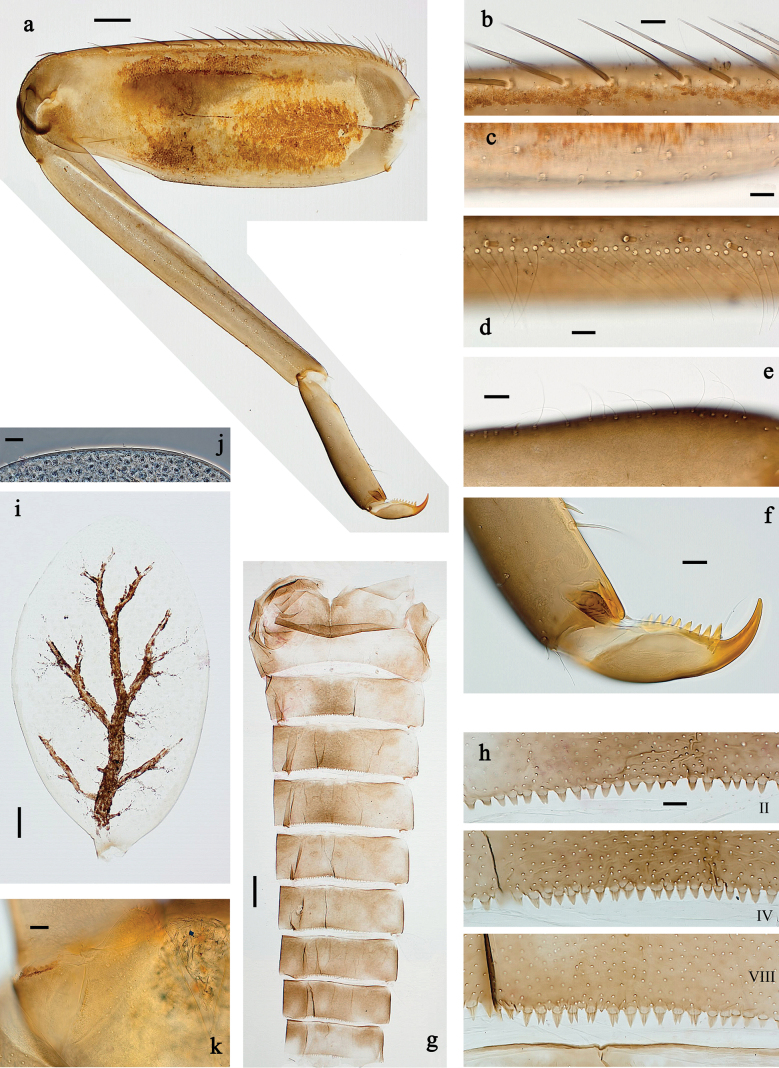
Papuanatula (Fijifiliola) claudia sp. nov., larva. a. Fore leg; b. Femur, outer margin; c. Femur, inner margin; d. Tibia; e. Tarsus, outer margin; f. Tarsus, claw; g. Abdomen; h. Abdominal terga; i, j. Tergalius IV; k. Paraproct. Scale bars: 100 µm (g); 50 µm (a); 20 µm (i); 10 µm (b–f, h, j, k).

**Larva** (Figs 15–17). Body length 4.3–6.0 mm, cerci longer than body length.

***Colouration*** (Figs 15a–c, 17g). Head and thorax dorsally dark grey with indistinct grey pattern; abdominal terga I, IX, and X yellowish grey, II–IV dark grey, V–VIII grey with indistinct pattern. Legs with femur on anterior side in basal ½ with large, dark reddish brown, hypodermal marking in large blank, marking oblong on fore and mid femur, and like a streak on hind femur; distomedially with dark grey transverse band, distally reddish brown and grey in colour; femur on posterior side with large, distomedial, reddish brown, hypodermal streak; tibia and tarsus dark grey. Head, thorax, and abdomen ventrally yellowish grey. Caudalii grey.

***Head*. *Antenna*** (Fig. 16j). Length ~1.5× head length. As typical for subgenus.

***Labrum*** (Fig. 16a, b). Length 0.6× maximum width. Otherwise, as typical for subgenus.

***Right mandible*** (Fig. 16f). Margin between prostheca and mola straight, with few minute denticles. Otherwise, as typical for subgenus.

***Left mandible*** (Fig. 16e). Margin between prostheca and mola straight, with few denticles toward subtriangular process. Otherwise, as typical for subgenus.

***Hypopharynx and superlinguae*** (Fig. 16d). As typical for subgenus.

***Maxilla*** (Fig. 16g). Maxillary palp approx. as long as galea-lacinia; palp segment II ~1.2× longer than segment I. Otherwise, as typical for subgenus.

***Labium*** (Fig. 16c). Paraglossa with one spine-like seta on inner, distolateral margin. Labial palp with segment I ~0.8× length of segments II and III combined. Segment II dorsally with row of five spine-like setae near outer, distolateral margin. Segment III slightly pentagonal, 0.7× length of segment II. Otherwise, as typical for subgenus.

***Legs*** (Fig. 17a–f). Ratio of leg segments: fore leg 1.1:1.0:0.4:0.1, middle leg 1.2:1.0:0.4:0.1 and hind leg 1.2:1.0:0.3:0.1. ***Femur***. Length ~2.7 × maximum width. ***Tibia*.** With one row of short, blunt setae parallel to row of long, fine, simple setae. ***Claw*** with one row of ten or 11 denticles; one posterior seta. Otherwise, as typical for subgenus.

***Abdomen*. *Terga*** (Fig. 17g, h). Posterior margin of terga: I smooth, without denticles; II–IX with triangular, pointed, apically partly split denticles.

***Tergalii*** (Fig. 17i, j). Elongate oval; tracheation well-developed, reaching margins, brown; margin smooth, without setae.

***Paraproct*** (Fig. 17k). Posterior margin with poorly developed prolongation, with split denticles in distal part.

***Caudalii*** (Fig. 16h, i). Cerci without swimming setae, distally with one rudimentary insertion per segment. Paracercus with 8–10 segments.

***Pose of subimaginal gonostyli under larval cuticle*.** Unknown.

**Subimago.** Unknown.

**Imago.** Unknown.

**Egg.** Unknown.

##### Biological aspects.

Local species, larvae living in run of fast flowing, turbulent mountain rivers with pristine water (Fig. 36d). There are male and female larvae, but one last instar female larva had eggs in which the larvae are already developing, indicating facultative parthenogenesis, and probably ovoviviparity (Fig. 16k, l) (see discussion).

##### Etymology.

The species is dedicated to my wife Claudia Kaltenbach for her constant support during this collection trip.

##### Distribution.

Fiji: Viti Levu (Fig. 39e).

#### ﻿ Papuanatula (Fijifiliola) crussetae

Taxon classificationAnimaliaEphemeropteraBaetidae

sp. nov.

648B9C5E-C7EB-586E-A028-5FE183F0080A

https://zoobank.org/036A1BF5-4D13-43D1-A3FC-3FA2C1289BF9

Figs 18, 19, 20

##### Material examined.

***Holotype*.** FIJI • larva; Viti Levu, Ba Prov., near Abaca village, Savuione Riv., close below Savuione waterfall; 17°40'26"S, 177°33'06"E; 690 m; 24.x.2024; leg. T. Kaltenbach; on slides; GBIFCH01581936, GBIFCH01582032; MZL.

##### Diagnosis.

**Larva**. Following combination of characters distinguishes *P. crussetae* sp. nov. from other species of Fijifiliola subgen. nov.: femur with irregular row of medium to long, spine-like setae at outer margin; tibia with irregular row of medium, spine-like setae parallel to row of long, fine, simple setae; distalmost seta at inner margin of tarsus not much longer than other setae; claw with one row of 16–19 denticles; tergalii in large middle part brown; paracercus with five segments.

##### Description.

**Figure 18.**
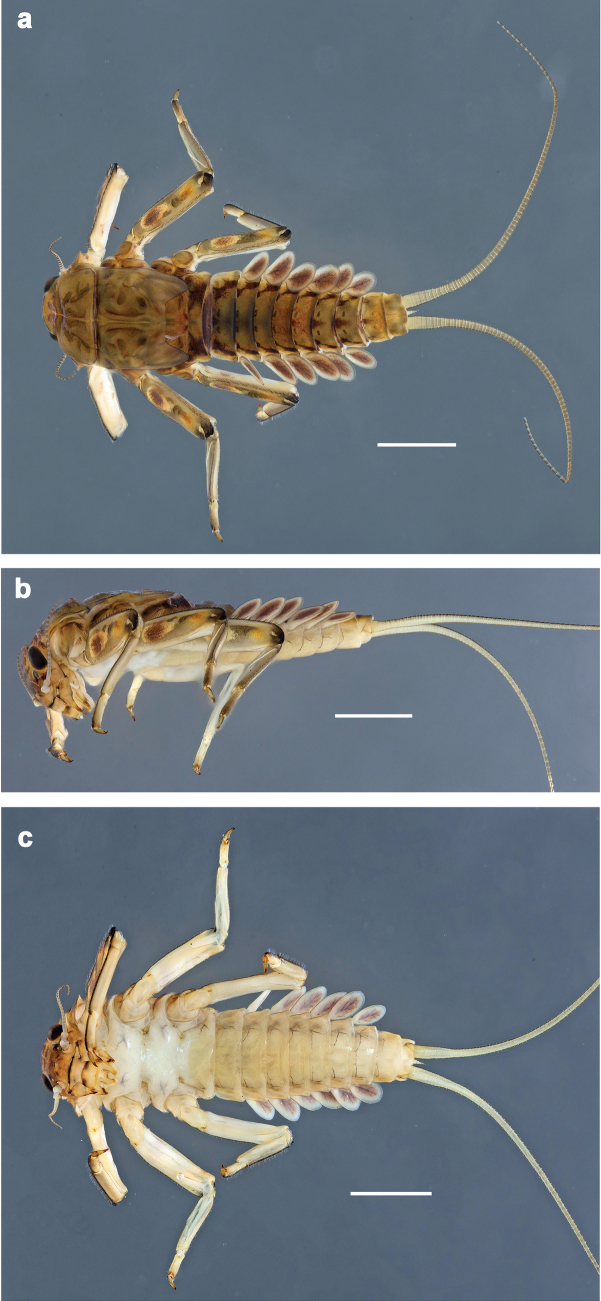
Papuanatula (Fijifiliola) crussetae sp. nov., larva, habitus. a. Dorsal view; b. Lateral view; c. Ventral view. Scale bars: 1 mm.

**Figure 19.**
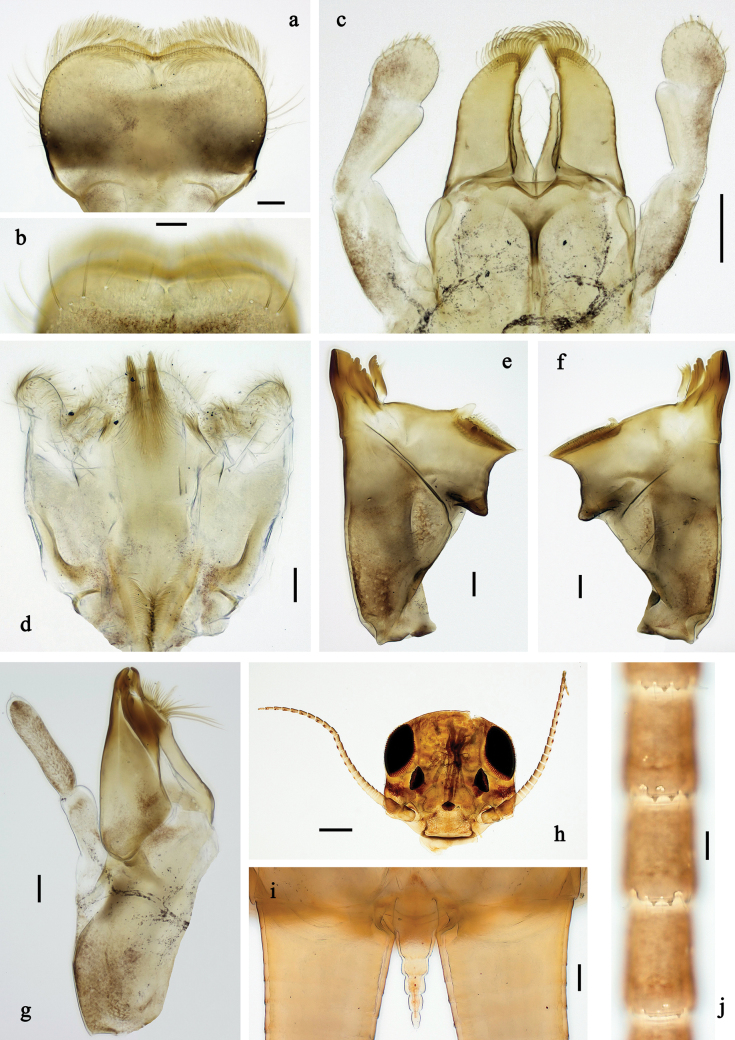
Papuanatula (Fijifiliola) crussetae sp. nov., larva. a, b. Labrum; c. Labium; d. Hypopharynx and superlinguae; e. Left mandible; f. Right mandible; g. Maxilla; h. Head; i. Paracercus; j. Cercus. Scale bars: 100 µm (h); 20 µm (a, c–g, i); 10 µm (b, j).

**Figure 20.**
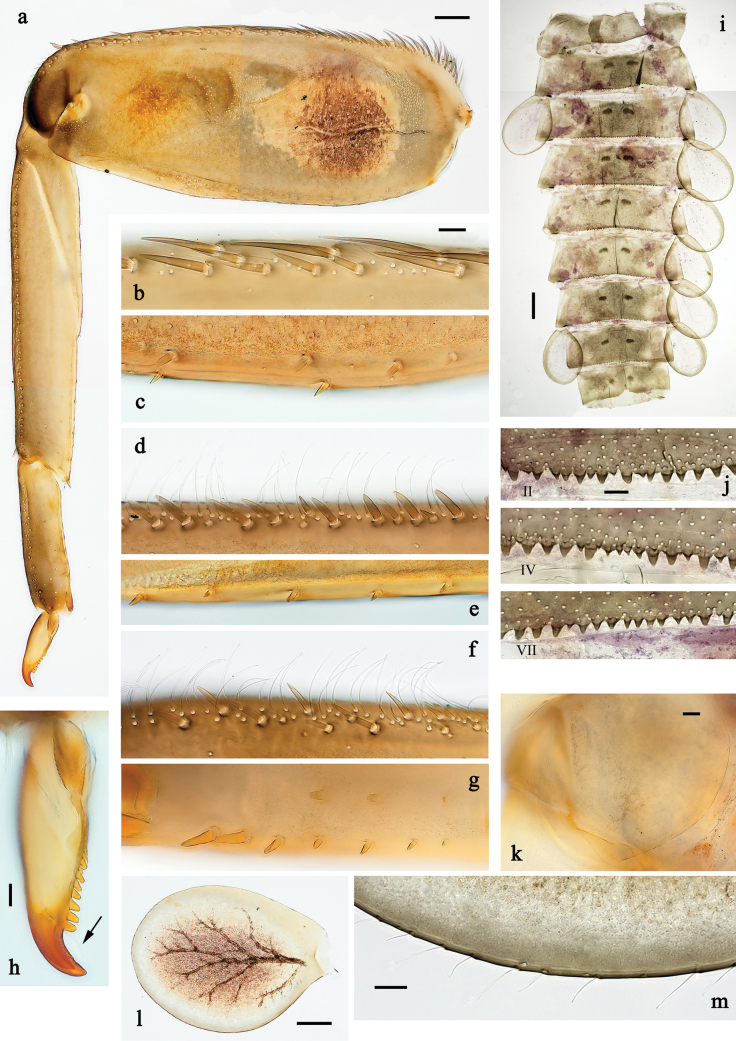
Papuanatula (Fijifiliola) crussetae sp. nov., larva. a. Middle leg; b. Femur, outer margin; c. Femur, inner margin; d. Tibia, outer margin; e. Tibia, inner margin; f. Tarsus, outer margin; g. Tarsus, inner margin; h. Claw (arrow: posterior seta); i. Abdomen; j. Abdominal terga; k. Paraproct; l, m. Tergalius VI. Scale bars: 100 µm (i); 50 µm (a, l); 10 µm (b–h, j, k, m).

**Larva** (Figs 18–20). Body length 4.7 mm, cerci longer than body length.

***Colouration*** (Fig. 18a–c). Head and thorax dorsally dark grey-brown with indistinct grey pattern; abdomen dorsally dark grey-brown to reddish brown, darker in middle part and laterally, terga IX and X brighter. Legs dark grey; femur on anterior side in basal ½ with large, dark brown, hypodermal, oval marking in large blank, distomedially reddish brown. Thorax ventrally off-white, abdomen ventrally yellowish grey. Caudalii grey.

***Head*. *Antenna*** (Fig. 19h). Length ~1.5× head length. As typical for subgenus.

***Labrum*** (Fig. 19a, b). Length 0.7× maximum width. Otherwise, as typical for subgenus.

***Right mandible*** (Fig. 19f). Margin between prostheca and mola straight, with few minute denticles. Otherwise, as typical for subgenus.

***Left mandible*** (Fig. 19e). Margin between prostheca and mola slightly convex, with few minute denticles. Otherwise, as typical for subgenus.

***Hypopharynx and superlinguae*** (Fig. 19d). As typical for subgenus.

***Maxilla*** (Fig. 19g). Maxillary palp approx. as long as galea-lacinia; palp segment II ~1.2× longer than segment I. Otherwise, as typical for subgenus.

***Labium*** (Fig. 19c). Paraglossa with two spine-like setae on inner, distolateral margin. Labial palp with segment I subequal to length of segments II and III combined. Segment II dorsally with row of six spine-like setae near outer, distolateral margin. Segment III slightly pentagonal, 0.8× length of segment II. Otherwise, as typical for subgenus.

***Legs*** (Fig. 20a–g). Ratio of leg segments: fore leg 1.2:1.0:0.5:0.2, middle leg 1.2:1.0:0.4:0.2 and hind leg 1.2:1.0:0.5:0.2. ***Femur***. Length ~2.2× maximum width on foreleg, other legs ~2.5×. Outer margin with irregular row of medium to long, spine-like setae. Short, pointed, spine-like setae along ventral margin. ***Tibia*.** With irregular row of short to medium, spine-like setae parallel to row of long, fine, simple setae. ***Tarsus*.** Outer margin with irregular row of short to medium, spine-like setae parallel to row of long, fine, simple setae, similar to tibia. Distalmost seta at inner margin not much longer than other setae. ***Claw*** with one row of 16–19 denticles; one or two posterior setae. Otherwise, as typical for subgenus.

***Abdomen*. *Terga*** (Fig. 20i, j). Posterior margin of terga: I smooth, without denticles; II–IX with triangular denticles.

***Tergalii*** (Fig. 20l, m). Wide skewed oval in shape; tracheation well-developed, reaching margins, brown; scattered brown hypodermal pigmentation in large middle part. Margin with minute denticles and medium, fine, simple setae.

***Paraproct*** (Fig. 20k). Posterior margin without prolongation, smooth, without denticles. Cercotractor without denticles.

***Caudalii*** (Fig. 19i, j). Cerci without swimming setae. Paracercus with five segments.

***Pose of subimaginal gonostyli under larval cuticle*.** Unknown.

**Subimago.** Unknown.

**Imago.** Unknown.

**Egg.** Unknown.

##### Biological aspects.

Local species; larvae living in run of fast flowing, turbulent mountain rivers with pristine water (Fig. 36d).

##### Etymology.

The species name is based on the Latin words crus and setae, meaning leg bristles, referring to the extraordinary setation of the legs.

##### Distribution.

Fiji: Viti Levu (Fig. 39f).

#### ﻿ Papuanatula (Fijifiliola) flowersi

Taxon classificationAnimaliaEphemeropteraBaetidae

sp. nov.

2035864D-4490-56DC-B9FF-4955E3DE41C1

https://zoobank.org/DA210268-B574-41CC-8655-D11741506A08

Figs 21, 22, 23

##### Material examined.

***Holotype*.** FIJI • larva; Viti Levu, Ba Prov., near Abaca village, Savuione Riv., close below Savuione waterfall; 17°40'26"S, 177°33'06"E; 690 m; 24.x.2024; leg. T. Kaltenbach; on slide; GBIFCH01581931; MZL. ***Paratypes*.** • 1 larva; same data as holotype; on slide; GBIFCH01581932; MZL • 1 larva; Viti Levu, Ba Prov., near Abaca village, Savuione Riv.; 17°40'07"S, 177°32'31"E; 510 m; 24.x.2024; leg. T. Kaltenbach; on slide; GBIFCH01581952; MZL.

##### Diagnosis.

**Larva**. Following combination of characters distinguishes *P. flowersi* sp. nov. from other species of Fijifiliola subgen. nov.: head orange-brown; abdomen dorsally dark brown, terga III–V partly orange-brown, X orange-brown; femur dark orange-brown; apex of hypopharynx without bifid bunch of stout setae; labial palp segment III elongate conical, inner margin straight; tergalii with orange-brown pigmentation outside tracheation; paracercus with 13 segments.

##### Description.

**Figure 21.**
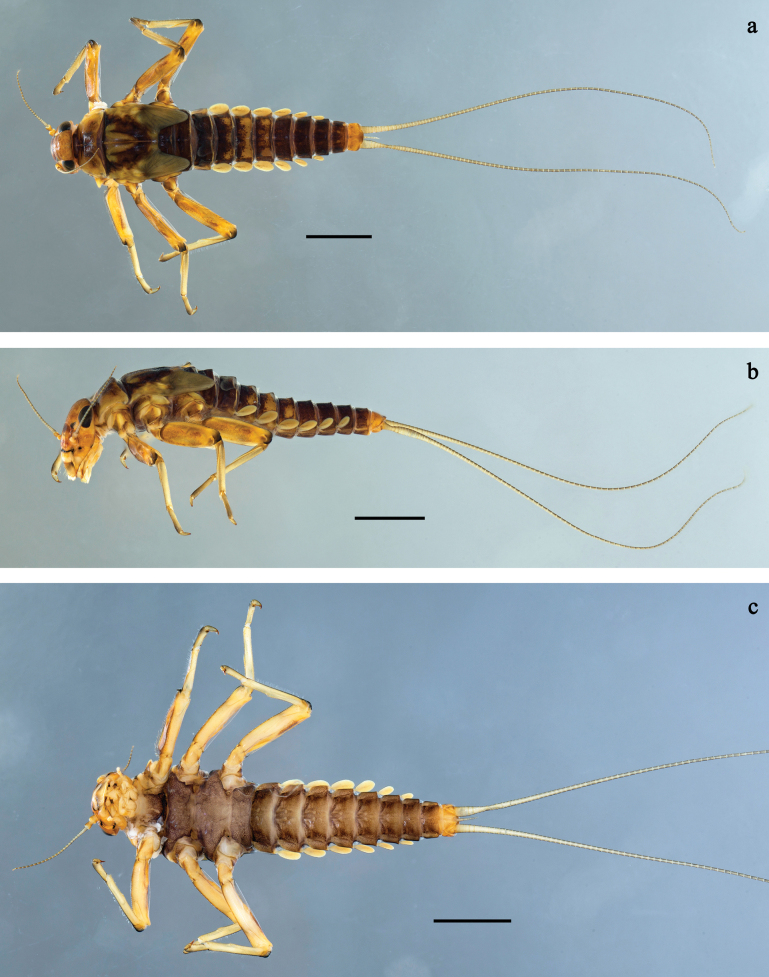
Papuanatula (Fijifiliola) flowersi sp. nov., larva, habitus. a. Dorsal view; b. Lateral view; c. Ventral view. Scale bars: 1 mm.

**Figure 22.**
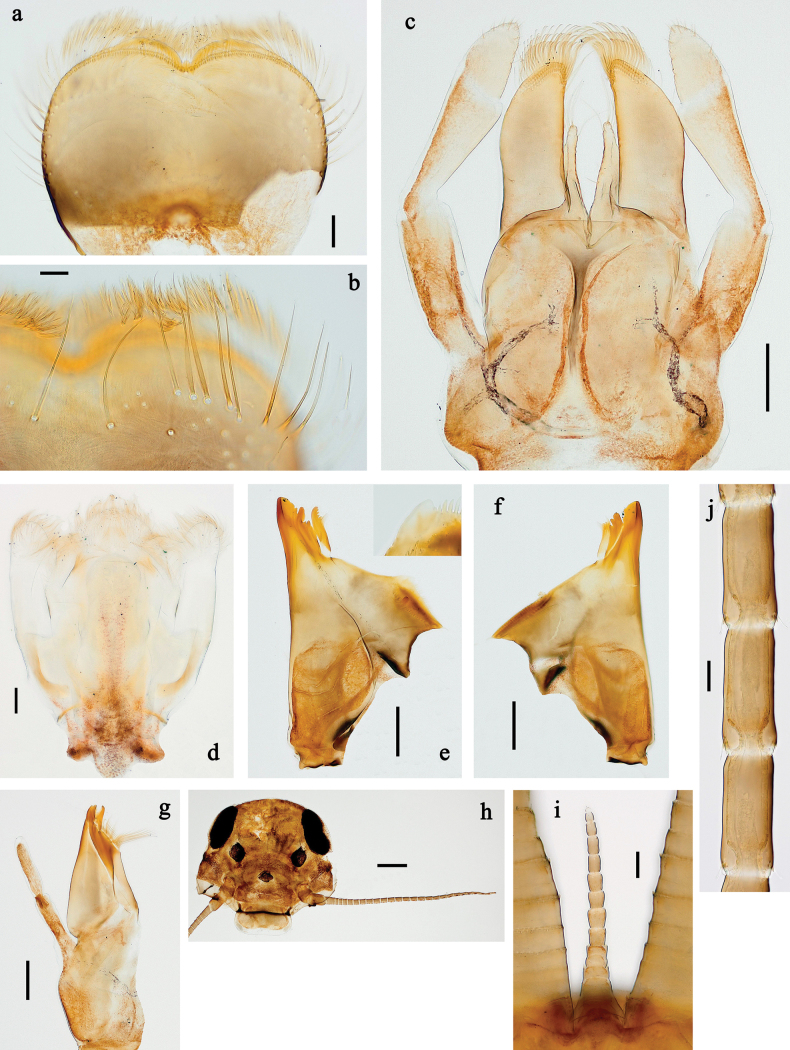
Papuanatula (Fijifiliola) flowersi sp. nov., larva. a, b. Labrum; c. Labium; d. Hypopharynx and superlinguae; e. Left mandible (detail: subtriangular process); f. Right mandible; g. Maxilla; h. Head; i. Paracercus; j. Cercus. Scale bars: 100 µm (h); 20 µm (a, c–g, i); 10 µm (b, j).

**Figure 23.**
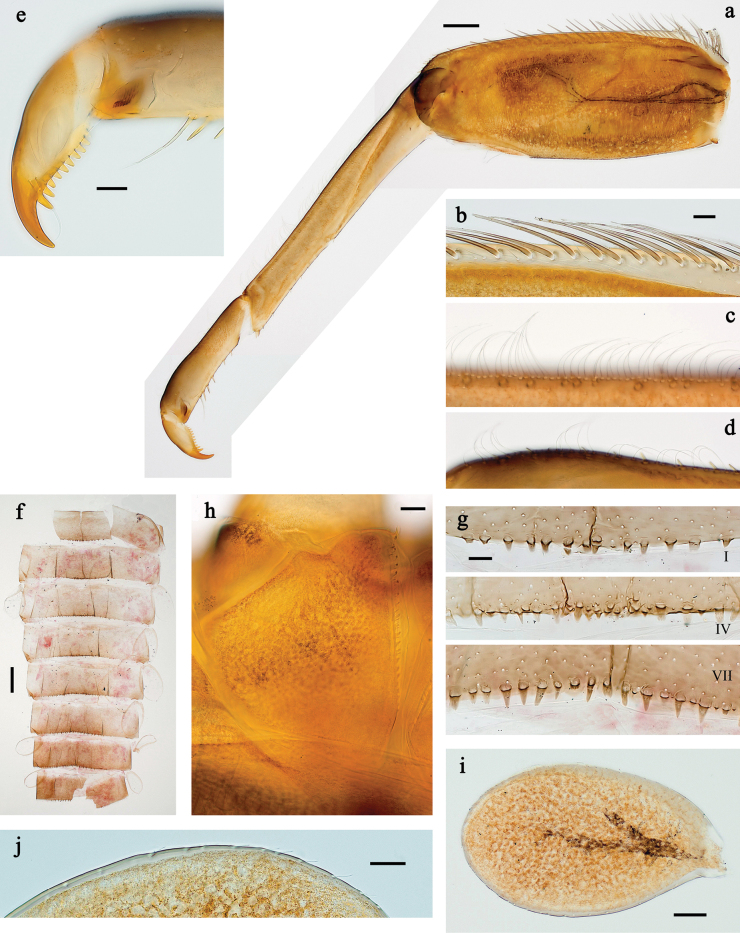
Papuanatula (Fijifiliola) flowersi sp. nov., larva. a. Fore leg; b. Femur, outer margin; c. Tibia; d. Tarsus, outer margin; e. Tarsus, claw; f. Abdomen; g. Abdominal terga; h. Paraproct; i, j. Tergalius IV. Scale bars: 100 µm (f); 50 µm (a); 20 µm (i); 10 µm (b–e, g, h, j).

**Larva** (Figs 21–23). Body length 4.9–5.6 mm, cerci ~1.2× body length.

***Colouration*** (Fig. 21a–c). Head orange-brown; thorax dorsally dark grey-brown with indistinct grey pattern, fore protoptera grey; abdomen dorsally dark brown, terga III–V partly orange-brown, X orange-brown. Legs mainly orange-brown, femur apically dark brown. Thorax and abdomen ventrally dark grey, segment X orange-brown. Caudalii orange-brown.

***Head*. *Antenna*** (Fig. 22h). Length ~1.5× head length. As typical for subgenus.

***Labrum*** (Fig. 22a, b). Length 0.7× maximum width. Otherwise, as typical for subgenus.

***Right mandible*** (Fig. 22f). Margin between prostheca and mola straight, smooth. Otherwise, as typical for subgenus.

***Left mandible*** (Fig. 22e). Margin between prostheca and mola straight, with few minute denticles toward subtriangular process. Subtriangular process short. Otherwise, as typical for subgenus.

***Hypopharynx and superlinguae*** (Fig. 22d). Apex of lingua without bifid bunch of stout setae. Otherwise, as typical for subgenus.

***Maxilla*** (Fig. 22g). Maxillary palp approx. as long as galea-lacinia; palp segment II ~0.9× length of segment I. Otherwise, as typical for subgenus.

***Labium*** (Fig. 22c). Paraglossa with two spine-like setae on inner, distolateral margin. Labial palp with segment I ~0.8× length of segments II and III combined. Segment II dorsally with row of five or six spine-like setae near outer, distolateral margin. Segment III elongate conical, 0.5× length of segment II, inner margin straight, outer margin convex. Otherwise, as typical for subgenus.

***Legs*** (Fig. 23a–e). Ratio of leg segments: fore leg 1.0:1.0:0.5:0.2, middle leg 1.0:1.0:0.4:0.2 and hind leg 1.0:1.0:0.4:0.2. ***Femur***. Length ~2.5× maximum width. ***Tibia*.** With row of short, blunt setae on each side parallel to row of long, fine, simple setae. ***Claw*** with one row of 9–12 denticles; one posterior seta. Otherwise, as typical for subgenus.

***Abdomen*. *Terga*** (Fig. 23f, g). Posterior margin of terga: I–IX with triangular, pointed denticles, becoming longer toward end of abdomen.

***Tergalii*** (Fig. 23i, j). Oval; tracheation mainly limited to main trunk, dark brown; with scattered, orange-brown, hypodermal pigmentation. Margin with minute denticles and short, fine, simple setae.

***Paraproct*** (Fig. 23h). Posterior margin with prolongation, with denticles.

***Caudalii*** (Fig. 22i, j). Cerci without swimming setae. Paracercus with 13 segments.

***Pose of subimaginal gonostyli under larval cuticle*.** Unknown.

**Subimago.** Unknown.

**Imago.** Unknown.

**Egg.** Unknown.

##### Biological aspects.

Local species; larvae living in run of fast flowing, turbulent mountain rivers with pristine water (Fig. 36d, e).

##### Etymology.

The species is dedicated to R.W. Flowers (formerly Florida A & M University, Tallahassee, USA), who pioneered the work on Fijian mayflies with his article dated 1990.

##### Distribution.

Fiji: Viti Levu (Fig. 39e).

#### ﻿ Papuanatula (Fijifiliola) gattolliati

Taxon classificationAnimaliaEphemeropteraBaetidae

sp. nov.

C992ED63-8E92-53DA-8E21-46D5C18F23E1

https://zoobank.org/A86F9447-E231-4BDA-868F-65CB2BF88677

Figs 24, 25, 26

##### Material examined.

***Holotype*.** FIJI • larva; Vanua Levu, Cacaudrove Prov., Seaqaqa Riv., bridge near Saivou village; 16°36'25"S, 179°08'46"E; 80 m; 05.xi.2024; leg. T. Kaltenbach; on slide; GBIFCH01581988; MZL. ***Paratypes*.** • 2 larvae; same data as holotype; on slides; GBIFCH01581983, GBIFCH01581989; MZL • 8 larvae; Vanua Levu, Cacaudrove Prov., tributary to Sekawa Riv., near Nakawaga; 16°39'55"S, 179°20'03"E; 32 m; 29.x.2024; leg. T. Kaltenbach; 1 on slide; GBIFCH01581907; 7 in alcohol; GBIFCH01581901, GBIFCH01581906, GBIFCH01582015; MZL.

##### Diagnosis.

**Larva**. Following combination of characters distinguishes *P. gattolliati* sp. nov. from other species of Fijifiliola subgen. nov.: larva dorsally grey, abdominal segments laterally with blackish marking; femur on anterior side in basal ½ with reddish brown marking in large blank (well-developed on fore femur, less on middle femur and absent on hind femur), large dark grey band in middle area, reddish brown colour in subdistal part; labial palp segment III slightly pentagonal; paracercus with 12–14 segments.

##### Description.

**Figure 24.**
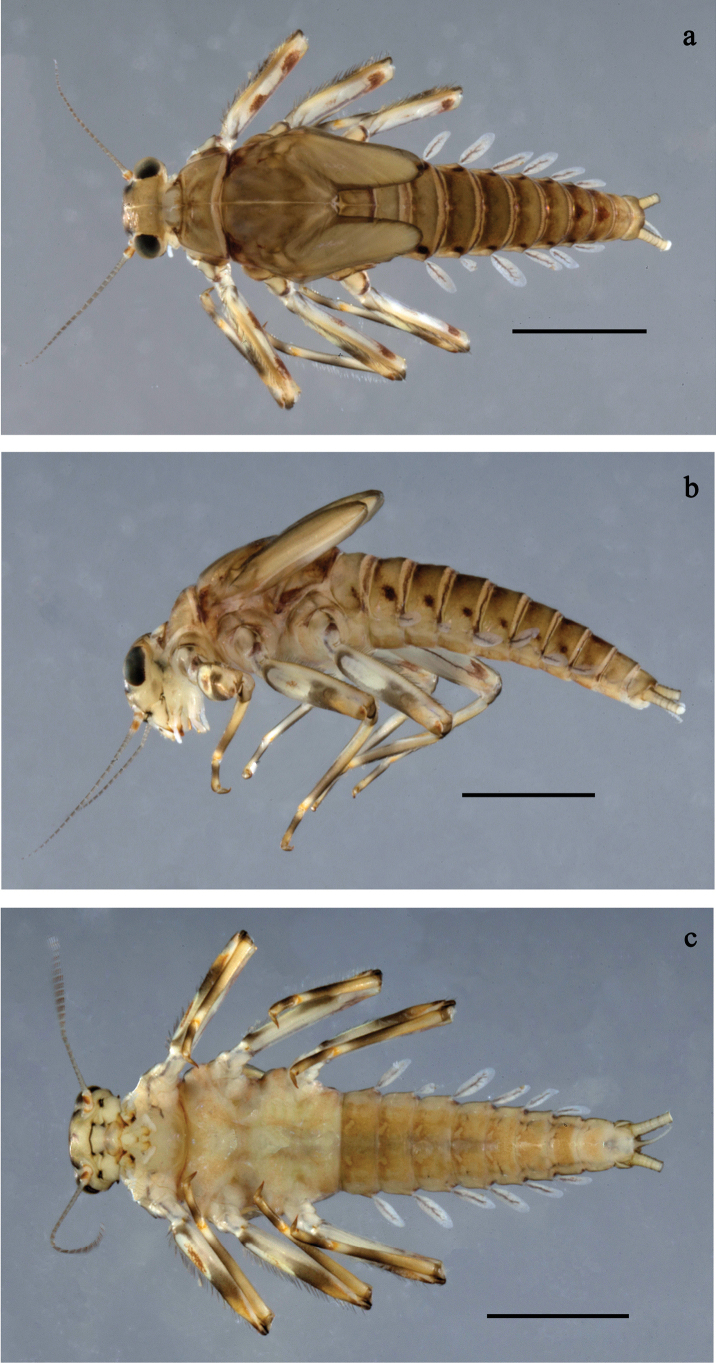
Papuanatula (Fijifiliola) gattolliati sp. nov., larva, habitus. a. Dorsal view; b. Lateral view; c. Ventral view. Scale bars: 1 mm.

**Figure 25.**
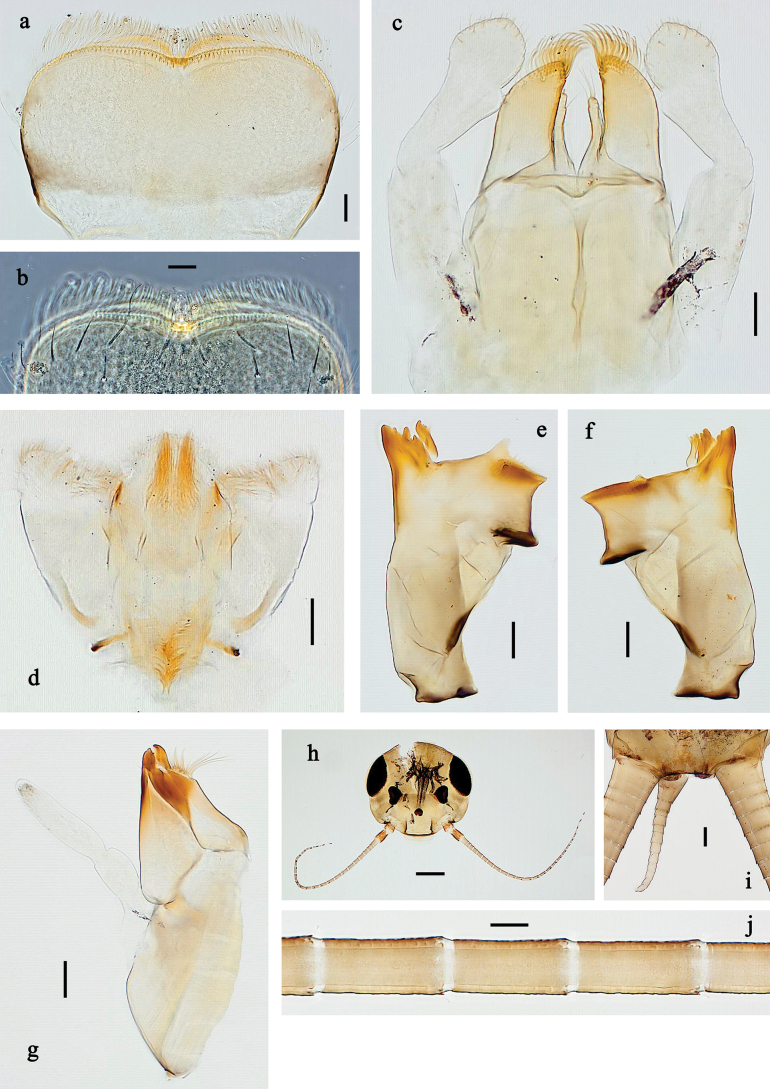
Papuanatula (Fijifiliola) gattolliati sp. nov., larva. a, b. Labrum; c. Labium; d. Hypopharynx and superlinguae; e. Left mandible; f. Right mandible; g. Maxilla; h. Head; i. Paracercus; j. Cercus. Scale bars: 100 µm (h); 20 µm (a, c–g, i); 10 µm (b, j).

**Figure 26.**
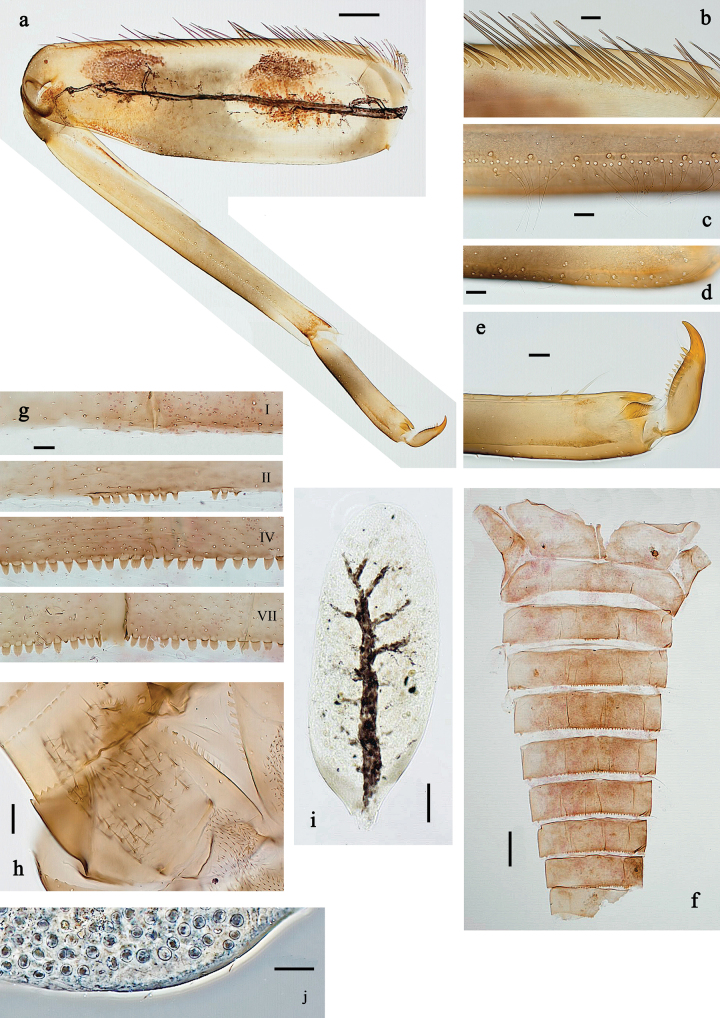
Papuanatula (Fijifiliola) gattolliati sp. nov., larva. a. Fore leg; b. Femur, outer margin; c. Tibia; d. Tarsus, outer margin; e. Tarsus, claw; f. Abdomen; g. Abdominal terga; h. Paraproct; i, j. Tergalius IV. Scale bars: 100 µm (f); 50 µm (a); 20 µm (i); 10 µm (b–e, g, h, j).

**Larva** (Figs 24–26). Body length 2.9–4.4 mm, cerci ~1.5× body length.

***Colouration*** (Fig. 24a–c). Head and thorax dorsally grey with indistinct pattern, fore protoptera grey with dark grey margin; abdomen dorsally grey, terga III and IV darker, terga II and V–VIII darker in anterior part, abdominal segments laterally with oblique, blackish marking. Femur on anterior side in basal ½ with reddish brown marking in large blank (well-developed on fore femur, less on middle femur and absent on hind femur), large dark grey band in middle area, basal and apical margin dark grey, reddish brown colour in subdistal part; femur on posterior side with dark brown marking posteromedially and distomedially, less developed on middle and hind legs; tibia reddish grey in basal ½ and distally, dark grey in distomedial part; tarsus basally and distally reddish grey, medially dark grey; claw dark grey. Head and thorax ventrally yellowish grey, abdomen ventrally grey-brown. Caudalii grey.

***Head*. *Antenna*** (Fig. 25h). Length ~2× head length. As typical for subgenus.

***Labrum*** (Fig. 25a, b). Length 0.5× maximum width. Otherwise, as typical for subgenus.

***Right mandible*** (Fig. 25f). Margin between prostheca and mola straight, smooth. Otherwise, as typical for subgenus.

***Left mandible*** (Fig. 25e). Margin between prostheca and mola straight, with few minute denticles toward subtriangular process. Otherwise, as typical for subgenus.

***Hypopharynx and superlinguae*** (Fig. 25d). As typical for subgenus.

***Maxilla*** (Fig. 25g). Maxillary palp slightly longer than galea-lacinia; palp segment II ~1.3× length of segment I. Otherwise, as typical for subgenus.

***Labium*** (Fig. 25c). Paraglossa with two spine-like setae on inner, distolateral margin. Labial palp with segment I approx. as long as segments II and III combined. Segment II dorsally with row of four or five spine-like setae near outer, distolateral margin. Segment III slightly pentagonal, 0.6× length of segment II. Otherwise, as typical for subgenus.

***Legs*** (Fig. 26a–e). Ratio of leg segments: fore leg 1.0:1.0:0.4:0.2, middle leg 1.1:1.0:0.4:0.2 and hind leg 1.2:1.0:0.3:0.1. ***Femur***. Length ~3× maximum width. ***Tibia*.** With row of short, blunt setae parallel to row of long, fine, simple setae. ***Claw*** with one row of 11 or 12 denticles; one posterior seta. Otherwise, as typical for subgenus.

***Abdomen*. *Terga*** (Fig. 26f, g). Posterior margin of terga: I smooth, without denticles, II–IX with triangular, pointed denticles.

***Tergalii*** (Fig. 26i, j). Narrow oblong; tracheation well-developed, partly reaching margins, dark brown. Margin smooth, with few short, fine, simple setae.

***Paraproct*** (Fig. 26h). Posterior margin with prolongation, with split denticles.

***Caudalii*** (Fig. 25i, j). Cerci without swimming setae. Paracercus with 12–14 segments.

***Pose of subimaginal gonostyli under larval cuticle*.** As typical for subgenus.

**Subimago.** Unknown.

**Imago.** Unknown.

**Egg.** Unknown.

##### Biological aspects.

Larvae living in run or riffles of medium fast flowing lowland rivers (Fig. 38a, c).

##### Etymology.

The species is dedicated to Jean-Luc Gattolliat (Naturéum, Muséum cantonal des Sciences Naturelles, Lausanne, Switzerland), for his long-time contribution to the knowledge of Baetidae, and for teaching me many things about mayflies.

##### Distribution.

Fiji: Vanua Levu (Fig. 40a).

#### ﻿ Papuanatula (Fijifiliola) grisea

Taxon classificationAnimaliaEphemeropteraBaetidae

sp. nov.

D358252C-08B1-5275-A4E5-A747C26DE594

https://zoobank.org/0ACCF107-AF4B-437B-8580-3AAD24F27989

Figs 27, 28, 29

##### Material examined.

***Holotype*.** FIJI • larva; Viti Levu, Ba Prov., Ba Riv., near Outback Hotel; 17°37'35"S, 177°45'04"E; 20 m; 22.x.2024; leg. T. Kaltenbach; on slide; GBIFCH01582000; MZL. ***Paratypes*.** • 4 larvae; same data as holotype; 3 on slides; GBIFCH01581958, GBIFCH01581959, GBIFCH01221840, GBIFCH01221836 (gonostylus); 1 in alcohol; GBIFCH01582030; MZL • 4 larvae; Viti Levu, Ba Prov., Ba Riv., near Outback Hotel; 17°37'35"S, 177°45'04"E; 20 m; 21.x.2024; leg. T. Kaltenbach; 1 on slide; GBIFCH01581940; 2 in alcohol; GBIFCH01582011, GBIFCH01582029; MZL • 2 larvae; Viti Levu, Ba Prov., Sabeto Riv., near Sabeto; 17°43'09"S, 177°31'21"E; 30 m; 17.xi.2024; leg. T. Kaltenbach; 1 on slide; GBIFCH01581972; 1 in alcohol; GBIFCH01582026; MZL.

##### Diagnosis.

**Larva**. Following combination of characters distinguishes *P. grisea* sp. nov. from other species of Fijifiliola subgen. nov.: larva dorsally grey; legs grey, femur on anterior side in basal part with reddish brown, hypodermal streak (poorly developed on middle leg, nearly absent on hind leg) in large, wedge-shaped blank; labial palp segment III slightly pentagonal; paracercus with 10–15 segments; cerci with 1–5 swimming setae in middle part.

##### Description.

**Figure 27.**
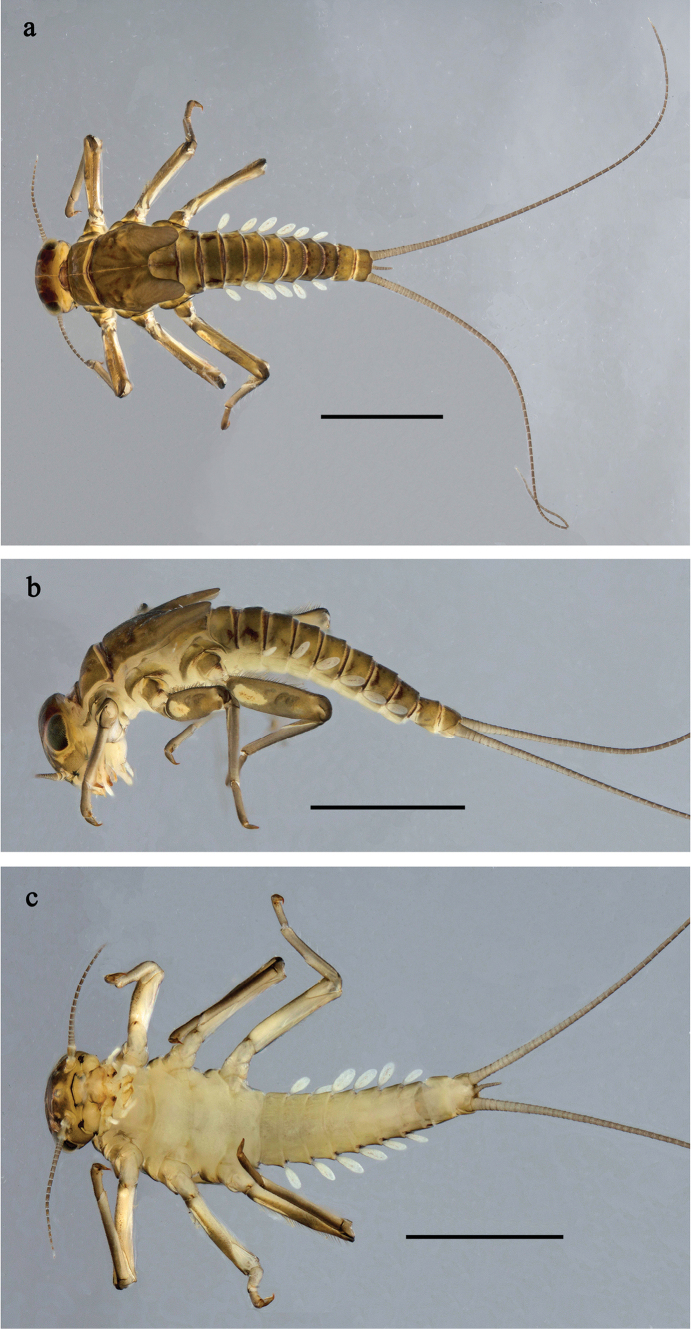
Papuanatula (Fijifiliola) grisea sp. nov., larva, habitus. a. Dorsal view; b. Lateral view; c. Ventral view. Scale bars: 1 mm.

**Figure 28.**
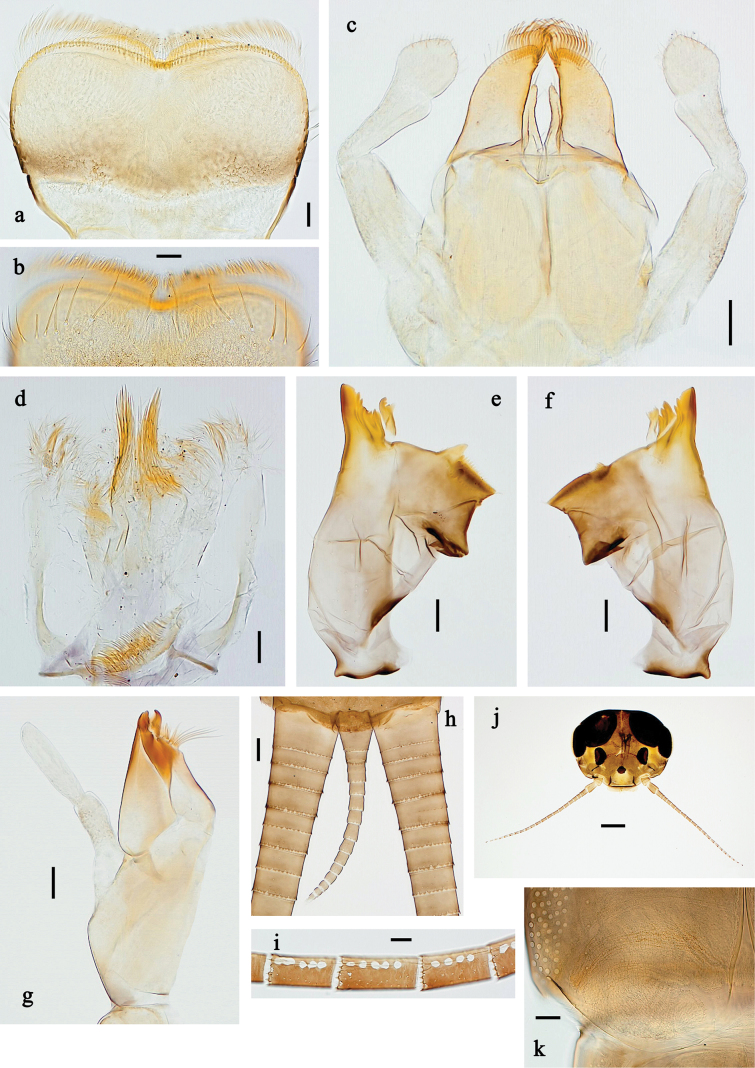
Papuanatula (Fijifiliola) grisea sp. nov., larva. a, b. Labrum; c. Labium; d. Hypopharynx and superlinguae; e. Left mandible; f. Right mandible; g. Maxilla; h. Paracercus; i. Cercus; j. Head; k. Male mature larva, developing subimaginal gonostylus. Scale bars: 100 µm (j); 50 µm (c); 20 µm (e–h); 10 µm (b, d, i, k).

**Figure 29.**
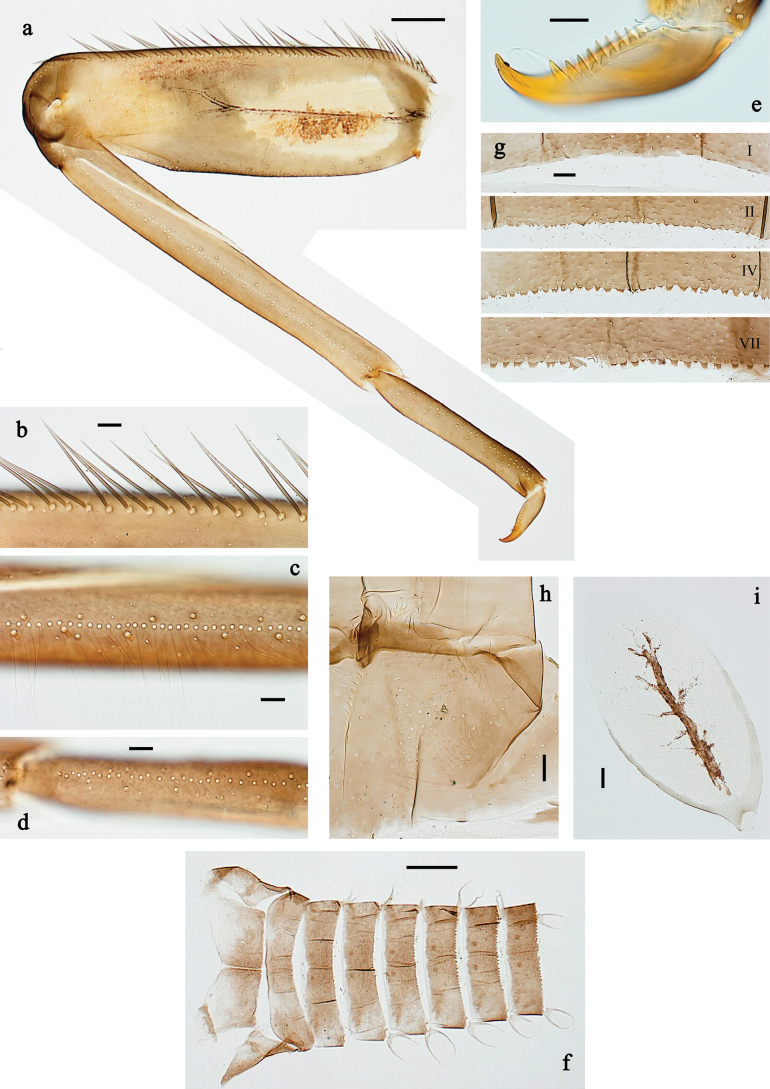
Papuanatula (Fijifiliola) grisea sp. nov., larva. a. Fore leg; b. Femur, outer margin; c. Tibia; d. Tarsus; e. Claw; f. Abdomen; g. Abdominal terga; h. Paraproct; i. Tergalius IV. Scale bars: 100 µm (f); 50 µm (a); 10 µm (b–e, g–i).

**Larva** (Figs 27–29). Body length 3.0–3.4 mm, cerci subequal to body length.

***Colouration*** (Fig. 27a–c). Head, thorax and abdomen dorsally grey with indistinct pattern as in Fig. 27a, fore protoptera grey. Legs grey, femur on anterior side in basal part with reddish brown, hypodermal streak (poorly developed on middle leg, nearly absent on hind leg) in large, wedge-shaped blank, medially with dark brown marking; femur on posterior side yellowish grey, without marking. Head, thorax and abdomen ventrally light grey. Caudalii grey.

***Head*. *Antenna*** (Fig. 28j). Length ~1.5× head length. As typical for subgenus.

***Labrum*** (Fig. 28a, b). Length 0.5× maximum width. Otherwise, as typical for subgenus.

***Right mandible*** (Fig. 28f). Margin between prostheca and mola straight, with few minute denticles. Otherwise, as typical for subgenus.

***Left mandible*** (Fig. 28e). Margin between prostheca and mola straight, with few minute denticles toward subtriangular process. Otherwise, as typical for subgenus.

***Hypopharynx and superlinguae*** (Fig. 28d). As typical for subgenus.

***Maxilla*** (Fig. 28g). Maxillary palp slightly longer than galea-lacinia; palp segment II subequal to length of segment I. Otherwise, as typical for subgenus.

***Labium*** (Fig. 28c). Paraglossa with one spine-like seta on inner, distolateral margin. Labial palp with segment I ~1.4× length of segments II and III combined. Segment II dorsally with row of four spine-like setae near outer, distolateral margin. Segment III slightly pentagonal, 0.6× length of segment II. Otherwise, as typical for subgenus.

***Legs*** (Fig. 29a–e). Ratio of leg segments: fore leg 0.9:1.0:0.3:0.2, middle leg 1.0:1.0:0.4:0.2 and hind leg 1.1:1.0:0.4:0.2. ***Femur***. Length ~3× maximum width. ***Tibia*.** With row of short, blunt setae on each side parallel to row of long, fine, simple setae. ***Claw*** with one row of 10–12 denticles; one posterior setae. Otherwise, as typical for subgenus.

***Abdomen*. *Terga*** (Fig. 29f, g). Posterior margin of terga: I smooth, without denticles, II–IX with triangular, partly split denticles.

***Tergalii*** (Fig. 29i). Elongate oval; tracheation mainly limited to main trunk, dark brown. Margin smooth, with short, fine, simple setae.

***Paraproct*** (Fig. 29h). Posterior margin with prolongation, with split denticles.

***Caudalii*** (Fig. 28h, i). Cerci with 1–5 swimming setae in middle part. Paracercus with 10–15 segments.

***Pose of subimaginal gonostyli under larval cuticle*** (Fig. 28k). As typical for subgenus.

**Subimago.** Unknown.

**Imago.** Unknown.

**Egg.** Unknown.

##### Biological aspects.

Larvae living in run or riffles of medium fast flowing lowland rivers (Figs 36b, c, 37b).

##### Etymology.

The species name refers to the predominant grey colour, which distinguishes it easily from the often co-occurring species *P. aji* sp. nov.

##### Distribution.

Fiji: Viti Levu (Fig. 40b).

#### ﻿ Papuanatula (Fijifiliola) sekawa

Taxon classificationAnimaliaEphemeropteraBaetidae

sp. nov.

E0640DB9-8ED6-5F88-851A-C3932204375F

https://zoobank.org/669EFAE4-F87D-4F81-8157-F6C69174B477

Figs 30, 31, 32

##### Material examined.

***Holotype*.** FIJI • larva; Vanua Levu, Cacaudrove Prov., tributary to Sekawa Riv., near Nakawaga; 16°39'55"S, 179°20'03"E; 32 m; 29.x.2024; leg. T. Kaltenbach; on slide; GBIFCH01581905; MZL. ***Paratypes*.** • 8 larvae; same data as holotype; 3 on slides; GBIFCH01581904, GBIFCH01581908, GBIFCH01221818; 5 in alcohol; GBIFCH01581902, GBIFCH01581903; MZL.

##### Diagnosis.

**Larva**. Following combination of characters distinguishes *P. sekawa* sp. nov. from other species of Fijifiliola subgen. nov.: larva dorsally dark grey, abdominal tergum X reddish brown; femur on anterior side with large grey area in middle part, in basal ⅓ with dark grey, narrow, longitudinal streak in large blank, distal ⅓ reddish brown; labial palp segment III slightly pentagonal; paracercus with 12 segments; cerci with one or two swimming setae per segment in distomedial part.

##### Description.

**Figure 30.**
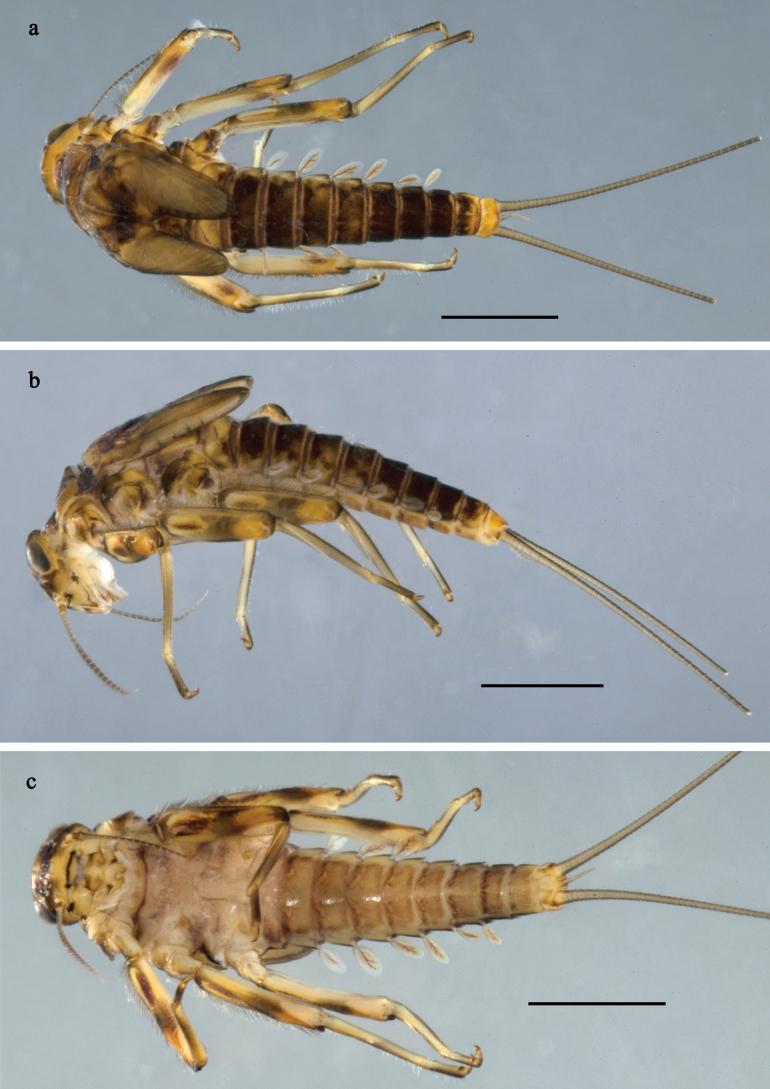
Papuanatula (Fijifiliola) sekawa sp. nov., larva, habitus. a. Dorsal view; b. Lateral view; c. Ventral view. Scale bars: 1 mm.

**Figure 31.**
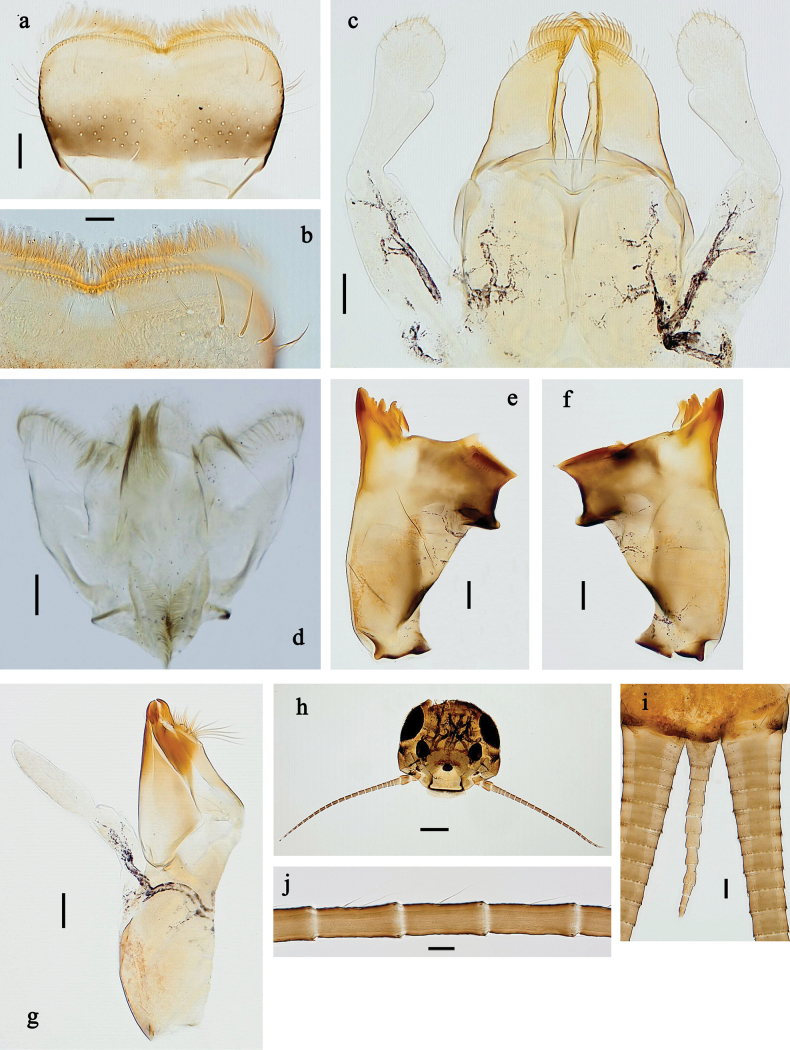
Papuanatula (Fijifiliola) sekawa sp. nov., larva. a, b. Labrum; c. Labium; d. Hypopharynx and superlinguae; e. Left mandible; f. Right mandible; g. Maxilla; h. Head; i. Paracercus; j. Cercus. Scale bars: 100 µm (h); 20 µm (a, c–g, i); 10 µm (b, j).

**Figure 32.**
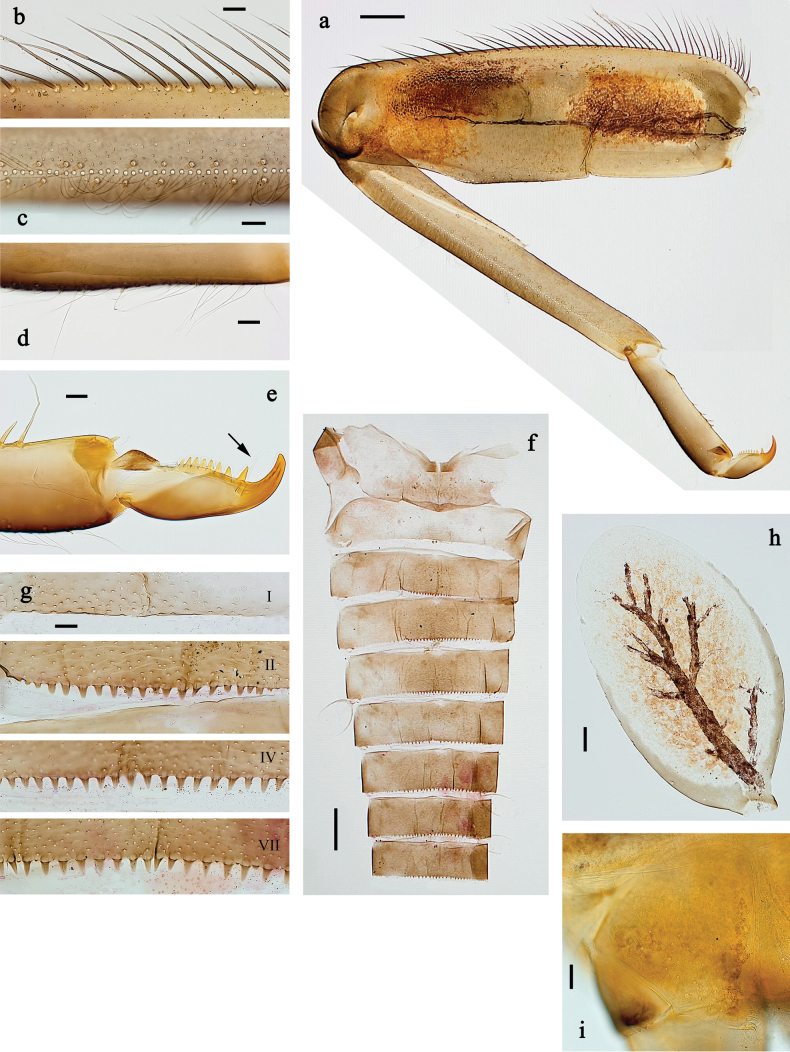
Papuanatula (Fijifiliola) sekawa sp. nov., larva. a. Hind leg; b. Femur, outer margin; c. Tibia; d. Tarsus, outer margin; e. Tarsus, claw; f. Abdomen; g. Abdominal terga; h. Tergalius III; i. Paraproct. Scale bars: 100 µm (f); 50 µm (a); 10 µm (b–e, g–i).

**Larva** (Figs 30–32). Body length 3.6–4.1 mm, cerci broken.

***Colouration*** (Fig. 30a–c). Head, thorax and abdomen dorsally dark grey, thorax with indistinct pattern, fore protoptera dark grey; abdominal tergum I brighter, tergum X reddish brown. Femur on anterior side with large grey area in middle part, in basal ⅓ with dark grey, narrow, longitudinal streak in large blank, distal ⅓ reddish brown; femur on posterior side with dark brown distomedial marking surrounded by reddish brown colour; tibia and tarsus predominantly grey. Thorax and abdomen ventrally grey-brown, sternum X yellow-brown. Caudalii grey.

***Head*. *Antenna*** (Fig. 31h). Length ~1.5× head length. As typical for subgenus.

***Labrum*** (Fig. 31a, b). Length 0.5× maximum width. Otherwise, as typical for subgenus.

***Right mandible*** (Fig. 31f). Margin between prostheca and mola straight, with minute denticles. Otherwise, as typical for subgenus.

***Left mandible*** (Fig. 31e). Margin between prostheca and mola slightly concave, with minute denticles toward subtriangular process. Otherwise, as typical for subgenus.

***Hypopharynx and superlinguae*** (Fig. 31d). As typical for subgenus.

***Maxilla*** (Fig. 31g). Maxillary palp slightly longer as galea-lacinia; palp segment II ~1.3× length of segment I. Otherwise, as typical for subgenus.

***Labium*** (Fig. 31c). Paraglossa with two spine-like setae on inner, distolateral margin. Labial palp with segment I approx. as long as segments II and III combined. Segment II dorsally with row of four or five spine-like setae near outer, distolateral margin. Segment III slightly pentagonal, 0.6× length of segment II. Otherwise, as typical for subgenus.

***Legs*** (Fig. 32a–e). Ratio of leg segments: fore leg 1.0:1.0:0.4:0.1, middle leg 1.0:1.0:0.3:0.1 and hind leg 1.1:1.0:0.4:0.1. ***Femur***. Length ~3× maximum width. Setae on outer margin long, flattened, apically pointed. ***Tibia*.** With row of short, blunt setae on each side parallel to row of long, fine, simple setae. ***Claw*** with one row of nine or ten denticles; one posterior seta. Otherwise, as typical for subgenus.

***Abdomen*. *Terga*** (Fig. 32f, g). Posterior margin of terga: I smooth, without denticles, II–IX with triangular, pointed denticles.

***Tergalii*** (Fig. 32h). Elongate oval; tracheation well-developed, nearly reaching margins, dark brown; with reddish brown pigmentation around tracheation. Margin with minute denticles and short, fine, simple setae.

***Paraproct*** (Fig. 32i). Posterior margin with prolongation, with split denticles.

***Caudalii*** (Fig. 31i, j). Cerci with 1 or 2 swimming setae per segment in distomedial part. Paracercus with 12 segments.

***Pose of subimaginal gonostyli under larval cuticle*.** Unknown.

**Subimago.** Unknown.

**Imago.** Unknown.

**Egg.** Unknown.

##### Biological aspects.

Larvae living in run or riffles of medium fast flowing lowland rivers (Fig. 38a).

##### Etymology.

The species is named after the Sekawa River system in Vanua Levu, where the type locality is.

##### Distribution.

Fiji: Vanua Levu (Fig. 40c).

#### ﻿ Papuanatula (Fijifiliola) tenebris

Taxon classificationAnimaliaEphemeropteraBaetidae

sp. nov.

95828F6E-A5F6-55A8-9661-0F2B1DE454F2

https://zoobank.org/C3925780-999C-462E-B92A-E7A1F1F9B4D7

Figs 33, 34, 35

##### Material examined.

***Holotype*.** FIJI • larva; Viti Levu, Ba Prov., near Abaca village, Savuione Riv.; 17°40'07"S, 177°32'31"E; 510 m; 24.x.2024; leg. T. Kaltenbach; on slides; GBIFCH01221831, GBIFCH01581923; MZL.

##### Diagnosis.

**Larva**. Following combination of characters distinguishes *P. tenebris* sp. nov. from other species of Fijifiliola subgen. nov.: larva dorsally rather uniform blackish brown; femur on anterior side in basal ½ with dark brown, narrow, longitudinal streak in large, oblong blank (all legs), large dark grey band in distomedial area; labrum dorsally with irregular arrangement of submarginal setae; maxillary palp ~1.5× length of galea-lacinia; tergalii rather large, skewed oval, with brown pigmentation; labial palp segment III conical; paracercus with 20 segments.

##### Description.

**Figure 33.**
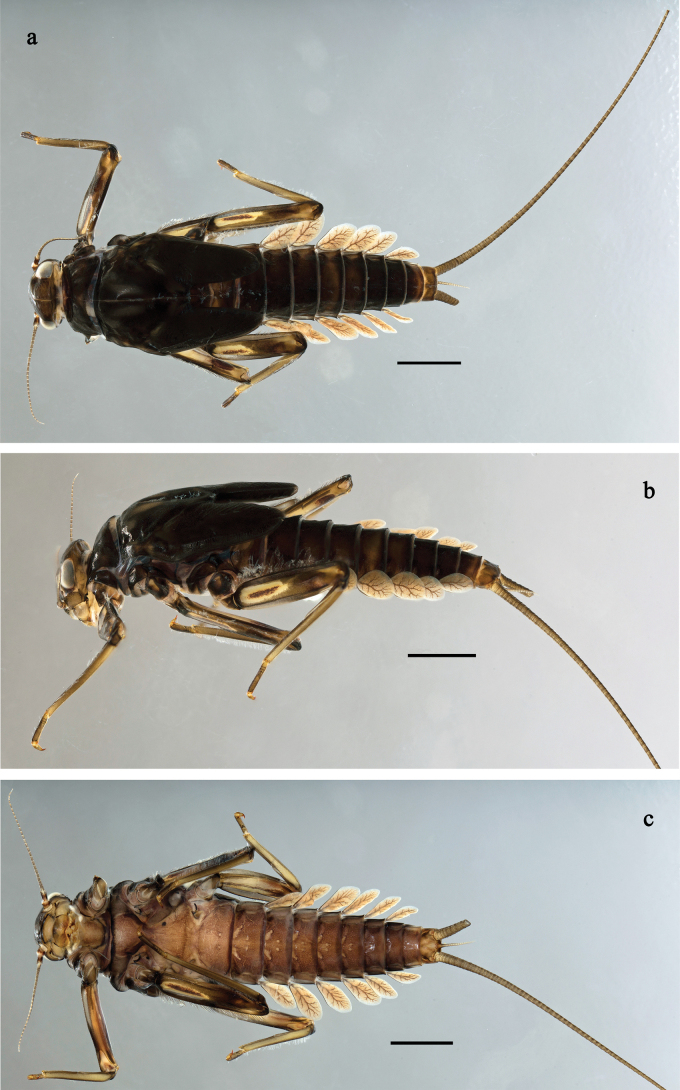
Papuanatula (Fijifiliola) tenebris sp. nov., larva, habitus. a. Dorsal view; b. Lateral view; c. Ventral view. Scale bars: 1 mm.

**Figure 34.**
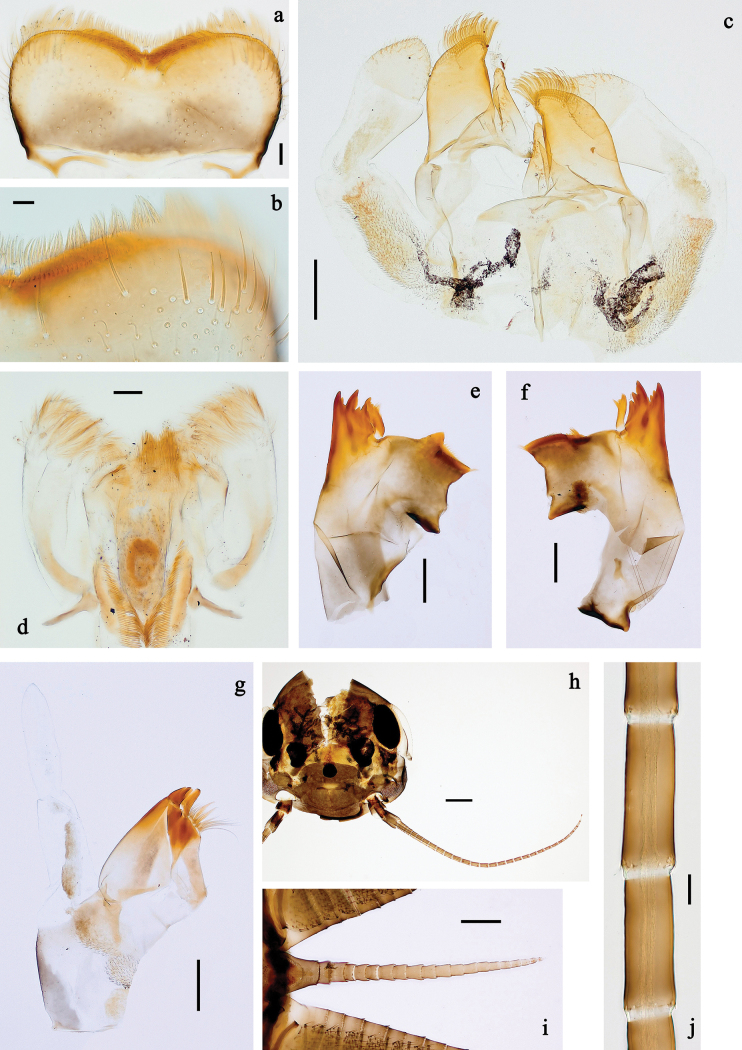
Papuanatula (Fijifiliola) tenebris sp. nov., larva. a, b. Labrum; c. Labium; d. Hypopharynx and superlinguae; e. Left mandible; f. Right mandible; g. Maxilla; h. Head; i. Paracercus; j. Cercus. Scale bars: 100 µm (h); 50 µm (c, e–g); 20 µm (a, d); 10 µm (b, i, j).

**Figure 35.**
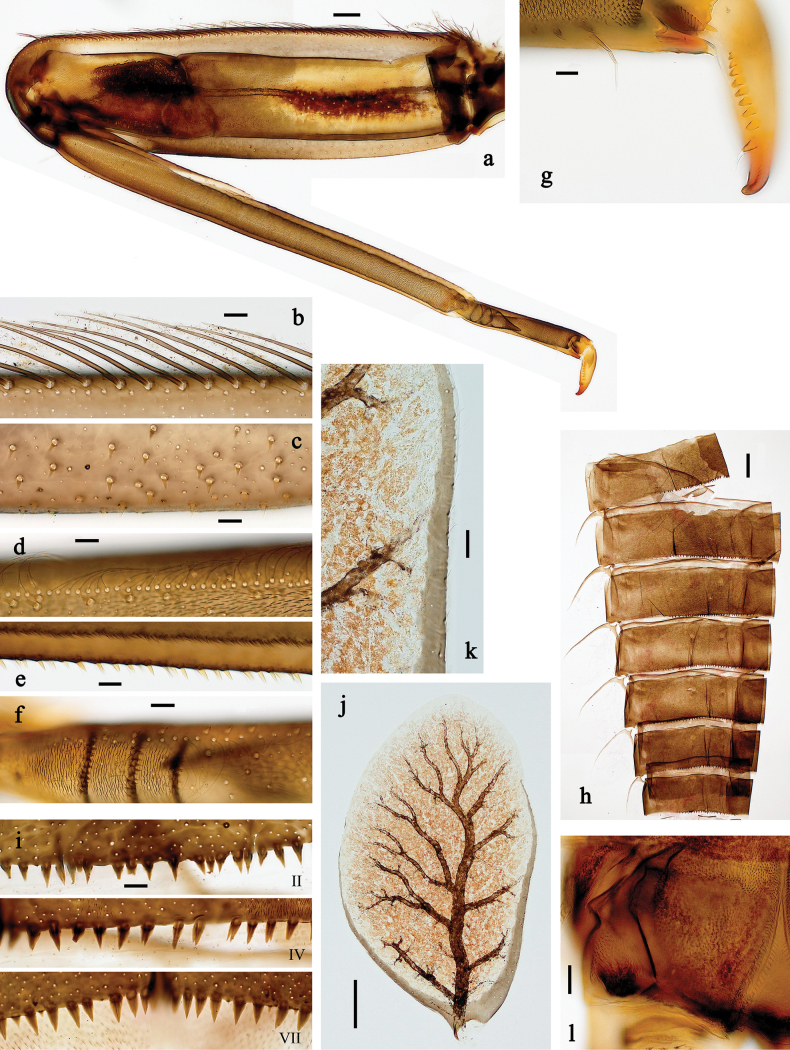
Papuanatula (Fijifiliola) tenebris sp. nov., larva. a. Middle leg; b. Femur, outer margin; c. Femur, inner margin; d. Tibia; e. Tibia, inner margin; f. Tarsus, outer margin; g. Tarsus, claw; h. Abdomen; i. Abdominal terga; j, k. Tergalius IV; l. Paraproct. Scale bars: 100 µm (h); 50 µm (a, j); 10 µm (b–g, i, k, l).

**Larva** (Figs 33–35). Body length 6.4 mm, cerci broken.

***Colouration*** (Fig. 33a–c). Head, thorax and abdomen dorsally rather uniform blackish brown. Femur on anterior side in basal ½ with dark brown, narrow, longitudinal streak in large, oblong blank (all legs), large dark grey band in distomedial area; tibia and tarsus grey. Head, thorax and abdomen ventrally dark grey-brown. Caudalii dark grey.

***Head*. *Antenna*** (Fig. 34h). Length ~1.5× head length. As typical for subgenus.

***Labrum*** (Fig. 34a, b). Length 0.5× maximum width. Dorsally with irregular arrangement of submarginal setae. Otherwise, as typical for subgenus.

***Right mandible*** (Fig. 34f). Margin between prostheca and mola almost straight, with minute denticles. Otherwise, as typical for subgenus.

***Left mandible*** (Fig. 34e). Margin between prostheca and mola almost straight, with minute denticles toward subtriangular process. Otherwise, as typical for subgenus.

***Hypopharynx and superlinguae*** (Fig. 34d). As typical for subgenus.

***Maxilla*** (Fig. 34g). Maxillary palp ~1.5× length of galea-lacinia; palp segment II approx. as long as segment I. Otherwise, as typical for subgenus.

***Labium*** (Fig. 34c). Paraglossa with two spine-like setae on inner, distolateral margin. Labial palp with segment I approx. as long as segments II and III combined. Segment II dorsally with row of six spine-like setae plus additional one or two setae near outer, distolateral margin. Segment III conical, 0.6× length of segment II. Otherwise, as typical for subgenus.

***Legs*** (Fig. 35a–g). Ratio of leg segments: fore leg 1.0:1.0:0.3:0.1, middle leg 1.0:1.0:0.3:0.1 and hind leg 1.0:1.0:0.3:0.1. ***Femur***. Length ~3.5× maximum width. Setae on outer margin long, flattened, pointed. ***Tibia*.** With row of short, blunt setae parallel to row of long, fine, simple setae. ***Claw*** with one row of ten or 11 denticles, distalmost denticle with distance to other denticles; one posterior seta. Otherwise, as typical for subgenus.

***Abdomen*. *Terga*** (Fig. 35h, i). Posterior margin of terga: II–IX with triangular, sharply pointed denticles.

***Tergalii*** (Fig. 35j, k). Rather large, skewed oval; tracheation well-developed, reaching margins, dark brown; brown pigmentation around tracheae. Margin with minute denticles and short, fine, simple setae.

***Paraproct*** (Fig. 35i). Posterior margin with prolongation, with denticles. Cercotractor without denticles.

***Caudalii*** (Fig. 34i, j). Cerci without swimming setae. Paracercus with 20 segments.

***Pose of subimaginal gonostyli under larval cuticle*.** Unknown.

**Subimago.** Unknown.

**Imago.** Unknown.

**Egg.** Unknown.

##### Biological aspects.

**Figure 36.**
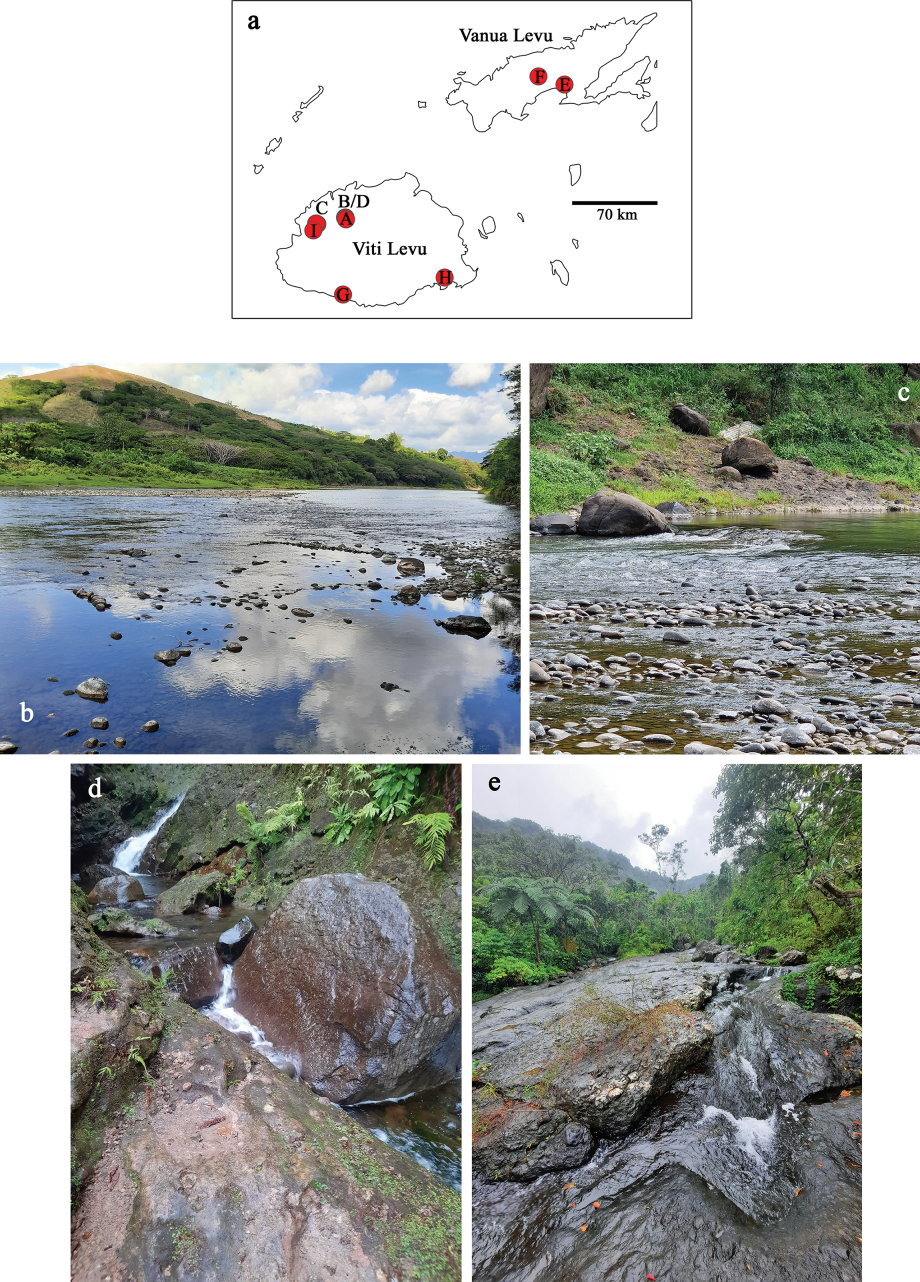
Habitats. a. Overview map; b, c. Ba River near Outback Hotel (locations A, B; lowland river); d. Savuione River, close to beyond the Savuione waterfall, near Abaca village (location C; mountain river); e. Savuione River, downstream of the waterfall, near Abaca village (location C1; mountain river). For characteristics of locations see Table 3.

Local species, larvae living in run of fast flowing mountain rivers with pristine water (Fig. 36e).

**Figure 37.**
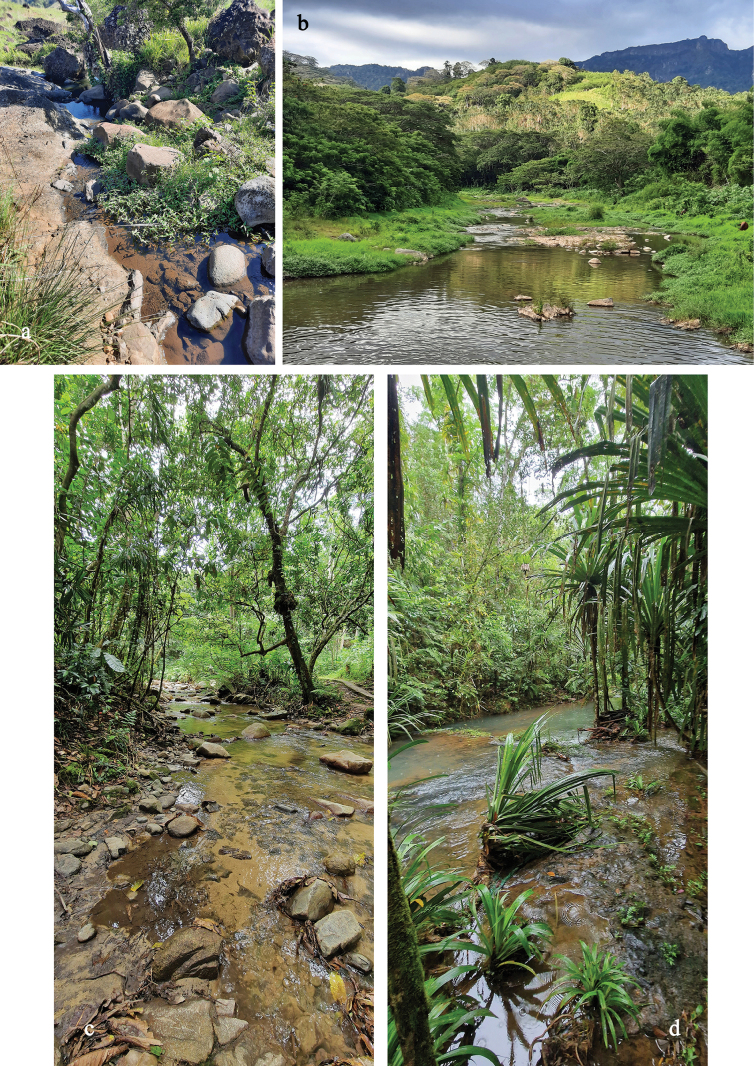
Habitats. a. Creek near Outback Hotel (location D1); b. Sabeto River, near Sabeto (location I; lowland river); c. Biausevu River, near Biausevu (location G); d. Waisila creek, Colo-I-Suva Forest Park (location H). Characteristics of locations see Table 3.

**Figure 38.**
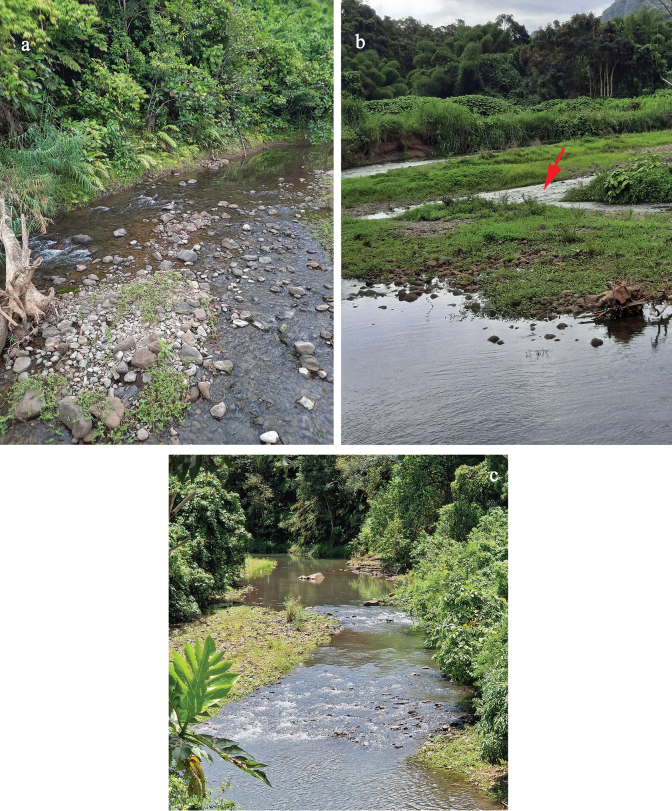
Habitats. a. Tributary to Sekawa River, near Nakawaga (location E; clean lowland river); b. Sekawa River near Nakawaga (location E1; lowland river, disturbed by human activities; many filamentous green algae); c. Seaqaqa River, bridge near Saivou (location F). Characteristics of locations see Table 3.

**Figure 39.**
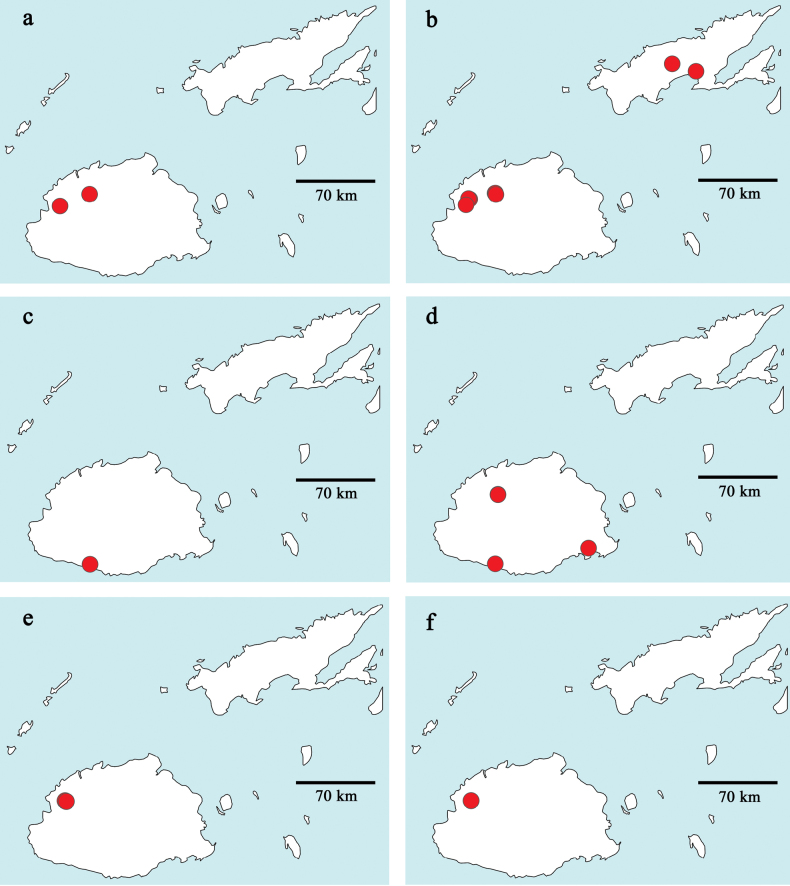
Distribution of described species. a. *Baetis procul* sp. nov. (locations B, I); b. Papuanatula (Fijifiliola) aji sp. nov. (locations A, B, C, C1, D, E, E1, F, I); c. Papuanatula (Fijifiliola) aurantica sp. nov. (location G); d. Papuanatula (Fijifiliola) bula sp. nov. (locations D, D1, G, H); e. Papuanatula (Fijifiliola) claudia sp. nov., Papuanatula (Fijifiliola) flowersi sp. nov. (locations C, C1); f. Papuanatula (Fijifiliola) crussetae sp. nov. (location C).

##### Etymology.

The name refers to the dark appearance of the species, *tenebris* meaning dark in Latin.

##### Distribution.

**Figure 40.**
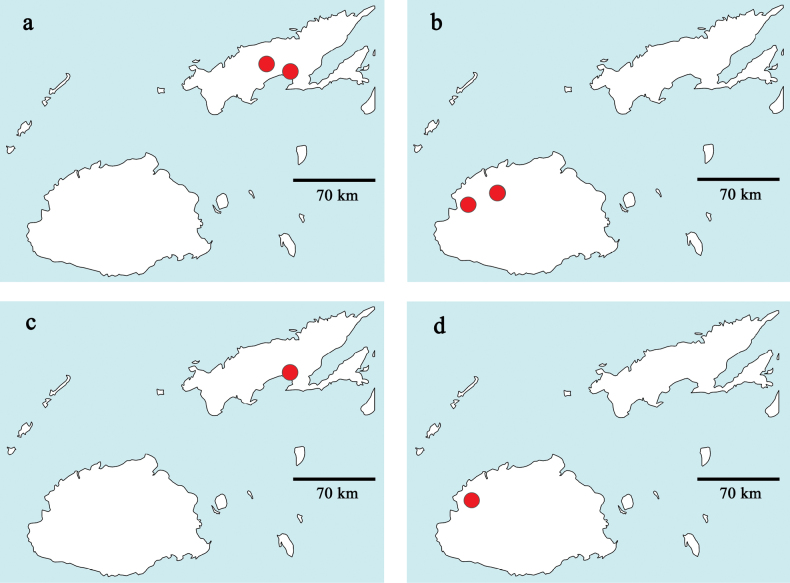
Distribution of described species. a. Papuanatula (Fijifiliola) gattolliati sp. nov. (locations E, F); b. Papuanatula (Fijifiliola) grisea sp. nov. (locations A, B, I); c. Papuanatula (Fijifiliola) sekawa sp. nov. (location E); d. Papuanatula (Fijifiliola) tenebris sp. nov. (location C1).

Fiji: Viti Levu (Fig. 40d).

### ﻿Key to the species of Papuanatula (Fijifiliola) (larvae)

**Table:** 

1	Outer margin of femur with irregular row of medium, spine-like setae (Fig. 20a, b)	**P. (F.) crussetae sp. nov.**
–	Outer margin of femur with regular row of long, flattened setae (Fig. 8a, b)	**2**
2(1)	Labrum dorsally with irregular submarginal row of long, simple setae (Fig. 34b); blackish colour; abdominal tergum II with row of long, pointed spines at posterior margin	**P. (F.) tenebris sp. nov.**
–	Labrum dorsally with regular submarginal arc of long, simple setae (Fig. 7b); colour not blackish; abdominal tergum II not with row of long, pointed spines	**3**
3(2)	Head, thorax, and abdomen dorsally, and femur on anterior side predominantly orange-brown (Fig. 9a, b)	**P. (F.) aurantica sp. nov.**
–	Thorax and abdomen dorsally not orange-brown	**4**
4(3)	Thorax and abdomen dorsally predominantly dark brown; head, legs, and abdominal tergum X orange-brown (Fig. 21a, b)	**P. (F.) flowersi sp. nov.**
–	Not this combination of colour pattern	**5**
5(4)	Abdominal terga IV–VI, posterior 1/2 of IX, and X predominantly yellow-grey, II, III, VII, and VIII rather uniform dark grey; femur on anterior side yellow-grey in distal part (Fig. 6a, b)	**P. (F.) aji sp. nov.**
–	Not this combination of colour pattern	**6**
6(5)	Abdomen dorsally rather uniform grey, laterally with distinct, oblique, blackish markings (Fig. 24a, b); tergalii narrow oblong (Fig. 26i); Vanua Levu	**P. (F.) gattolliati sp. nov.**
–	Abdomen laterally without distinct, blackish markings; tergalii not narrow oblong, but oval; Viti Levu or Vanua Levu	**7**
7(6)	Abdominal terga dark grey with indistinct pattern (Fig. 27a); fore femur on anterior side in basal part with brown streak in wedge-shaped blank (Fig. 27b), distally grey (Fig. 27b); femur on posterior side without distinct marking or colour (Fig. 27c)	**P. (F.) grisea sp. nov.**
–	Abdominal terga with distinct pattern; fore femur on anterior side in basal part with streak in suboval blank (Fig. 12b), distally reddish grey (Fig. 30b); femur on posterior side subdistally with distinct marking (Fig. 15a)	**8**
8(7)	Abdomen dorsally predominantly uniform dark grey, tergum I basally, IX on posterior margin and X entirely orange-brown (Fig. 30a); margin of tergalii with minute denticles and short, simple setae, costal and anal ribs broadly grey, orange-brown pigmentation around tracheae (Fig. 32h); Vanua Levu	**P. (F.) sekawa sp. nov.**
–	Abdomen dorsally with different pattern; margin of tergalii smooth, not broadly grey, no pigmentation around tracheae; Viti Levu	**9**
9(8)	Abdominal terga II and VI–VIII predominantly dark grey, I, III–V and IX, X brighter grey; femur on anterior side in basal part with distinct, dark brown, narrow streak in large blank; femur on posterior side in distal part reddish grey (Fig. 12a–c)	**P. (F.) bula sp. nov.**
–	Abdomen dorsally varying light grey to dark grey, terga IX and X yellowish grey; femur on anterior side with broad, dark brown streak in large blank; femur on posterior side subdistally with broad, distinct, dark brown streak (Fig. 15a–c)	**P. (F.) claudia sp. nov.**

## ﻿Discussion

### ﻿*Baetis procul* sp. nov.

The assignment of the new species to the genus *Baetis* is based on following characters: larvae slender, not dorsoventrally flattened; two pairs of protoptera; simple, stout prostheca on both mandibles; presence of patella-tibial suture on all legs; presence of well-developed femoral patch on all legs; claws with a simple row of denticles; tergalii consisting of a single lamella, usually on abdominal segments I–VII, tergalius I much smaller than others; unmodified paraproct; three caudal filaments (Bauernfeind and Soldán 2012; Yanai et al. 2018). For an assignment to *Baetis*, also the absence of certain characters is important: absence of a carina between antennae; absence of stout setae between prostheca and mola of both mandibles. The imago has double intercalary veins on the forewing, hindwing with two longitudinal veins and a well-developed costal process.

In a global context, *Baetis procul* sp. nov. has morphological similarities with the Holarctic *fuscatus* species group, as originally defined by Müller-Liebenau (1969), and later more detailed by Webb et al. (2018) for North American species: labial palp with curved outer margin, length of segment II < 2× basal width of segment III; pronotum with inverted, dark, U-shaped marking (as in the *flavistriga* complex); dorsal, abdominal pattern very similar to *flavistriga* complex; robust setae absent on antennal scape and pedicel, paraproct, tergalii, and abdominal terga; length of tergalii < 2× width, serrate margins; femur with subquadrangular, dark spot.

Therefore, *Baetis procul* sp. nov. should be included into the subgenus Baetis s. str., as defined slightly differently by both Webb et al. (2018) and Bauernfeind and Soldán (2012), also adopted by Yaagoubi et al. (2023).

The morphologically most similar species is *B. taiwanensis* from Taiwan and Japan. *Baetis procul* sp. nov. is distinguished from *B. taiwanensis* from Taiwan by the following character: larva with abdominal terga I–VIII grey-brown, with distinct sublateral pale areas (Figs 1a, c, 3i; in *taiwanensis* terga III–V much paler than I, II, VI, and VII; Müller-Liebenau 1985: fig. 7). It is distinguished from *B. taiwanensis* from Japan by following characters: femur of larva with irregular, medial brown spot, and subapical, yellow-brown spot (Fig. 3a; *taiwanensis* with rectangular, medial spot and without subapical, yellow-brown spot; Fujitani et al. 2004: figs 9, 16); larval paraproct with five or six large spines (Fig. 3m; *taiwanensis* with several larger and many small spines; Fujitani et al. 2004: fig. 18); hindwing of imago narrow, with concave anal margin (Fig. 4e; *taiwanensis* with wider hindwing, anal margin straight; Fujitani et al. 2004: fig. 3). Judging from descriptions and figures of *B. taiwanensis* from Taiwan (Müller-Liebenau 1985) and Japan (Fujitani et al. 2004), it is not excluded that these are two different species, both different from *B. procul* sp. nov. Further morphologic and genetic investigations may be necessary to clarify their status.

Three female imagos of *Baetis procul* sp. nov. were collected at one site by kick-sampling in the river, together with larvae of *Papuanatula aji* sp. nov. and *P. grisea* sp. nov. (location B). This suggests that the imagos were laying their eggs under water, as it is already known from other species of *Baetis* (Sartori and Brittain 2015; Jacobus et al. 2019). In the lab, it was discovered that none of the three imagos had any egg remaining in the abdomen, which supports the assumption that they were caught just after egg laying under water.

The occurrence of a *Baetis* species in Fiji was unexpected, firstly because there is no species known from New Guinea and the geographic distance to other *Baetis* species in Taiwan and Japan is very large, and secondly, because Flowers (1990) did not mention *Baetis* s. str., but only three species of *Baetis* group *molawinensis* (according to Müller-Liebenau and Hubbard 1985; easily distinguishable from *Baetis* s. str. by their distomedial protuberance on labial palp segment II), which later became *Labiobaetis*. The path of colonisation of the Fiji Islands by *Baetis* remains speculative until more material and studies are available from other archipelagos in the South Pacific, especially from the Solomon Islands. However, several scenarios may be considered. Firstly, a colonisation from New Guinea along the Melanesian arc, which had even more islands in the past and therefore, enabled shorter dispersal distances (Flowers 1990; Ryan 2009); in this case, *Baetis* should be found in New Guinea in the future. New Guinea is very large and many regions are still poorly or not at all investigated for mayflies, including New Ireland (apart from Demoulin 1969). Secondly, a colonisation from Southeast Asia, where *Baetis* is present, via Palau to the Melanesian arc. An ecological study reported the presence of *Baetis* in Palau (Bright 1982). However, this could be also a species of *Labiobaetis*, which was still included into *Baetis* at this point. Finally, one cannot exclude the involvement of human activities in the arrival of *Baetis* on the Fiji Islands.

### ﻿Assignment of Fijifiliola subgen. nov. to Papuanatula, and affinities with other subgenera

Fijifiliola subgen. nov. clearly belongs to the genus *Papuanatula*, according to the diagnosis given in Kaltenbach et al. (2025a): antennal scapes basally narrow, distally broader; labrum much wider than long; labium with glossae much shorter than paraglossae; maxillary palp with two segments; outer side of each femur usually with single regular row of long, hair-like setae; femoral patch absent on all legs; patella-tibial suture usually present on all legs; tibia-tarsal condylus (originally located on outer side) turned to anterior side; anterior (originally outer-anterior) side of each tibia usually with regular row of setae similar to that on femur; anterior (originally outer) side of each tarsus usually with regular row of setae similar to femur and tibia; tarsus usually with conspicuous, long seta subdistally on inner margin; claw with single row of denticles and one or several posterior setae; hind protoptera absent or vestigial; tergalii present on abdominal segments II–VII; paraproct usually with prolongation on proximal margin; cerci usually longer than body length; paracercus strongly reduced or vestigial. However, in Fijifiliola subgen. nov., the regular row of setae on anterior side of tibia is not similar to the one on outer margin of femur, but usually consisting of long, fine, simple setae; a row of similar setae is present along outer margin of tarsus (Fig. 5). These are the main differences to both other subgenera *Papuanatula* s. str. and *Papuafiliola*. Another important difference is the shape of the distomedial protuberance on labial palp segment II: very short and broadly rounded in Fijifiliola subgen. nov., minute or not developed in *Papuanatula* s. str., and well-developed, elongate in *Papuafiliola* (Fig. 5). Further, Fijifiliola subgen. nov. is distinguished from the other two subgenera by a different combination of some characters, as summarised in Fig. 5 and Table 3: shape of labrum is similar to that of *Papuanatula* s. str. (widest in middle part; widest basally in *Papuafiliola*); labrum with dorsal, submarginal arc of simple setae similar to *Papuafiliola*, but a full arc instead of few setae as in *Papuafiliola* (feathered setae in *Papuanatula* s. str.); incisor of both mandibles blade-like enlarged as in *Papuanatula* s. str. (not blade-like in *Papuafiliola*); distalmost seta on inner margin of tarsus much longer than other setae as in *Papuanatula* s. str. (not much longer in *Papuafiliola*). Overall, Fijifiliola subgen. nov. seems to have more morphological similarities with *Papuanatula* s. str. than with *Papuafiliola*, However, these similarities do not justify a judgement on the relationship between the three subgenera of *Papuanatula*.

Based on the apomorphies and the homogeneity of all three subgenera, and their geographic distribution, raising them from subgeneric to generic status could be considered in the future. However, I prefer to remain conservative, waiting for further collections and studies on other archipelagos of the South Pacific.

### ﻿Parthenogenesis and ovoviviparity in Papuanatula (Fijifiliola) subgen. nov.

In one mature female larva of P. (F.) claudia sp. nov., the abdomen was filled with eggs in which the developing larvae could be observed (Fig. 16k, l). It is the first documented case of parthenogenesis in *Papuanatula*. There are male and female larvae in this population, which means that the parthenogenesis is facultative. As the young larvae are already developing in this early phase, the species is most probably also ovoviviparous. Both, parthenogenesis and ovoviviparity are well known in mayflies (Funk et al. 2010; Sartori and Brittain 2015; Jacobus et al. 2019; Liégeois et al. 2021), a comprehensive review is given by Liégeois et al. (2021). Moreover, there is evidence that most, if not all, mayflies, an ancient group with a primitive reproductive system, are facultatively parthenogenetic (Funk et al. 2010). In many Baetidae, ovoviviparity and/or parthenogenesis have also been reported (e.g. *Acerpenna* Waltz & McCafferty, *Alainites* Waltz & McCafferty, *Anafroptilum* Kluge, *Baetis* Leach, *Callibaetis* Eaton, *Centroptilum* Eaton, *Cloeon* Leach, *Labiobaetis*, *Nigrobaetis* Kazlauskas (in Novikova and Kluge), *Procloeon* Bengtsson) (Needham and Murphy 1924; Berner 1941; Sivaramakrishnan et al. 1991; Funk et al. 2010; Gattolliat and Staniczek 2011; Kluge 2016; Liégeois et al. 2021). Parthenogenesis and ovoviviparity may favour successful random colonisation, because it enables virgin females to form populations in new habitats, which may even be bisexual (Funk et al. 2010). Therefore, it is not surprising to find it in species living in remote, isolated environments like islands or archipelagos, as it is the case for Papuanatula (F.) claudia sp. nov. from Fiji and *Labiobaetis paradisus* Gattolliat & Staniczek from Vanuatu (Gattolliat and Staniczek 2011).

### ﻿Genetics

COI barcode sequences were obtained from eight of the ten species of Fijifiliola subgen. nov. (Table 1; Suppl. material 1). The interspecific distances are always rather high (13%–23%, K2P), in line with genetic distances found in *Papuanatula* (incl. *Papuanatula* s. str. and *Papuafiliola*) in New Guinea (10%–25%; Kaltenbach et al. 2025a, b). *Labiobaetis* Novikova & Kluge has also comparable genetic distances in New Guinea (13%–31%; Kaltenbach and Gattolliat 2018, 2021). Ball et al. (2005) reported a mean interspecific, congeneric distance of 18% for mayflies from the United States and Canada.

The intraspecific distances were mostly between 0% and 3%, as expected. An exception is one specimen of *P. aji* sp. nov. from location D with 6%–7% genetic distance to all other locations, whereby, another specimen of the same location has the usual 0%–3%. Isolation cannot be an explanation for this case, but rather the involvement of a pseudogene or other unknown reasons. The second exception is one specimen of *P. bula* sp. nov. from location D1 with 5%–6% genetic distance to the other locations. Here, the larger genetic distance could be explained by a possible isolation from the other locations. Apart from these two exceptions, the species of Fijifiliola subgen. nov. do not show distances of more than the usual 0%–3% between different locations of the same species, even not between the islands Viti Levu and Vanua Levu (*P. aji* sp. nov.). Intraspecific distances of 4%–6% were also reported in some cases for *Labiobaetis* species in New Guinea, Indonesia, and Borneo (Kaltenbach and Gattolliat 2018, 2019, 2020), as well as in aquatic beetles in the Philippines (Komarek and Freitag 2020). Ball et al. (2005) also reported a case with 6% intraspecific distance in a mayfly in North America. Moreover, in a large study on the DNA barcode (COI) of North American Ephemeroptera, nearly 20% of the species included two or three haplotype clusters with more than 5% sequence divergence (Webb et al. 2012). The likely involvement of species complexes underlying these results are discussed in this study. Finally, intraspecific K2P distances of more than 3.5% are not uncommon within Plecoptera as well (Gill et al. 2015; Gattolliat et al. 2016).

### ﻿Distribution and diversity of *Papuanatula*

Based on present data, the genus has a disjunct distribution in New Guinea (incl. New Britain), the island of Sulawesi, and in the Fiji Islands (Kaltenbach et al. 2025a, b; this study). The subgenus Papuanatula s. str. is present in New Guinea and Sulawesi, *Papuafiliola* in New Guinea, and *Fijifiliola* in the Fiji Islands only.

Despite many collection activities and studies on Baetidae in continental Southeast Asia, Indonesia, and the Philippines in recent years, no species of *Papuanatula* was found apart from in Sulawesi. However, the mayfly fauna of islands between Sulawesi and New Guinea (e.g. Ambon, Halmahera) is still very poorly studied. Islands or archipelagos of the South Pacific other than Fiji Islands (e.g., Solomon Islands, Tonga, Samoa) are mostly very poorly or not at all studied and no *Papuanatula* species were reported from there (Tillyard 1928). From Vanuatu, which was studied in more detail, only a very low diversity was reported and no species of *Papuanatula* were recorded (Gattolliat and Staniczek 2011). New Caledonia is an exception and much better studied, with a high diversity of Leptophlebiidae and one species of *Cloeon* of unclear origin, but no species of *Papuanatula* (Peters and Mary 2016; Hrivniak et al. 2023 and citations therein).

The fact that all known species of *Papuanatula* in Fiji belong to the morphologically well-defined subgenus Fijifiliola subgen. nov., which was not discovered anywhere else so far, speaks for one single colonisation by a *Papuanatula* species in the past, with subsequent local radiation. However, the morphology of Fijifiliola subgen. nov. with characters partly similar to *Papuanatula* s. str. and partly to *Papuafiliola* (Fig. 5; Table 3), does not allow an assessment on whether Fijifiliola subgen. nov. is more related to one or the other of these subgenera. Flowers (1990) already recognised the relationship of Fijifiliola subgen. nov. (as New Genus “A”) with two undescribed species from New Guinea and one from New Britain (“*Pseudocloeon* B” from “Bismarck Islands”; Demoulin 1969), which were later described as *Papuanatula* species by Lugo-Ortiz and McCafferty (1999). Flowers (1990) mentioned eight different species, one of them very common (probably *P. aji* sp. nov.) and some occurring in one locality only, based on rich material from 60 different locations on four islands, mostly from the main islands Viti Levu and Vanua Levu. In this study, ten species are described from 12 different sites of nine different rivers on Viti Levu and Vanua Levu. This suggests that there are probably more than eight species in the material studied by Flowers (1990), which may be revealed by further taxonomic investigation of this material, and that there is a total of more than the ten species described in this study. This is a remarkable diversity for this archipelago, which could be explained by the highly structured environment (islands and mountainous areas), providing opportunities for random colonisation, followed by isolation and speciation. Allopatry was identified as main mechanism of diversification of the megadiverse Dytiscidae genus *Exocelina* Balke, 1998 in New Guinea (see discussion in Kaltenbach et al. 2025a; Toussaint et al. 2013, 2014), and could be also the driver of the diversity in the Fiji Islands.

Further collections on the main islands Viti Levu and Vanua Levu, especially in higher parts of mountainous areas, and on yet unexplored islands such as Taveuni (third biggest island), will most probably uncover further new species of Papuanatula (Fijifiliola) subgen. nov. in the Fiji Islands.

## Supplementary Material

XML Treatment for Baetis procul

XML Treatment for Fijifiliola subgen. nov.

XML Treatment for Papuanatula (Fijifiliola) aji

XML Treatment for Papuanatula (Fijifiliola) aurantica

XML Treatment for Papuanatula (Fijifiliola) bula

XML Treatment for Papuanatula (Fijifiliola) claudia

XML Treatment for Papuanatula (Fijifiliola) crussetae

XML Treatment for Papuanatula (Fijifiliola) flowersi

XML Treatment for Papuanatula (Fijifiliola) gattolliati

XML Treatment for Papuanatula (Fijifiliola) grisea

XML Treatment for Papuanatula (Fijifiliola) sekawa

XML Treatment for Papuanatula (Fijifiliola) tenebris

## ﻿Additional information

### Use of AI

No use of AI was reported.

### Author ORCIDs

Thomas Kaltenbach https://orcid.org/0000-0001-8052-0388

### Data availability

All of the data that support the findings of this study are available in the main text or Supplementary Information.

## Supplementary materials

Supplementary material 1 Supportive Data: Genetic distance (COI, K2P; intraspecific distances in bold) Data type xls https://binary.pensoft.net/file/1462934This dataset is made available under the Open Database License (http://opendatacommons.org/licenses/odbl/1.0/). The Open Database License (ODbL) is a license agreement intended to allow users to freely share, modify, and use this Dataset while maintaining this same freedom for others, provided that the original source and author(s) are credited.Author: Thomas Kaltenbach
